# The trispecific DARPin ensovibep inhibits diverse SARS-CoV-2 variants

**DOI:** 10.1038/s41587-022-01382-3

**Published:** 2022-07-21

**Authors:** Sylvia Rothenberger, Daniel L. Hurdiss, Marcel Walser, Francesca Malvezzi, Jennifer Mayor, Sarah Ryter, Hector Moreno, Nicole Liechti, Andreas Bosshart, Chloé Iss, Valérie Calabro, Andreas Cornelius, Tanja Hospodarsch, Alexandra Neculcea, Thamar Looser, Anja Schlegel, Simon Fontaine, Denis Villemagne, Maria Paladino, Dieter Schiegg, Susanne Mangold, Christian Reichen, Filip Radom, Yvonne Kaufmann, Doris Schaible, Iris Schlegel, Christof Zitt, Gabriel Sigrist, Marcel Straumann, Julia Wolter, Marco Comby, Feyza Sacarcelik, Ieva Drulyte, Heyrhyoung Lyoo, Chunyan Wang, Wentao Li, Wenjuan Du, H. Kaspar Binz, Rachel Herrup, Sabrina Lusvarghi, Sabari Nath Neerukonda, Russell Vassell, Wei Wang, Julia M. Adler, Kathrin Eschke, Mariana Nascimento, Azza Abdelgawad, Achim D. Gruber, Judith Bushe, Olivia Kershaw, Charles G. Knutson, Kamal K. Balavenkatraman, Krishnan Ramanathan, Emanuel Wyler, Luiz Gustavo Teixeira Alves, Seth Lewis, Randall Watson, Micha A. Haeuptle, Alexander Zürcher, Keith M. Dawson, Daniel Steiner, Carol D. Weiss, Patrick Amstutz, Frank J. M. van Kuppeveld, Michael T. Stumpp, Berend-Jan Bosch, Olivier Engler, Jakob Trimpert

**Affiliations:** 1 Spiez Laboratory, Austrasse, Spiez, Switzerland; 2 Institute of Microbiology, University Hospital Center and University of Lausanne, Lausanne, Switzerland; 3 Department Biomolecular Health Sciences, Division Infectious Diseases & Immunology - Virology section, Faculty of Veterinary Medicine, Utrecht University, Utrecht, Netherlands; 4 Cryo-Electron Microscopy, Bijvoet Center for Biomolecular Research, Department of Chemistry, Faculty of Science, Utrecht University, Utrecht, Netherlands; 5 Molecular Partners AG, Zurich-Schlieren, Switzerland; 6 Materials and Structural Analysis, Thermo Fisher Scientific, Eindhoven, Netherlands; 7 Binz Biotech Consulting, Zug, Switzerland; 8 Laboratory of Immunoregulation, Division of Viral Products, Center for Biologics Evaluation and Research, US Food and Drug Administration, Silver Spring, MD USA; 9 Freie Universität Berlin, Institut für Virologie, Berlin, Germany; 10 Freie Universität Berlin, Institut für Tierpathologie, Berlin, Germany; 11 Novartis Institutes for BioMedical Research, PK Sciences, Cambridge, MA USA; 12 Novartis Institutes for BioMedical Research, Preclinical Safety, Basel, Switzerland; 13 Novartis Pharma AG, Basel, Switzerland; 14 Max-Delbrück-Center for Molecular Medicine in the Helmholtz Association, Berlin, Germany

**Keywords:** Cryoelectron microscopy, Antiviral agents

## Abstract

The emergence of severe acute respiratory syndrome coronavirus 2 (SARS-CoV-2) variants with potential resistance to existing drugs emphasizes the need for new therapeutic modalities with broad variant activity. Here we show that ensovibep, a trispecific DARPin (designed ankyrin repeat protein) clinical candidate, can engage the three units of the spike protein trimer of SARS-CoV-2 and inhibit ACE2 binding with high potency, as revealed by cryo-electron microscopy analysis. The cooperative binding together with the complementarity of the three DARPin modules enable ensovibep to inhibit frequent SARS-CoV-2 variants, including Omicron sublineages BA.1 and BA.2. In Roborovski dwarf hamsters infected with SARS-CoV-2, ensovibep reduced fatality similarly to a standard-of-care monoclonal antibody (mAb) cocktail. When used as a single agent in viral passaging experiments in vitro, ensovibep reduced the emergence of escape mutations in a similar fashion to the same mAb cocktail. These results support further clinical evaluation of ensovibep as a broad variant alternative to existing targeted therapies for Coronavirus Disease 2019 (COVID-19).

## Abstract (web summary)

Multiple variants of SARS-CoV-2 are inhibited by a trispecific DARPin.

## Main

Antiviral drugs against SARS-CoV-2 have relied primarily on small molecules and neutralizing mAbs. However, the efficacy of existing drugs is threatened by the potential emergence of escape variants via selective evolutionary pressure. To address this concern, antibody cocktails have been generated to provide broader protection against variants^1–3^. Recent data showed that two treatments of two antibodies administered as a cocktail were unlikely to be active against the Omicron BA.1 variant, leading the US Food and Drug Administration to revise their authorization for use. These concerns emphasize the need to develop alternative therapeutic modalities with broad variant activity.

DARPins are a novel class of therapeutics that are being developed in ophthalmology and oncology^4,5^. They are structurally different from antibodies and consist of a single chain of linked DARPin binding domains. Here we report the application of the DARPin platform^6^ to generate ensovibep, an anti-SARS-CoV-2 multispecific DARPin clinical candidate^7–9^. The smaller size of ensovibep (85 kDa), compared to ~150 kDa of a mAb, in conjunction with high thermal stability^7^, high production yields^7^ and demonstrated high protection against viral variants, makes this molecule an attractive alternative to other treatments.

The main mutations identified in SARS-CoV-2 localize to the spike protein, a metastable pre-fusion trimer on the viral membrane that mediates virus entry into the host cell. The spike protein comprises multiple functional subunits: S1, including the N-terminal domain (NTD) and the receptor-binding domain (RBD), responsible for interaction with the angiotensin-converting enzyme 2 (ACE2) host receptor^10–13^, and the S2 subunit, which is responsible for virus–host cell membrane fusion via irreversible conformational changes^14–17^. By the end of 2021, many viral lineages had been identified and designated as variants of interest (VOIs) or variants of concern (VOCs) based on their associated increased risk to public health—among them, the most recent lineage of Omicron, B.1.1.529 (refs. ^18–30^).

Many variants harbor mutations in the RBD domain of the spike protein, mainly in the ACE2-binding site (for example, K417T/N, N439K, L452R, E484K/Q and N501Y). These mutations have been linked to either increasing the affinity to the human ACE2 receptor (N439K and N501Y) and, therefore, transmissibility and/or facilitating immune escape (K417T/N, L452R and E484K/Q)^18,19,23–25,31–33^. In particular, the E484K substitution has been shown to play a key role in attenuating antibody potency, according to a study analyzing clinical-stage therapeutic antibodies^20^.

Using cryo-electron microscopy (cryo-EM) analysis, we provide an explanation for ensovibep-mediated neutralization of SARS-CoV-2. Three DARPin domains can simultaneously target the receptor-binding ridge (RBR) on each RBD of the spike trimer, locking the spike in an open conformation and occluding the ACE2-binding site. Owing to the cooperative binding of this trispecific design, ensovibep confers high protection against a panel of relevant spike mutants as well as all frequent SARS-CoV-2 variants identified to date. We show, in a viral passaging experiment, that the protection provided by ensovibep against the development of viral escape mutants is equivalent to that of a clinically evaluated mAb cocktail^1,34,35^. We further demonstrate high in vivo efficacy in a therapeutic Roborovski dwarf hamster model where ensovibep protects against severe disease induced by SARS-CoV-2 (ref. ^36^).

## Results

### Structural basis for SARS-CoV-2 spike neutralization by ensovibep

**Fig. 1:**
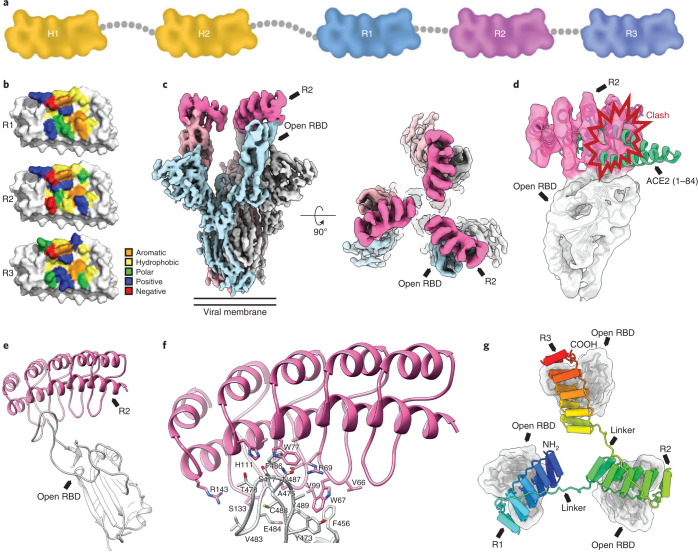
Structural modeling of ensovibep. **a**, Schematic overview of the ensovibep construct. Protein linkers are depicted as gray dashed lines, and the half-life-extending HSA-binding monovalent DARPins (H1 and H2) are colored yellow. **b**, Surface representations of the three monovalent DARPin molecules binding to the RBD, with the amino acid residues in the paratope colored according to their biophysical characteristics as indicated. **c**, cryo-EM density for the SARS-CoV-2 spike ectodomain in complex with the RBD-targeting monovalent DARPin R2, shown as two orthogonal views. The DARPin density is colored magenta, and the three spike protomers are colored light blue, gray and pale pink. **d**, Zoomed-in view of an RBD bound to DARPin R2 with the cryo-EM density shown semi-transparent. The atomic coordinates for the fitted open RBD (PDB ID: 6XCN) and the DARPin model are overlaid. The atomic coordinates for residues 1–84 of the RBD-bound ACE2 (PDB ID: 6M0J), colored green, are superimposed. **e**, Pseudo-atomic model of the monovalent DARPin R2 in complex with the RBD, colored pink and gray, respectively. **f**, Zoomed-in view of the interface between monovalent DARPin R2 and RBD. **g**, Proposed model of the three covalently linked RBD-targeting monovalent DARPin molecules of ensovibep bound to the trimeric spike protein RBD domains. The three DARPin domains are shown in a rainbow color scheme from the N terminus (blue) to the C terminus (red).

Ensovibep comprises two human serum albumin (HSA)-binding DARPin domains for in vivo half-life extension^9^ and three RBD-binding DARPin domains, R1, R2 and R3, with picomolar affinity for their target (Fig. 1a and Supplementary Fig. 1). R1, R2 and R3 are sequence related and target a common epitope (Fig. 1b and Supplementary Figs. 2 and 3), but they are not fully identical in their paratopes (Supplementary Fig. 2). This allows the trispecific molecule to cover a potency loss if a monovalent binder is susceptible to a mutation. An advantage of this design is that a single ensovibep molecule can bind all three RBDs of the trimeric spike protein in a cooperative manner to improve potency (Supplementary Fig. 3).

To understand how ensovibep neutralizes SARS-CoV-2, we performed cryo-EM analysis on the S-ectodomain incubated with DARPin R2. After a 15-second incubation, three-dimensional (3D) classification revealed that 65% of the S-ectodomains were fully closed, 20% had two open RBDs and 15% had three open RBDs (Supplementary Figs. 4a and 5a and Supplementary Table 1). Density consistent with the DARPin molecule was present on the RBR on each open RBD. After a 60-second incubation, 66% of S-ectodomains had three DARPin-bound, open RBDs (Supplementary Fig. 5b), and 18% had one DARPin-bound RBD trapped in a partially closed conformation (Supplementary Figs. 5b and 6a,b). This demonstrates that DARPin R2 binding prevents closure of the RBD through a previously described ratcheting mechanism^37^. Subsequent 3D refinement produced a 4.2-Å-resolution map (Fig. 1c and Supplementary Fig. 4c–e), and, after focused refinement of the RBD region, the map quality was sufficient to assign the pose of DARPin R2, which binds with its N-terminus orientated toward the spike three-fold symmetry axis (Fig. 1c). The concave paratope covers the RBR, preventing ACE2 binding through steric hindrance (Fig. 1d). Guided by the cryo-EM data, molecular docking experiments were performed between the RBD and DARPin R2. The predicted interface area is ~700 Å^2^, and the key epitope residues F456, Y473, F486, N487 and Y489 form an interface of hydrophobic interactions and hydrogen bonds with the DARPin domain (Fig. 1e,f). Because R1, R2 and R3 share a similar paratope composition and architecture, we could conceptually model the three RBD binders of ensovibep bound to the fully open S-ectodomain (Fig. 1g). This demonstrated that the linkers would permit simultaneous binding of all three DARPin modules, allowing high avidity of ensovibep (Supplementary Fig. 1). These data suggest that ensovibep inhibits SARS-CoV-2 by blocking ACE2 binding, as observed for several SARS-CoV-2-neutralizing antibodies^37,38^.

### Ensovibep is highly potent against SARS-CoV-2 variants

**Fig. 2:**
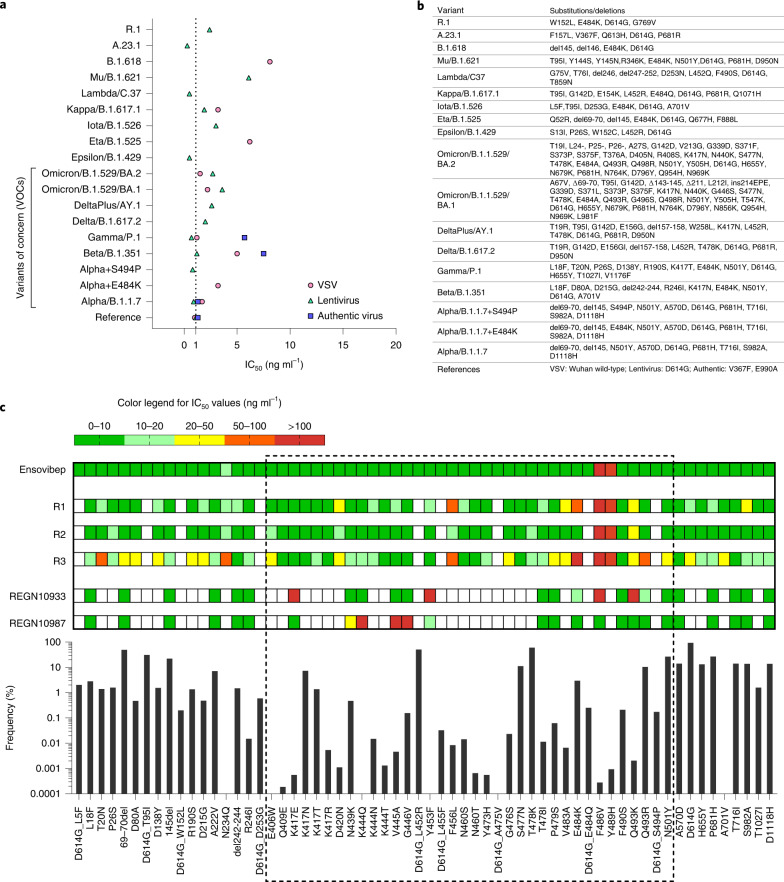
Potency of ensovibep in neutralizing different SARS-CoV-2 variants. **a**, Graph reporting IC_50_ values (ng ml^−1^) for ensovibep measured in neutralization assays performed with lentivirus-based or VSV-based pseudoviruses or authentic viruses for the variants indicated. Reference variant is the Wuhan strain for VSV-based pseudovirus, a D614G variant for the lentivirus-based pseudovirus or a patient isolate from the early pandemic for the authentic virus. **b**, Table of the residues modified in the SARS-CoV-2 spike protein for the different variants tested compared to the Wuhan strain. **c**, Graph with global frequencies of point mutations in the spike protein of SARS-CoV-2 according to the GISAID database (as of February 2022), including a heat map table with IC_50_ values for ensovibep, R1, R2, R3, REGN10933 and REGN10987 for all point mutations tested (VSV-based and lentivirus-based pseudovirus assays). Dashed box: mutations in RBD. White fields: data not available.

**Extended Data Fig. 1:**
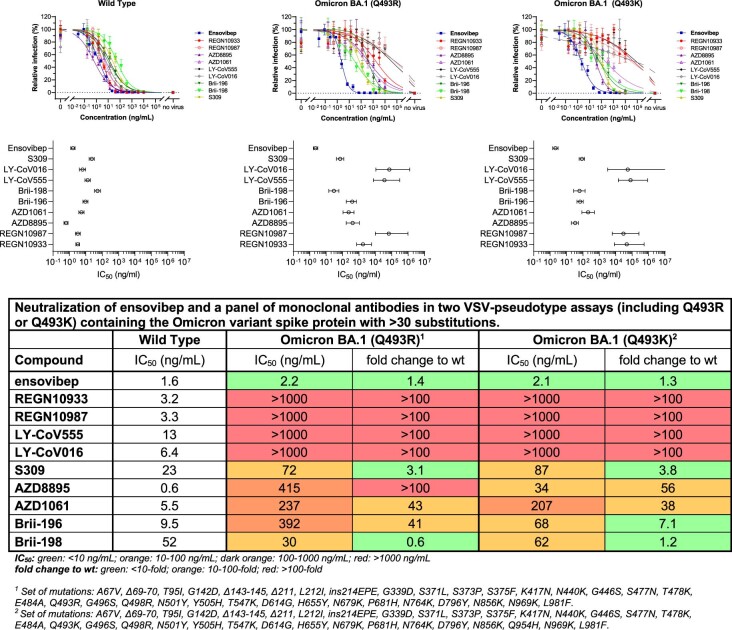
Neutralization of Omicron BA.1 SARS-CoV-2 Variant by Antivirals. Titration curves (mean ± SEM) and IC_50_ values (mean ± CI at 95%) for VSV-pseudotype neutralization assays with wild-type and two different Omicron BA.1 variant spike proteins containing either an arginine or a lysine in position Q493. Ensovibep was tested together with a panel of clinically validated monoclonal antibodies. The table provides the numeric IC50 values as well as the fold change towards the wild-type values.

To assess neutralization of ensovibep against the wild-type SARS-CoV-2 and emerging variants, we used vesicular stomatitis virus (VSV)-based as well as lentivirus-based pseudoviruses displaying the SARS-CoV-2 wild-type or mutant spike protein. In addition, we tested authentic SARS-CoV-2 variants for the Wuhan reference and for lineages B.1.1.7, B.1.351 and P.1. In these settings, ensovibep is able to neutralize the wild-type strain with a half-maximal inhibitory concentration (IC_50_) of ~1 ng ml^−1^ (Fig. 2a). This high neutralization efficacy is retained in most VOCs and VOIs, such as lineage B.1.1.7/Alpha, including also E484K or S494P, B.1.351/Beta, P.1/Gamma, B.1.617.2/Delta, AY.2/Delta Plus, C.37/Lambda, B.1.621/Mu and B.1.1.529/Omicron BA.1 and BA.2. (Fig. 2a,b, Supplementary Table 2 and Supplementary Fig. 7). The neutralization potencies of ensovibep remain within ten-fold difference from the reference virus, with IC_50_ values in the single-digit ng ml^−1^ range, even against variants that were demonstrated to be refractory to immunization, such as Beta, Gamma, Delta, Delta Plus and Omicron variants BA.1 and BA.2 (refs. ^33,39–41^). In contrast, many of the tested clinically relevant mAbs demonstrated a major loss in neutralization (Extended Data Fig. 1 and Supplementary Fig. 8).

Using pseudovirus neutralization assays, we also evaluated the influence of single mutations on the neutralization potency of ensovibep (Supplementary Table 3), the monovalent DARPin R2 and the reference mAbs REGN10933 and REGN10987. The panel included mutations present in VOCs and VOIs, frequent mutations or mutations located within the binding epitope of ensovibep. In contrast to the single mAbs, ensovibep protected well against most point mutations tested, with the only exception of substitutions at F486 and Y489, which affect all monovalent RBD binders incorporated in ensovibep (Fig. 2c). In line, structural analysis and modeling identify F486 and Y489 as core interacting residues for the three RBD binders^7^ (Fig. 1b,f). Consequently, the mutations F486V and Y489H destabilize the spike binding of the entire trispecific ensovibep molecule to the spike protein. However, F486, N487 and Y489 are also critical residues for the interaction between the RBD and ACE2, and their mutation reduces infectivity as demonstrated in pseudovirus experiments (Supplementary Fig. 9). The functional importance of F486 and Y489 is reflected by a low frequency of naturally occurring substitutions at these sites (Fig. 2c). A reduction of potency of ensovibep from one-digit to double-digit ng ml^−1^ IC_50_ values was also observed for mutation N234Q. This residue is located outside of the RBD-binding region of ensovibep. This minor effect could be related to the loss of the conserved glycosylation site at N234Q, favoring the kinetics of the down-conformation of the RBD domain, reducing binding of ensovibep and ACE2 to the RBD, both binding only the RBD up-conformation^42^.

Ensovibep retains potency against spike proteins carrying mutations at locations where the single DARPin domains partially lose activity, such as E484K and Q493K/R. We hypothesize that the cooperative binding in combination with the complementarity of the three RBD binders provide an enhanced resistance to spike mutations.

#### Live SARS-CoV-2 under therapeutic pressure of antivirals

SARS-CoV-2 escape mutants may arise under therapeutic pressure^3,43^. Using a viral passaging model, we compared the risk of mutational escape from therapeutic pressure for ensovibep to that of the monovalent R2 domain, mAbs REGN10933 and REGN10987 (individually or as a mixture) and mAb S309.

**Fig. 3:**
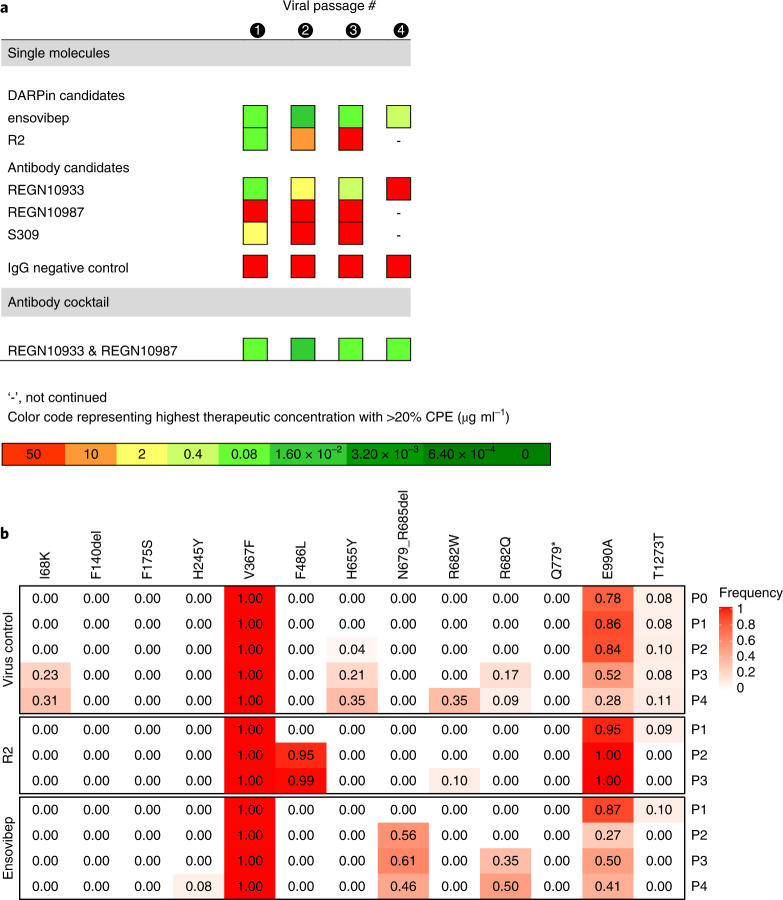
Protection against SARS-CoV-2 escape mutations generated over four viral passages. **a**, Tabular representation of the CPEs induced by SARS-CoV-2 cultured in the presence of increasing concentrations of monovalent DARPin binder R2, multispecific DARPin antiviral ensovibep and the antibody antivirals REGN10933, REGN10987 and S309 or a cocktail of REGN10933 and REGN10987 through passage 1 to passage 4. Color code represents the highest concentration showing ≥20% CPE, for which the culture supernatants were passaged to the next round and deep sequenced for the identification of potential escape mutations. **b**, Identification of escape mutations in viral passages using deep sequencing. SARS-CoV-2 virus was serially passaged with the monovalent DARPin binder R2 and ensovibep. To identify putative escape mutations in the spike protein, RNA was extracted and sequenced from the supernatant of wells with the greatest selective pressure showing a substantial CPE. All variants in the spike protein relative to the reference genome (NC_045512.2) are shown. Passage 0 of the virus control corresponds to the inoculum used for all experiments. The color of the fields is proportional to the fraction of the reads containing the respective variant (red = 1.0, white = 0.0).

A French SARS-CoV-2 isolate (S:V367F/S:E990A) was passaged with increasing concentrations of antivirals (Fig. 3a,b). Escape variants were selected by passaging the supernatant of cultures showing virus-induced cytopathic effect (CPE) for the highest therapeutic concentration onto fresh cells while maintaining the therapeutic pressure (Supplementary Fig. 10). After the first incubation cycle of 4 days (passage 1), ensovibep, DARPin R2, REGN10933 and the antibody mixture conferred protection at the same concentration of 0.4 µg ml^−1^. S309 was less efficient, requiring a higher concentration (10 µg ml^−1^) for protection, and REGN10987 was not protective up to the highest tested concentration of 50 µg ml^−1^. Under continuous selective pressure through passage 2 to passage 4, DARPin R2 and the individual mAbs S309 and REGN10933 lost the capacity to protect cells, whereas ensovibep and the mAb cocktail protected cells throughout the four passages (Fig. 3a).

To identify putative escape mutations of the DARPins, RNA was deep sequenced from the highest antiviral concentration of each passage showing CPE (Fig. 3b). Mutations were found near the spike protein cleavage site (H655Y, N679_R685del, R682W and R682Q), which are likely related to adaptations to the experimental cell system and not escape mutations^36,37^. A potential escape mutation, F486L, was identified for the monovalent R2 but not for ensovibep up to round four. Still, F486 mutations were shown to influence the potency of ensovibep (Fig. 2c).

#### In vivo efficacy of ensovibep in a COVID-19 hamster model

We employed the Roborovski dwarf hamster, a species susceptible to severe COVID-19-like illness^36^, to test the in vivo efficacy of ensovibep and to compare it to the REGN10933 and REGN10987 antibody mixture. Moreover, evaluation of the virological and histopathological outcome of infection enabled comparison across a variety of relevant parameters.

We first aimed to determine in vivo protection conferred by ensovibep against a SARS-CoV-2 wild-type isolate. Initially, we determined both dose and time dependency of treatment efficacy based on clinical and virological parameters (see Methods for detailed descriptions). The course of disease in Roborovski dwarf hamsters is rapid, with animals developing severe disease within 48 hours of infection. We, thus, considered 24 hours post-infection (p.i.) as the latest possible intervention timepoint. Both dose and early administration of ensovibep were found to positively affect the outcome of infection, also evidenced by markedly reduced virus loads in the respiratory tract of treated animals (Supplementary Fig. 11).

**Fig. 4:**
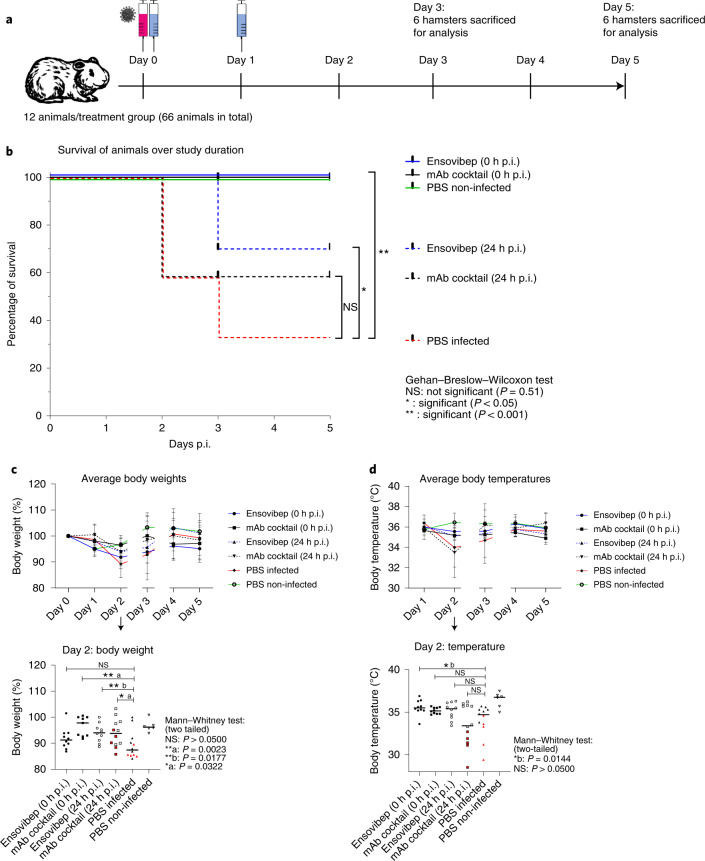
Clinical parameters of SARS-COV-2-infected and treated Roborovski dwarf hamsters. **a**, Design of the Roborovski dwarf hamster study. Animals were infected on day 0 with 10^5^ PFU of SARS-CoV-2 Alpha (B.1.1.7) variant. Treatment was administered either directly after infection (0 hours p.i.) or 1 day after infection (24 hours p.i). For each treatment group, 12 animals were injected i.p. with 10 mg kg^−1^ of ensovibep, 10 mg kg^−1^ of mAb cocktail (5 m kg^−1^ of REGN10933 and 5 mg kg^−1^ of REGN10987) or PBS (placebo). Additionally, a group of six non-infected and non-treated control animals was included as comparators for the infected and treated groups. Daily measurement of body weights and temperatures as well as observation of clinical symptoms was undertaken. Animals were sacrificed on day 3 or day 5 p.i. or immediately once an individual animal reached a defined humane endpoint. **b**, Survival of animals for 5 days p.i. Animals that had to be euthanized according to defined humane endpoints were considered as non-survived. Body weights (**c**) and body temperatures (**d**) throughout the study duration. Data points show mean ± s.d. of the following number of animals analyzed per treatment group at 0/1/2/3/4/5 days p.i.: ensovibep 0 hours: *n* = 10/10/10/10/5/5; mAb cocktail 0 hours: *n* = 9/9/9/9/5/5; ensovibep 24 hours: *n* = 10/10/10/10/5/5; mAb cocktail 24 hours: *n* = 12/12/12/7/6/6; placebo, infected: *n* = 12/12/12/7/4/4; placebo, non-infected: *n* = 6/6/6/6/3/3. Some animals were excluded from the final analysis due to low drug exposure, likely due to a failure of i.p. injections. These animals were excluded from all analyses. Lines connecting dots are interrupted for any change in animal numbers between consecutive days. Because a considerable number of animals in the mAb cocktail and placebo groups reached defined humane endpoints by day 2 p.i., this day is zoomed-in. Red symbols: animals taken out of the study at day 2 due to severe clinical symptoms. Data are represented by the median and values for individual animals. Number of biologically independent animals: ensovibep 0 hours: *n* = 10; mAb cocktail 0 hours: *n* = 9; ensovibep 24 hours: *n* = 10; mAb cocktail 24 hours: *n* = 12; placebo, infected: *n* = 12; placebo, non-infected: *n* = 6. NS, not significant.

**Fig. 5:**
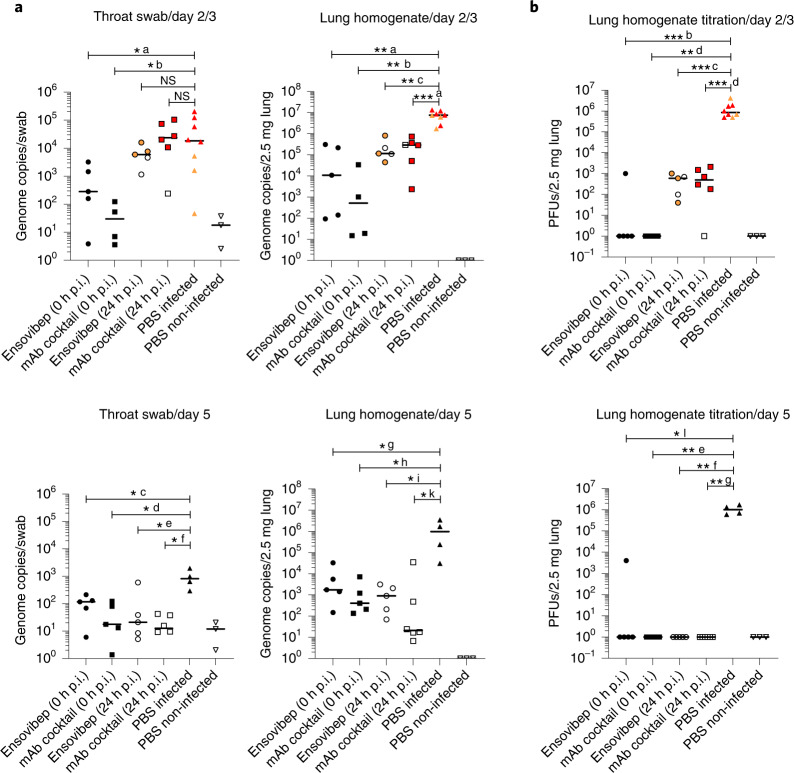
Virology of SARS-COV-2-infected and treated Roborovski dwarf hamsters. **a**, qPCR analysis of virus gRNA copy numbers in oropharyngeal swabs and lung homogenates at day 2/3 or day 5 p.i. **b**, Titration of replication-competent virus from lung homogenates as plaque assay on Vero E6 cells at day 2/3 or day 5 p.i. Red symbols: animals taken out of the study at day 2 due to severe clinical symptoms. Orange symbols: animals taken out of the study at day 3 due to severe clinical symptoms. Data are represented by the median and values for individual animals. Number of animals analyzed per treatment group at day 2/3; day 5 p.i.: ensovibep 0 hours: *n* = 5; 5 / mAb cocktail 0 hours: *n* = 4; 5 / ensovibep 24 hours: *n* = 5; 5 / mAb cocktail 24 hours: *n* = 6; 6 / placebo, infected: *n* = 8; 4 / placebo, non-infected: *n* = 3; 3. Some animals were excluded from the final analysis due to low drug exposure, likely due to a failure of i.p. injections. These animals were excluded from all analyses. Statistics: two-tailed Mann–Whitney test. *P* values: not significant (NS) = *P* > 0.05; *a: 0.0295; *b: 0.0162; *c: 0.0159; *d: 0.0159; *e: 0.0317; *f: 0.0100; *g: 0.0317; *h: 0.0159; *i: 0.0159; *k: 0.0190; *l: 0.0159; **a: 0.0016; **b: 0.0040; **c: 0.0016; **d: 0.0159; **e: 0.0079; **f: 0.0079; **g: 0.0048; ***a: 0.0007; ***b: 0.0008; ***c: 0.0008; ***d: 0.0007.

**Fig. 6:**
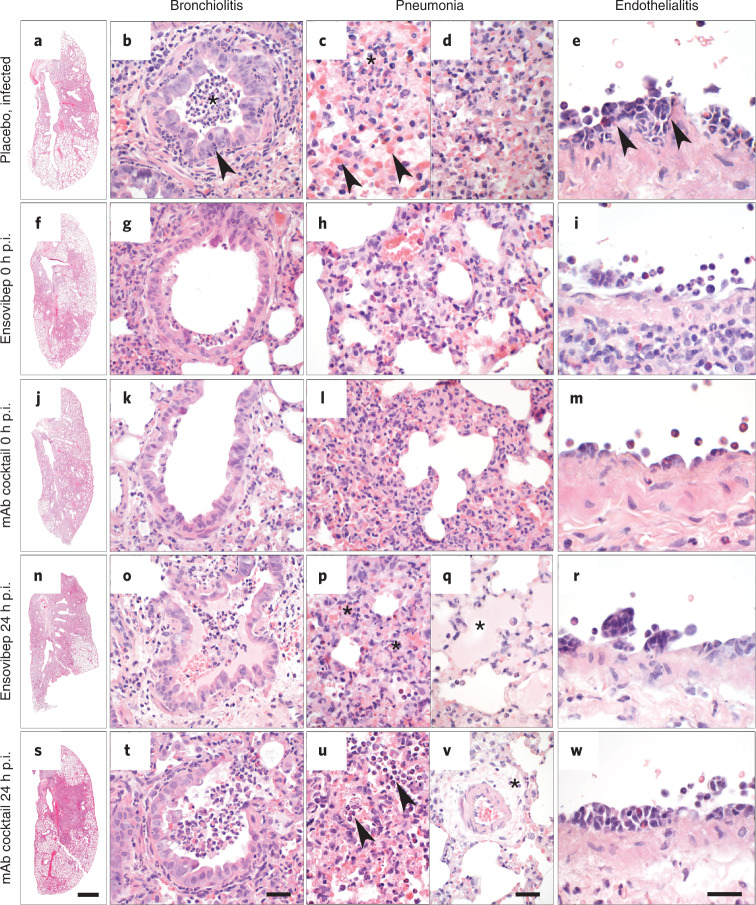
Lung histopathology of Roborovski dwarf hamsters at 2 days or 3 days p.i. **a**–**e**, Lungs of untreated hamsters at day 2/3 p.i. developed marked inflammation. **a**, Whole slide scan revealing consolidation of approximately 60% of the left lung. **b**, Untreated hamsters had moderate necro-suppurative and hyperplastic bronchiolitis with intraluminal accumulation of neutrophils and cellular debris (asterisk) as well as neutrophils transmigrating through the bronchial epithelium into the lumen (arrowhead). The lung parenchyma presented with a patchy distribution of acute necrosis (**c**, asterisk) with microvascular thrombosis (arrowheads) or with areas of dense infiltration by macrophages and neutrophils (**d**). **e**, Pulmonary blood vessels had mild to moderate endothelialitis. **f**–**i**, Lungs of hamsters treated with ensovibep on the day of infection developed moderately less consolidation of their lungs (**f**). **g**, Bronchiolitis was milder with less inflammatory cell infiltrate compared to the untreated group. Neutrophils were mostly absent. **h**, Alveolar walls were only moderately expanded by neutrophils and macrophages with less alveolar edema compared to untreated hamsters. **i**, Endothelialitis was virtually absent with marginating neutrophils as only immune cells interacting with the vascular lining. **j**–**m**, Hamsters treated with the antibody cocktail at the day of infection developed lesions that were similar to those as described for the ensovibep-treated group. **n**–**w**, In contrast, lungs of hamsters treated at 1 day p.i. had lesions similar to the untreated hamsters at that time, regardless of their treatments. **o**,**t**, Both treatment groups developed moderate bronchiolitis similarly to the untreated group. **p**,**u**, Interstitial (asterisks) and alveolar (arrowheads) infiltration with neutrophils and macrophages with variable necrosis of alveolar epithelial cells. Additional lesions in both treatment groups included moderate to marked alveolar edema (**q**, asterisk), here shown for the ensovibep group, and moderate interstitial edema (**v**, asterisk), here shown for the antibody group. **r**,**w**, Both treatment groups developed moderate endothelialitis with monomorphonuclear infiltrates underneath detached endothelial cells, similarly to the untreated group. Scale bars: **a**, **f**, **j**, **n**, **s**, 1 mm; **b**, **g**, **k**, **o**, **t**, 50 µm; **c**, **d**, **h**, **l**, **p**, **q**, **u**, **v**, **e**, **i**, **m**, **r**, **w**, 20 µm.

**Extended Data Fig. 2:**
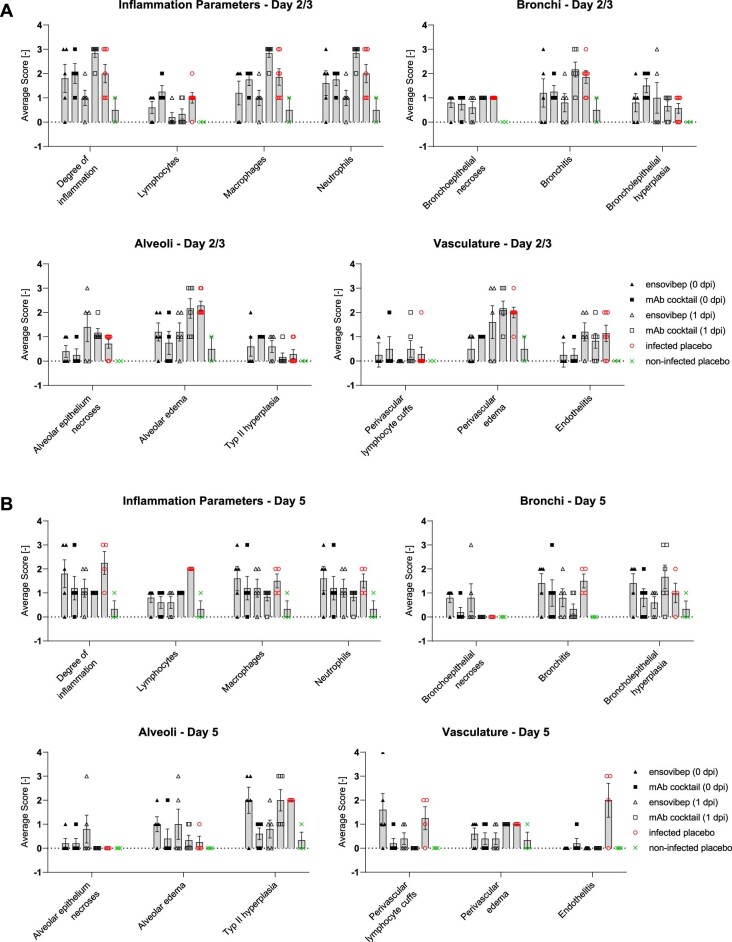
Scoring of Histopathologic Lesions. Histopathologic lesions were scored semi-quantitatively and scores plotted as graphs for histologic signs of general inflammation and histologic parameters of bronchiolar, alveolar and vascular lesions at day 2/3 p.i (**a**) or day 5 p.i. (**b**). Data are represented as mean values +/- SEM. Number of animals analyzed per treatment group at days 2/3; day 5 p.i.: Ensovibep 0 h: n = 5; 5 / mAb cocktail 0 h: n = 4; 5 / Ensovibep 24 h: n = 5; 5 / mAb cocktail 24 h: n = 6; 6 / Placebo, infected: n = 8; 4 Placebo, non-infected: n = 3; 3. The rational for excluding animals is the identification of animals with low drug exposure, likely due to a failure of i.p. injections.

**Extended Data Fig. 3:**
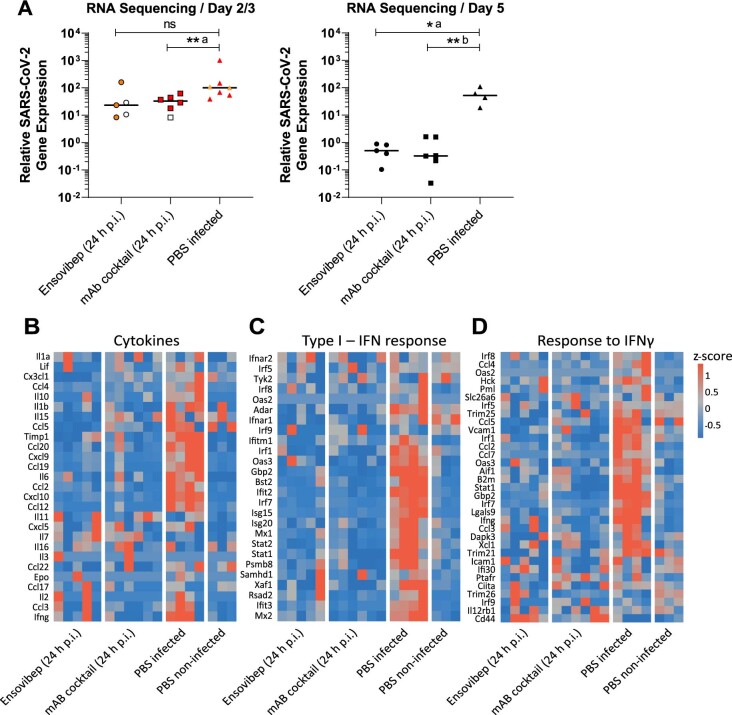
Transcriptome Analysis of Animals Treated 24 h p.i. in Comparison to Placebo Group. Extended Data Fig. 3: **a**) SARS-CoV-2 relExp: Comparison of the relative expression of total canonical junction-spanning viral mRNA transcripts, compared to the total genomic transcripts, among dwarf hamsters infected with SARS-CoV-2 after 2/3 and 5 days, and receiving therapeutically 24 h p.i. ensovibep, mAb cocktail or a PBS solution. Values are shown in log10 scale for both time-points. Red symbols: animals taken out of the study at day 2 due to severe clinical symptoms. Orange symbols: animals taken out of the study at day 3 due to severe clinical symptoms. Data is represented by the median and values for individual animals. Number of animals analyzed per treatment group at days 2/3; day 5 p.i.: Ensovibep 24 h: n = 5; 5 / mAb cocktail 24 h: n = 6; 6 / Placebo, infected: n = 7; 4. The rational for excluding animals in the Ensovibep 24 h group is the identification of animals with low drug exposure, likely due to a failure of i.p. injections. In addition, one sample in the PBS group did not yield enough RNA and was excluded. Statistics: two-tailed Mann-Whitney Test. P-values: ns = P > 0.05; *a: 0.0159; **a: 0.0082; **b: 0.0095. **b-d**) Heatmaps of differently expressed genes in the lung of dwarf hamsters after infection with SARS-CoV-2 and treatment with ensovibep or a mAb cocktail. Genes related to cytokine-mediated signaling pathway (**b**), type I IFN signaling (**c**), cellular-response to IFNy and pro-inflammatory cytokines (**d**) were selected, as previously described by Winkler et al. (2020) (10.1038/s41590-020-0778-2). Columns represent samples and rows genes. Shown are z-scores of DESeq2-normalized data and color scale ranges from blue (10 % lower quantile) to red (10 % upper quantile) of the selected genes.

We determined 10 mg kg^−1^ as optimal dose for ensovibep treatment and used it for comparison to REGN10933 and REGN10987 against the SARS-CoV-2 Alpha variant. We chose treatment timepoints at the time of infection to mimic clinical post-exposure prophylaxis and at 24 hours p.i. to mimic treatment at the onset of clinical symptoms (Fig. 4a). For post-exposure prophylaxis, we confirmed protection from disease for both treatments with notable reduction of virus loads, particularly in the lungs of treated animals compared to placebo-treated controls at all timepoints (Fig. 5a). No obvious differences were observed between the two agents; however, a slight trend toward lower viral load in the antibody cocktail group was observed at 5 days p.i. (Fig. 5a). By contrast, we observed differences between the groups treated 24 hours p.i. (Fig. 4b–d). Animals treated with ensovibep presented with improved condition at 2 days p.i., with 0/10 of the animals reaching a defined humane endpoint, whereas 5/12 animals in the mAb cocktail group and 5/12 animals in the placebo group had to be euthanized (Fig. 5b). Nevertheless, 3/10 hamsters in the ensovipeb group and an additional three hamsters in the placebo group reached defined endpoints at day 3 p.i., whereas no further animals in the mAb cocktail group developed severe illness (Fig. 4b). After the 24-hour p.i. treatment, no substantial differences in average body weights or temperatures were observed in any of the treatment groups (Fig. 4c,d and Supplementary Fig. 12). This is likely a result of the early termination of severely sick animals. However, examination of these parameters on day 2 p.i. revealed significantly higher body weights in both treatment groups compared to the placebo group and a trend toward higher body temperatures in the ensovibep group compared to the other groups (Fig. 4c,d). As body temperature decrease is a very sensitive parameter of disease in this species^36^, this, in particular, is reflective of the better condition of animals in the 24-hour p.i. ensovibep-treated group. Both treatments resulted in marked reduction of virus loads compared to the placebo group (Fig. 5a,b), with no appreciable difference between mAb and ensovibep treatment. This result was more pronounced at the level of replicating virus, indicating efficient neutralization of cell-free virus in both treatment groups (Fig. 5b). Although the histological outcome of infection was similar between both treatment groups (Fig. 6), semi-quantitative assessment of SARS-CoV-2-induced lesions revealed consistently higher scores for the mAb-treated group compared to ensovibep (Extended Data Fig. 2). Scores for inflammation in the mAb-treated group were, on average, exceeding the scores obtained for the placebo group. On a transcriptional level, gene sets representing pro-inflammatory cytokines or genes involved in cytokine-mediated signaling were overall reduced after treatment of hamsters with either ensovibep or the mAb cocktail (Supplementary Note and Extended Data Fig. 3). However, the translational significance of these findings to the clinic has not been completely defined.

We performed a pharmacokinetic analysis for both treatments and found similar exposures in non-infected hamsters after intraperitoneal (i.p.) administration. Ensovibep achieved a higher maximal serum concentration and a shorter systemic half-life compared to the mAb cocktail (Supplementary Fig. 13). To account for failed i.p. injections, we screened terminal serum samples and removed data of animals lacking proper exposure from all analyses (Supplementary Table 4).

## Discussion

The structural analysis of ensovibep provides insights to the mode of action enabling low picomolar neutralizing activity observed against the currently most frequent SARS-CoV-2 mutations and variants. We measured the activity of ensovibep on a panel of single spike protein mutations that have been shown to be of concern because they may be associated with increased transmissibility or disease severity, or they affect neutralization of some monoclonal or polyclonal antibodies^1,44,45^. Of mutations tested, F486 and Y489 substitutions caused the greatest potency reduction when compared to wild-type or reference virus. Mutations at these positions were also noted in the viral passaging study. F486L was identified from sequencing of mutations allowing escape from inhibition by the monovalent DARPin R2, incorporated in ensovibep (Fig. 3b). F486 and Y489 are key binding residues for the interaction of ensovibep with the RBD. However, F486 and Y489 are also important for viral infection, being residues involved in binding of the spike complex to ACE2 and lower the infectivity of the virus^21,25,46,47^ (Supplementary Fig. 9).

The reduced neutralization potency observed for ensovibep and its single DARPin domains against viruses bearing the N234Q mutation outside the RBD might be explained by the effect of this mutation on the RBD conformational dynamics. An in silico simulation study showed that this conserved glycosylation site, together with N165, might be involved in the stabilization of the RBD up-conformation^42^. Because the RBD epitope for binding of ensovibep is exposed only in the up-conformation, a mutation in one of these glycosylation sites might affect binding equilibrium, as suggested in our neutralization assays. The N234Q mutation might affect all protein-binding scaffolds that are binding exclusively to the up-conformation of the RBD and reduces the affinity of the spike protein for the human ACE2 receptor, as demonstrated previously^42^.

Some mutations, not predicted to be key interaction residues for ensovibep (for example, E484K or Q493K), led to a reduction in potency for one or several of the RBD-binding monovalent DARPin domains, whereas the trispecific ensovibep molecule maintained full neutralization capacity. This demonstrates that the trispecific DARPin design of ensovibep, cooperatively binding with three distinct paratopes (Supplementary Fig. 2), results in high neutralizing potency, even if binding affinity for an individual DARPin domain is decreased (Fig. 2c and Supplementary Fig. 8). This cooperative binding of multiple paratopes is a hallmark of the trispecific nature of ensovibep, differentiating the molecule from mAb candidates by affording full neutralization of even the highly mutated SARS-CoV-2 variants, such as Omicron BA.1 and BA.2.

Ensobibep’s high level of protection against viral escape mutations was also demonstrated in a viral passaging experiment. The single mAbs and the monovalent DARPin R2 were overcome by escape mutants, whereas ensovibep maintained potency similar to a clinically validated mAb cocktail.

Translatability of the observed in vitro activity of ensovibep against SARS-CoV-2 was evaluated in a COVID-19 model using highly susceptible Roborovski dwarf hamsters. Using this in vivo model, we confirmed the therapeutic benefit of ensovibep, with outcomes similar to a clinically validated antibody cocktail. In a late intervention scenario, ensovibep treatment yielded prolonged survival of animals and reduced inflammation of the lungs when compared to mAb-treated and placebo-treated animals. In a pharmacokinetic profile, ensovibep showed a higher maximal serum uptake from the peritoneum compared to an antibody cocktail (Supplementary Fig. 13), which might be contributing to differentiated outcomes. Concomitantly, reduction of viral load was similar in the ensovibep-treated and mAb-treated groups, and both differed significantly from the control group, as determined by virus genomic RNA (gRNA), replicating virus and virus transcript levels. Potential mechanistic differences affecting virus particle clearance, such as phagocytosis, which is possibly related to Fc effector function, could not be assessed from the current study.

In conclusion, ensovibep has shown highly potent neutralization against the most frequent SARS-CoV-2 variants to date due to its cooperative and complementary binding to a highly conserved epitope region on the spike RBD. In vitro and in vivo single-agent efficacies closely match the performance of a clinically validated mAb cocktail. Ultimately, ensovibep demonstrates the potential to prevent severe disease and reduce viral load in a highly susceptible in vivo model under different treatment scenarios. The clinical translatability of these results is currently being investigated in the treatment of ambulatory patients with COVID-19. If successful, *Escherichia coli-*based manufacturing will allow rapid and large-scale production for global access to this new class of therapeutics to add to the COVID-19 armamentarium.

## Methods

### Generation of His-tagged monovalent RBD binders and ensovibep

DARPin constructs selected and cloned as described in the Supplementary Methods were transformed in *E. coli* BL21 cells (Merck), plated on LB agar (containing 1% glucose and 50 μg ml^−1^ of ampicillin) (Merck) and then incubated overnight at 37 °C. A single colony was picked into TB medium (containing 1% glucose and 50 μg ml^−1^ of ampicillin) and incubated overnight at 37 °C, shaking at 230 r.p.m. Fresh TB medium (containing 50 μg ml^−1^ of ampicillin) was inoculated with 1:20 of overnight culture and incubated at 37 °C at 230 r.p.m. At OD_600_ = 1.1, the culture was induced by the addition of IPTG (0.5 mM final concentration) and incubated further for 5 hours at 37 °C and 230 r.p.m. Harvest was done by centrifugation (10 minutes, 5,000*g*). After cell disruption by sonication, primary recovery was done by heat treatment for 30 minutes at 62.5 °C and subsequent centrifugation (15 minutes, 12,000*g*). Then, 20 mM imidazole and 1% Triton X-100 were added to the supernatant, and the 0.22-µm-filtered supernatant was further purified by immobilized metal affinity chromatography (IMAC) (HisTrap FF crude, Cytiva) using the N-terminal His-tag and including a wash step with 1% Triton X-100 and a step elution with 250 mM imidazole (Merck). In a subsequent step, the elution fraction of the IMAC step was applied on a size exclusion chromatography (Superdex 200, Cytiva), and fractions of interest were pooled and concentrated. Finally, the concentrated sample was filtered through a 0.22-µm Mustang E filter (Pall) for endotoxin removal and sterile filtration and quality controlled.

### cryo-EM

Next, 4 μl of purified S-ectodomain (9 μM) was mixed with 1 μl of 50 μM DARPin R2 and incubated for 15 seconds at 21 °C. Then, 3 μl of sample was dispensed on Quantifoil R1.2/1.3 200 mesh grids that had been freshly glow-discharged for 30 seconds at 20 mA. Grids were blotted using blot force +2 for 5 seconds using Whatman No. 1 filter paper and immediately plunge-frozen into liquid ethane cooled by liquid nitrogen using a Vitrobot Mark IV plunger (Thermo Fisher Scientific) equilibrated to ~95% relative humidity at 4 °C. Movies were collected using Glacios Cryo-TEM (Thermo Fisher Scientific) operating at 200 keV and equipped with a Falcon 4 Direct Electron Detector (Thermo Fisher Scientific). For additional analysis of DARPin R2, 4 μl of purified S-ectodomain (18 μM) was mixed with 1 μl of 100 μM DARPin and incubated for 60 seconds at 21 °C. Grids were prepared as described above, and movies were collected using a Titan Krios Cryo-TEM (Thermo Fisher Scientific) operating at 300 keV and equipped with a Falcon 4 Direct Electron Detector. All cryo-EM data were acquired using EPU 2 software (Thermo Fisher Scientific) with a 30-degree stage tilt to account for preferred orientation of the samples. Movies were collected in electron counting mode at 92,000× (Glacios) or 75,000× (Titan Krios), corresponding to a pixel size of 1.1 Å per pixel or 1.045 Å per pixel over a defocus range of −1.25 μm to −2.5 μm.

### Image processing

Movies were manually inspected and then imported into Relion (version 3.1.1)^48^. Drift and gain correction were performed with MotionCor2 (version 1.3.0)^49^, and GCTF (version 1.06)^50^ was used to estimate the contrast transfer function. Particles were picked using the Laplacian-of-Gaussian (LoG) algorithm, and then Fourier-binned (2 × 2) particles were extracted in a 160-pixel box. Extracted particles were subjected to two rounds of two-dimensional (2D) classification, ignoring CTFs until the first peak. Using the ‘molmap’ command in UCSF Chimera^51^, a SARS-CoV-2 spike structure (Protein Data Bank (PDB) ID: 6VSB)^52^ was used to generate a 50-Å-resolution starting model for 3D classification. Particles selected from 2D classification were subject to a single round of 3D classification (with C1 symmetry). Particles belonging to the best classes were re-extracted unbinned in a 320-pixel box, 3D auto-refined (with C1 or C3 symmetry) and post-processed. Iterative rounds of per-particle defocus estimation, 3D auto-refinement and post-processing were used to account for the 30-degree stage tilt used during data collection. When CTF refinement did not yield any further improvement in resolution, Relion’s Bayesian polishing procedure was performed on the particle stacks, with all movie frames included, followed by 3D auto-refinement and post-processing. Subsequently, additional rounds of per-particle defocus estimation, 3D auto-refinement and post-processing were performed on the polished particles until no further improvement in resolution or map quality was observed. The nominal resolution for each map was determined according to the ‘gold standard’ Fourier shell correlation (FSC) criterion (FSC = 0.143), and local resolution estimations were performed using Relion. Map sharpening was performed using DeepEMhancer (version 0.13)^53^ as implemented in COSMIC2 (ref. ^54^). To improve the quality of the DARPin R2 density in the fully open spike reconstruction, a focused 3D classification approach was employed. In brief, each particle contributing to the final C3-symmetry-imposed reconstruction was assigned three orientations corresponding to its symmetry-related views using the ‘relion_particle_symmetry_expand’ tool. A soft mask was placed over the map to isolate the DARPin R2-bound RBD, and the symmetry-expanded particles were subjected to masked 3D classification without alignment using a regularization parameter (‘T’ number) of 20. Particles corresponding to the 3D class with the best resolved DARPin density were re-extracted in a 200-pixel box and centered on the mask used for focused classification. In conjunction with this, the signal for the protein outside the masked was subtracted. The re-extracted particles were then 3D auto-refined (with C1 symmetry) using local angular searches (1.8 degrees) and sharpened using DeepEMhancer^53^. Three copies of the locally refined map were aligned to the globally refined map using the UCSF Chimera ‘fit in map’ tool and resampled using the ‘vop resample’ command. Finally, a composite map was generated using the ‘vop add’ command. An overview of the image processing workflows is shown in Supplementary Fig. 4a.

### Molecular modeling of monovalent and multivalent DARPin molecules

Homology models of DARPin R1, R2 and R3 were generated with Rosetta version 3.11(2019.35.60890)^55–57^. The consensus-designed ankyrin repeat domain (PDB ID: 2XEE) was used as template. Mutations were introduced with RosettaRemodel with fixed backbone, and the structure was refined with RosettaRelax. Forty refined structures were clustered using RosettaCluster with 0.3-Å radius, and the lowest-energy model from the largest cluster served as the final model. The UCSF Chimera ‘fit in map’ tool was used to fit the DARPin R2 model into the locally refined EM map. This fitted model of DARPin R2, together with the RBD domain (PDB ID: 6M0J), was further refined with Rosetta. The structure was pre-relaxed for docking and served as input for local, high-resolution docking with RosettaDock with fixed backbone. Five hundred models were generated and clustered with 1-Å radius (RosettaCluster). The two largest clusters were inspected, and the lowest-energy model from the more conserved group (that is, with lower rigid body perturbation from the input structure) was taken further for additional all-atom refinement with RosettaRelax, with protocol optimized for interfaces (InterfaceRelax2019). Fifty models were generated, and the lowest-scoring model was selected. This model was used to describe the interactions between DARPin R2 and the RBD. The PDB file with the coordinates of the trimer of DARPin R2:RBD was used as an input structure for the conceptual modeling of ensovibep bound to the spike ectodomain as shown in Fig. 1g. The linkers were generated using Rosetta modeling tools. Figures were generated with UCSF Chimera (version 1.15.0)^51^, UCSF ChimeraX (version 1.2.5)^58^ and PyMOL (version 2.0, Schrödinger).

### Generation of mAbs

All used mAbs were custom produced at evitria AG based on publicly available sequences. Production numbers: REGN10933; 902071.2 / LY-CoV555; 901968.1 / LY-CoV016; 902385.1 / REGN10987; 902709.1 / AZD8895; 905290.1 / AZD1061; 905290.2 / S309; 905290.3 / BRII-196; 905342.1 / BRII-198; 905342.2. All mAbs passed quality control (by SDS–PAGE, size exclusion chromatography and endotoxin measurement) before use in assays.

### VSV–SARS-CoV-2 pseudotype mutation vector generation

Plasmid pCAGGS containing the Wuhan-hu-1 spike protein of SARS-CoV-2 (ref. ^7^) was used as a reference and as template for generation of single and multiple spike protein mutants. Forward and reverse complementary primers encoding the mutation were synthesized by Microsynth. High-fidelity Phusion polymerase (New England Biolabs) was used for all DNA amplification.

Mutations of the spike protein were generated via site-directed mutagenesis, with DNA fragments generated via polymerase chain reaction (PCR) (Phusion polymerase, New England Biolabs) with a generic forward primer upstream of the spike open reading frame (ORF) (pCAGGS-5; GGTTCGGCTTCTGGCGTGTGACC) and with a mutation-specific reverse primer or a generic reverse primer (rbglobpA-R; CCCATATGTCCTTCCGAGTG) downstream the ORF. Fragments were used as input for an assembly PCR. For multi-mutation spike proteins, a complementary pair of primers was designed. The full-length mutated spike ORF was inserted into the pCAGGS vector backbone. Sequence was verified by Microsynth.

### VSV–SARS-CoV-2 pseudotype neutralization assay

The pseudotype viral system was based on the recombinant VSV*ΔG-Luc vector in which the glycoprotein gene (G) had been deleted and replaced with genes encoding green fluorescent protein and luciferase^59^. Pseudoviruses were generated as reported previously^60,61^. For the neutralization assay, an initial dilution of the compounds was followed by three-fold dilutions in quadruplicates in DMEM-2% (vol/vol) FCS supplemented with 20 μM HSA (CSL Behring). The mixture was mixed with an equal volume of DMEM-2% FCS containing 250 infectious units per well of SARS-CoV-2 pseudoviruses and incubated for 90 minutes at 37 °C. The mix was inoculated onto Vero E6 cells (supplied by the American Type Culture Collection (ATCC), CRL-1586) in a clear-bottom, white-walled 96-well plate during 90 minutes at 37 °C. The inoculum was removed and fresh medium added, and cells were further incubated at 37 °C for 16 hours. Cell were lysed according to the ONE-Glo luciferase assay system (Promega), and light emission was recorded using a Berthold TriStar LB941 luminometer. The raw data (relative luminescence unit (RLU) values) were exported to GraphPad Prism version 8.4.3, and the % neutralization values were normalized to the untreated PsV signal. IC_50_ values with 95% confidence intervals were estimated by the model of non-linear regression fit with settings for log (inhibitor) versus normalized response curves. Data points are plotted by the mean ± s.e.m. of quadruplicate data.

### SARS-CoV-2 lentivirus-based pseudovirus neutralization assay

The neutralizing activity of the compounds was measured using lentiviral particles pseudotyped with spike proteins of SARS-COV-2 variants, as previously described^62^. In brief, pseudoviruses bearing the spike proteins and carrying a firefly luciferase^63^ reporter gene were produced in 293T cells (ATCC, CRL-3216) by co-transfection of pCMVΔR8.2, pHR′CMVLuc and pCDNA3.1-spike variants. Plasmids encoding human codon-optimized spike genes with the desired mutations were purchased (GenScript). Supernatants containing pseudoviruses were collected 48 hours after transfection, filtered and stored at −80 °C. Pseudovirus titers were measured by infecting 293T-ACE2.TMPRSS2s cells^62^ for 48 hours before measuring luciferase activity (luciferase assay reagent, Promega). For neutralization assays, pseudoviruses with titers of approximately 10^6^ RLU ml^−1^ were incubated with serially diluted compounds for 2 hours at 37 °C before adding the pseudovirus and antibody mixtures (100 μl) onto 96-well plates pre-seeded 1 day earlier with 3.0 × 10^4^ 293T-ACE2.TMPRSS2s cells per well. Pseudovirus infection was scored 48 hours later by measuring luciferase activity (SpectraMax Plate Reader, Molecular Devices). The concentration causing a 50% reduction of RLU compared to control (ID_50_) was reported as the neutralizing antibody titer. Titers were calculated using a non-linear regression curve fit (GraphPad Prism). The mean titer from at least two independent experiments with intra-assay duplicates was reported as the final titer. This work was performed independently by investigators at the US Food and Drug Administration, Center for Biologics Evaluation and Research, as part of the Therapeutics Research Team for the US government COVID-19 response efforts.

### SARS-CoV-2 lentivirus-based pseudovirus neutralization assay (setup 2)

Some neutralizing activity was measured with a second lentiviral pseudotype assay setup. Data from this setup represent single runs. This work was performed independently by investigators at Monogram Biosciences for the US government COVID-19 response efforts. The assay setup is described in detail by Huang et al.^64^.

### Cells and pathogenic virus

Vero E6 cells (kindly provided by Volker Thiel, University of Bern) were passaged in MEM containing 10% FCS and supplements (2 mM L-glutamine, 1% non-essential amino acids, 100 U ml^−1^ of penicillin, 100 μg ml^−1^ of streptomycin and 0.06% sodium bicarbonate, all from Bioswisstec) at 37 °C, >85% humidity and 5% CO_2_. Vero E6/TMPRSS2 cells^65,66^ obtained from the Centre For AIDS Reagents (National Institute for Biological Standards and Control) were passaged in DMEM containing 10% FCS and supplements at 37 °C, >85% humidity and 5% CO_2_.

SARS-CoV-2 (2019-nCoV/IDF0372/2020) was propagated in Vero E6 cells in MEM containing 2% FCS and supplements (2%-FCS-MEM) at 37 °C, >85% humidity and 5% CO_2_. SARS-CoV-2 variants (B.1.1.7, B.1.351 and P.1) were provided by the University Hospital of Geneva, Laboratory of Virology^33^, and propagated in Vero E6/TMPRSS2 cells in DMEM containing 2% FCS and supplements (2%-FCS-DMEM) at 37 °C, >85% humidity and 5% CO_2_. Viral titer was determined by standard plaque assay, by incubating ten-fold serial dilutions of the virus for 1 hour at 37 °C on a confluent 24-well plate with Vero E6 cells. Then, inoculum was removed, and 1 ml of overlay medium (20 ml DMEM, 5 ml FCS, 100 U ml^−1^ of penicillin, 100 μg ml^−1^ of streptomycin and 25 ml of Avicel rc581) was added. After 3 days of incubation at 37 °C, the overlay was removed, and the plates were stained with crystal violet.

### Viral passaging experiment with authentic SARS-CoV-2

Virus escape studies were adapted from Baum et al.^1^. In brief, 1:5 serial dilutions of the compounds from 100 μg ml^−1^ to 0.032 μg ml^−1^ were prepared in MEM 2% FCS, supplements and 10 μM HSA (CSL Behring, 2%-FCS-MEM+HSA). Next, 500 µl of virus suspension containing 1.5 × 10^6^ plaque-forming units (PFU) of SARS-CoV-2 in 2%-FCS-MEM+HSA was mixed with 500 μl of serially diluted DARPin molecules or mAbs and subsequently incubated for 1 hour at 37 °C. The mixtures were then transferred to confluent Vero E6 cells in 12-well plates and incubated for 4 days at 37 °C, >85% humidity and 5% CO_2_. Each culture well was assessed for CPE by microscopy. The supernatant was removed from wells with the highest DARPin or antibody concentrations showing substantial CPE (≥20%) and used for total RNA extraction and further passaging. For the next passage, the remaining 900 μl of supernatant was diluted in 4 ml in 2%-FCS-MEM+HSA, and 500 μl of this dilution was mixed with serial dilutions of the compounds and transferred to 12-well plates with fresh Vero E6 cells as described above. Cell culture wells were assessed for CPE again after 4 days, and the supernatant of wells with highest DARPin or antibody concentrations with evident viral replication (CPE) were harvested and used for additional passages.

### Deep sequencing of viral passages

RNA of the cell culture supernatant was extracted using the RNeasy Universal Plus Kit (Qiagen), according to the manufacturer’s protocol. Next, 10.5 µl of the extract was reverse transcribed using SuperScript VILO (Thermo Fisher Scientific), following the manufacturer’s instructions. Barcoded libraries were prepared on the Ion Chef Instrument (Thermo Fisher Scientific) using the Ion AmpliSeq SARS-CoV-2 Research Panel (Thermo Fisher Scientific). Then, 8–16 barcoded samples were pooled and loaded on one Ion 530 chip using the Ion Chef Instrument and sequenced on the Ion S5 System with 550 flows. The resulting BAM files were converted to FASTQ format using SAMtools 1.10 (ref. ^67^) and subjected to adapter and quality trimming using Trimmomatic 0.39 (ref. ^68^) (options: ILLUMINACLIP:adapters.fasta:2:30.10, LEADING: 3, TRAILING: 3, SIDINGWINDOW: 4:15, MINLEN: 36). Reads were aligned to the SARS-CoV-2 reference genome (NC_045512.2) using bwa 0.7.17 (ref. ^69^), and variants were determined using LoFreq version 2.1.5 (ref. ^70^). Variants were filtered for a minimal depth of 400× and a minimal allele frequency of 3% using bcftools 1.10 (ref. ^67^). Functional annotation of the variants was performed using SNPEff 5.0 (ref. ^71^). Variants were visualized in R 3.6.1 using ComplexHeatmap 2.2 (ref. ^72^).

### Virus neutralization of authentic wild-type and variants of SARS-CoV-2

Virus neutralization was determined using 100 TCID_50_ SARS-CoV-2 variants from lineage B.1.1.7 and P.1 or the wild-type French isolate (with the following differences to the Wuhan wild-type: V367F and E990A) in a cell viability assay. DARPin molecules were serially diluted 1:4 from 40 nM to 2.4 pM (in triplicates) in 100 μl of cell culture medium (2%-FCS-DMEM) supplemented with 10 μM HSA in 96-well plates, mixed with 100 TCID_50_ SARS-CoV-2 in 100 μl of 2%-FCS-MEM with HSA and incubated for 1 hour at 37 °C. The mixtures were transferred onto confluent Vero E6/TMPRSS2 cells. The controls consisted of cells exposed to virus suspension only, to determine maximal CPE, and of cells incubated with medium only, to determine baseline cell viability. The plates were incubated for 3 days at 37 °C, >85% humidity and 5% CO_2_. Cell viability was determined by removing 100 μl of supernatant from all wells and adding 100 μl of CellTiter-Glo reagent (CellTiter-Glo Luminescent Cell Viability Assay, Promega). Luminescence was measured using a GloMax instrument (Promega).

### Roborovski dwarf hamster model for the assessment of antiviral protection

#### Cells and viruses

For in vivo experiments, SARS-CoV-2 isolates BetaCoV/Germany/BavPat1/2020 and BetaCoV/Germany/ChVir21652/2020 (B.1.1.7) were grown on Vero E6 cells and whole-genome sequenced before infection experiments to confirm genetic integrity. All virus stocks were titrated on Vero E6 cells before infection.

#### Animals and infection

A total of 120 female (67) and male (53) Roborovski dwarf hamsters (*Phodopus roborovskii*) obtained via the German pet trade were used for infection experiments. Animals were housed in groups of 3–6 animals of the same sex in individually ventilated GR900 cages (Tecniplast) and provided with food and water ad libitum and bountiful enrichment (Carfil). Infection was performed by intranasal administration of 1 × 10^5^ PFU of SARS-CoV-2 in 20 µl of cell culture medium under general anesthesia^36^. All animal procedures were performed in accordance with relevant institutional and legal regulations and approved by the responsible state authority: Landesamt für Gesundheit und Soziales, Berlin, Germany, permit number G 0086/20.

#### Treatment

DARPin molecules and mAbs were administered intraperitoneally in sterile PBS. The final drug concentration was adjusted based on the desired dose and respective animal weight to a 100-µl injection volume. All animals in this study were treated once at the indicated timepoint, 0 hours, 6 hours or 24 hours p.i.

#### Experimental groups

From a total of 120 Roborovski dwarf hamsters, 54 were used to determine dose and time dependency of treatment success. Six animals (four female and two male) per group were infected with 1 × 10^5^ PFU of SARS-CoV-2 wild-type (BetaCoV/Germany/BavPat1/2020) and treated with 3 mg kg^−1^, 10 mg kg^−1^ or 20 mg kg^−1^ of ensovibep at the time of infection, with 1 mg kg^−1^ or 20 mg kg^−1^ 6 hours p.i. or with 10 mg kg^−1^ 24 hours p.i. A placebo (PBS) treatment group with six animals (four female and two male) was also included in each of three studies performed for this purpose. Results of these experiments are summarized in Supplementary Fig. 11.

To compare efficacy of ensovibep and mAb cocktail treatment, 60 animals were infected with 1 × 10^5^ PFU of SARS-CoV-2 variant B.1.1.7 (BetaCoV/Germany/ChVir21652/2020). Subjects were divided into groups of 12 animals (six female and six male) and treated with 10 mg kg^−1^ of ensovibep, 10 mg kg^−1^ of Regeneron mAb cocktail or placebo (PBS) at the time of infection or with 10 mg kg^−1^ of ensovibep or 10 mg kg^−1^ of Regeneron mAb cocktail 24 hours p.i. An additional six (three female and three male) animals served as a non-infected control group. Results of this experiment are presented in Figs. 4–6.

In all in vivo infection experiments performed in this study, half of each respective group was scheduled for take-out at 3 days p.i.; the other half was to be terminated at 5 days p.i. In some of the experiments, several animals had to be terminated at timepoints other than these for humane reasons. Defined humane endpoints included body temperature <33 °C, body weight loss >15% together with signs of respiratory distress, body weight loss >20% or a combination of these factors. Animals were monitored at least twice a day to prevent any prolonged suffering.

#### Virological analysis

RNA was extracted from throat swabs and lung tissue using the innuPREP Virus DNA/RNA Kit (Analytic Jena). Viral RNA was quantified using a one-step RT–qPCR reaction with the NEB Luna Universal Probe One-Step RT–qPCR (New England Biolabs) and the 2019-nCoV RT–qPCR primers and probe (E_Sarbeco)^73^ on a StepOnePlus Real-Time PCR System (Thermo Fisher Scientific), as previously described^36^. To obtain virus titers, duplicate ten-fold serial dilutions of lung tissue homogenates were made and incubated on Vero E6 monolayers for 2 hours at 37 °C. Cells were washed and overlaid with semi-solid cell culture medium containing 1.5% microcrystalline cellulose (Avicel) and incubated for 48 hours at 37 °C. Plates were then fixed with 4% formalin and stained with 0.75% crystal violet for plaque counting.

#### Histology

For histopathology, the left lung lobe was carefully removed, immersion-fixed in formalin, pH 7.0, for 48 hours, embedded in paraffin and cut in 2-μm sections. Slides were stained with hematoxylin and eosin (H&E) after de-waxing in xylene and rehydration in decreasing ethanol concentrations. Lung sections were microscopically evaluated in a blinded fashion by a board-certified veterinary pathologist to assess the character, distribution and severity of pathologic lesions using lung-specific inflammation scoring parameters as previously described for other lung infection models. Three different scores were used that included the following parameters: (1) lung inflammation score including severity of (i) interstitial pneumonia, (ii) bronchiolitis, (iii) necrosis of bronchial and alveolar epithelial cells and (iv) hyperplasia of alveolar epithelial type II cells as well as (v) hyperplasia of bronchial epithelial cells; (2) immune cell infiltration score taking into account the presence of (i) neutrophils, (ii) macrophages and (iii) lymphocytes in the lungs as well as (iv) perivascular lymphocytic cuffing; and (3) edema score including (i) alveolar edema and (ii) perivascular edema. H&E-stained slides were analyzed, and images were taken using an Olympus BX41 microscope with a DP80 Microscope Digital Camera and cellSens Imaging Software, version 1.18 (Olympus). For the display of overviews of whole lung lobe sections, slides were automatically digitized using the Aperio CS2 slide scanner (Leica Biosystems Imaging), and image files were generated using Image Scope Software (Leica Biosystems Imaging). The percentages of lung tissues affected by inflammation were determined histologically by an experienced board-certified experimental veterinary pathologist (O.K.), as described previously^74^. Lung inflammation scores were determined as absent, (1) mild, (2) moderate or (3) severe and quantified as described previously^74^. Immune cell influx scores and edema scores were rated from absent to (1) mild, (2) moderate or (3) severe.

### Reporting summary

Further information on research design is available in the Nature Research Reporting Summary linked to this article.

## Online content

Any methods, additional references, Nature Research reporting summaries, source data, extended data, supplementary information, acknowledgements, peer review information; details of author contributions and competing interests; and statements of data and code availability are available at 10.1038/s41587-022-01382-3.

## Supplementary information

Supplementary information Supplementary Methods, Supplementary Notes, References for Supplementary Information, Supplementary Figs. 1–14, Supplementary Tables 1–6 and References for Supplementary Figures and Tables

Reporting Summary

## Data Availability

The EM density maps for the SARS-CoV-2 spike ectodomain in complex with monovalent DARPin R2 have been deposited to the Electron Microscopy Data Bank under accession codes EMD-11953, EMD-14810, EMD-14811 and EMD-11954. The atomic coordinates of the SARS-CoV-2 spike ectodomain used to generate the starting model for cryo-EM 3D classification are available from the Protein Data Bank under accession code 6VSB. The atomic coordinates of the template used to generate DARPin R1, R2 and R3 are available from the Protein Data Bank under accession code 2XEE. The monovalent DARPin and multivalent DARPin sequences, as well as pseudo-atomic models derived from molecular docking experiments, are available here, to allow the use of the data for non-commercial purposes. All other data used in the study are included in the manuscript files.

## References

[1] Baum A (2020). Antibody cocktail to SARS-CoV-2 spike protein prevents rapid mutational escape seen with individual antibodies. Science.

[2] Copin R (2021). The monoclonal antibody combination REGEN-COV protects against SARS-CoV-2 mutational escape in preclinical and human studies. Cell.

[3] Ku Z (2021). Molecular determinants and mechanism for antibody cocktail preventing SARS-CoV-2 escape. Nat. Commun..

[4] Binz HK (2017). Design and characterization of MP0250, a tri-specific anti-HGF/anti-VEGF DARPin^®^ drug candidate. MAbs.

[5] Fiedler U (2017). MP0250, a VEGF and HGF neutralizing DARPin^®^ molecule shows high anti-tumor efficacy in mouse xenograft and patient-derived tumor models. Oncotarget.

[6] Binz HK (2004). High-affinity binders selected from designed ankyrin repeat protein libraries. Nat. Biotechnol..

[7] Walser, M. et al. Highly potent anti-SARS-CoV-2 multivalent DARPin therapeutic candidates. Preprint at https://www.biorxiv.org/content/10.1101/2020.08.25.256339v3 (2021).

[8] Stumpp MT, Dawson KM, Binz HK (2020). Beyond antibodies: the DARPin^®^ drug platform. BioDrugs.

[9] Steiner D (2017). Half-life extension using serum albumin-binding DARPin^®^ domains. Protein Eng. Des. Sel..

[10] Zhou P (2020). A pneumonia outbreak associated with a new coronavirus of probable bat origin. Nature.

[11] Shang J (2020). Structural basis of receptor recognition by SARS-CoV-2. Nature.

[12] Tortorici MA, Veesler D (2019). Structural insights into coronavirus entry. Adv. Virus Res..

[13] Letko M, Marzi A, Munster V (2020). Functional assessment of cell entry and receptor usage for SARS-CoV-2 and other lineage B betacoronaviruses. Nat. Microbiol.

[14] Walls AC (2020). Structure, function, and antigenicity of the SARS-CoV-2 spike glycoprotein. Cell.

[15] Walls AC (2016). Cryo-electron microscopy structure of a coronavirus spike glycoprotein trimer. Nature.

[16] Walls AC (2017). Tectonic conformational changes of a coronavirus spike glycoprotein promote membrane fusion. Proc. Natl Acad. Sci. USA.

[17] Hoffmann M (2020). SARS-CoV-2 cell entry depends on ACE2 and TMPRSS2 and is blocked by a clinically proven protease inhibitor. Cell.

[18] Garcia-Beltran WF (2021). Multiple SARS-CoV-2 variants escape neutralization by vaccine-induced humoral immunity. Cell.

[19] Greaney AJ (2021). Complete mapping of mutations to the SARS-CoV-2 spike receptor-binding domain that escape antibody recognition. Cell Host Microbe.

[20] Lusvarghi, S. et al. Key substitutions in the spike protein of SARS-CoV-2 variants can predict resistance to monoclonal antibodies, but other substitutions can modify the effects. *J. Virol.* **96**, e0111021 (2022). 10.1128/JVI.01110-21 PMC8754225 34668774

[21] Starr TN (2020). Deep mutational scanning of SARS-CoV-2 receptor binding domain reveals constraints on folding and ACE2 binding. Cell.

[22] Thomson EC (2021). Circulating SARS-CoV-2 spike N439K variants maintain fitness while evading antibody-mediated immunity. Cell.

[23] Wang, P. et al. Increased resistance of SARS-CoV-2 variants B.1.351 and B.1.1.7 to antibody neutralization. Preprint at https://www.biorxiv.org/content/10.1101/2021.01.25.428137v2 (2021).

[24] Yi C (2020). Key residues of the receptor binding motif in the spike protein of SARS-CoV-2 that interact with ACE2 and neutralizing antibodies. Cell Mol. Immunol..

[25] Liu Z (2021). Identification of SARS-CoV-2 spike mutations that attenuate monoclonal and serum antibody neutralization. Cell Host Microbe.

[26] Zhou D (2021). Evidence of escape of SARS-CoV-2 variant B.1.351 from natural and vaccine-induced sera. Cell.

[27] Gobeil SM-C (2021). Effect of natural mutations of SARS-CoV-2 on spike structure, conformation, and antigenicity. Science.

[28] Tegally, H. et al. Emergence and rapid spread of a new severe acute respiratory syndrome-related coronavirus 2 (SARS-CoV-2) lineage with multiple spike mutations in South Africa. Preprint at https://www.medrxiv.org/content/10.1101/2020.12.21.20248640v1 (2020).

[29] Voloch CM (2021). Genomic Characterization of a Novel SARS-CoV-2 Lineage from Rio de Janeiro, Brazil. J. Virol..

[30] Cele S (2022). Omicron extensively but incompletely escapes Pfizer BNT162b2 neutralization. Nature.

[31] Thomson EC (2021). Circulating SARS-CoV-2 spike N439K variants maintain fitness while evading antibody-mediated immunity. Cell.

[32] Laffeber C, de Koning K, Kanaar R, Lebbink JHG (2021). Experimental evidence for enhanced receptor binding by rapidly spreading SARS-CoV-2 variants. J. Mol. Biol..

[33] Planas D (2021). Reduced sensitivity of SARS-CoV-2 variant Delta to antibody neutralization. Nature.

[34] Copin, R. et al. In vitro and in vivo preclinical studies predict REGEN-COV protection against emergence of viral escape in humans. Preprint at https://www.biorxiv.org/content/10.1101/2021.03.10.434834v1.full (2021).

[35] Cathcart, A. et al. The dual function monoclonal antibodies VIR-7831 and VIR-7832 demonstrate potent in vitro and in vivo activity against SARS-CoV-2. Preprint at https://www.biorxiv.org/content/10.1101/2021.03.09.434607v10 (2021).

[36] Trimpert J (2020). The Roborovski dwarf hamster is a highly susceptible model for a rapid and fatal course of SARS-CoV-2 infection. Cell Rep..

[37] Walls AC (2019). Unexpected receptor functional mimicry elucidates activation of coronavirus fusion. Cell.

[38] Corti D, Purcell LA, Snell G, Veesler D (2021). Tackling COVID-19 with neutralizing monoclonal antibodies. Cell.

[39] Falsey AR (2021). SARS-CoV-2 neutralization with BNT162b2 vaccine dose 3. N. Engl. J. Med..

[40] Hoffmann M (2021). SARS-CoV-2 variants B.1.351 and P.1 escape from neutralizing antibodies. Cell.

[41] Pulliam JRC (2022). Increased risk of SARS-CoV-2 reinfection associated with emergence of Omicron in South Africa. Science.

[42] Casalino L (2020). Beyond shielding: the roles of glycans in the SARS-CoV-2 spike protein. ACS Cent. Sci..

[43] Andreano E (2021). SARS-CoV-2 escape from a highly neutralizing COVID-19 convalescent plasma. Proc. Natl Acad. Sci. USA.

[44] Jones BE (2021). The neutralizing antibody, LY-CoV555, protects against SARS-CoV-2 infection in nonhuman primates. Sci. Transl. Med..

[45] Pinto D (2020). Cross-neutralization of SARS-CoV-2 by a human monoclonal SARS-CoV antibody. Nature.

[46] Shi R (2020). A human neutralizing antibody targets the receptor-binding site of SARS-CoV-2. Nature.

[47] Zahradnik, J. et al. SARS-CoV-2 RBD in vitro evolution follows contagious mutation spread, yet generates an able infection inhibitor. Preprint at https://www.biorxiv.org/content/10.1101/2021.01.06.425392v2 (2021).

[48] Zivanov, J. et al. New tools for automated high-resolution cryo-EM structure determination in RELION-3. *eLife* **7**, e42166 (2018). 10.7554/eLife.42166 PMC6250425 30412051

[49] Zheng SQ (2017). MotionCor2: anisotropic correction of beam-induced motion for improved cryo-electron microscopy. Nat. Methods.

[50] Zhang K (2016). Gctf: real-time CTF determination and correction. J. Struct. Biol..

[51] Pettersen EF (2004). UCSF Chimera—a visualization system for exploratory research and analysis. J. Comput. Chem..

[52] Wrapp D (2020). Cryo-EM structure of the 2019-nCoV spike in the prefusion conformation. Science.

[53] Sanchez-Garcia, R. et al. DeepEMhancer: a deep learning solution for cryo-EM volume post-processing. Preprint at https://www.biorxiv.org/content/10.1101/2020.06.12.148296v3 (2020). 10.1038/s42003-021-02399-1 PMC8282847 34267316

[54] Cianfrocco, M. A., Wong, M., Youn, C. & Wagner, R. COSMIC2: a science gateway for cryo-electron microscopy structure determination. *Proceedings of the Practice and Experience in Advanced Research Computing.*10.1145/3093338.3093390 (2017).

[55] Chaudhury S (2011). Benchmarking and analysis of protein docking performance in Rosetta v3.2. PLoS ONE.

[56] Huang PS (2011). RosettaRemodel: a generalized framework for flexible backbone protein design. PLoS ONE.

[57] Leaver-Fay A (2011). ROSETTA3: an object-oriented software suite for the simulation and design of macromolecules. Methods Enzymol..

[58] Goddard TD (2018). UCSF ChimeraX: meeting modern challenges in visualization and analysis. Protein Sci..

[59] Berger Rentsch M, Zimmer G (2011). A vesicular stomatitis virus replicon-based bioassay for the rapid and sensitive determination of multi-species type I interferon. PLoS ONE.

[60] Torriani G (2019). Macropinocytosis contributes to hantavirus entry into human airway epithelial cells. Virology.

[61] Torriani, G. et al. Identification of clotrimazole derivatives as specific inhibitors of arenavirus fusion. *J. Virol.* **93**, e01744-18 (2019). 10.1128/JVI.01744-18 PMC6401469 30626681

[62] Neerukonda SN (2021). Establishment of a well-characterized SARS-CoV-2 lentiviral pseudovirus neutralization assay using 293T cells with stable expression of ACE2 and TMPRSS2. PLoS ONE.

[63] Wollscheid B (2009). Mass-spectrometric identification and relative quantification of N-linked cell surface glycoproteins. Nat. Biotechnol..

[64] Huang Y (2021). Calibration of two validated SARS-CoV-2 pseudovirus neutralization assays for COVID-19 vaccine evaluation. Sci. Rep..

[65] Matsuyama S (2020). Enhanced isolation of SARS-CoV-2 by TMPRSS2-expressing cells. Proc. Natl Acad. Sci. USA.

[66] Nao N (2019). Consensus and variations in cell line specificity among human metapneumovirus strains. PLoS ONE.

[67] Li H (2009). The Sequence Alignment/Map format and SAMtools. Bioinformatics.

[68] Bolger AM, Lohse M, Usadel B (2014). Trimmomatic: a flexible trimmer for Illumina sequence data. Bioinformatics.

[69] Li H, Durbin R (2009). Fast and accurate short read alignment with Burrows–Wheeler transform. Bioinformatics.

[70] Wilm A (2012). LoFreq: a sequence-quality aware, ultra-sensitive variant caller for uncovering cell-population heterogeneity from high-throughput sequencing datasets. Nucleic Acids Res..

[71] Cingolani P (2012). A program for annotating and predicting the effects of single nucleotide polymorphisms, SnpEff: SNPs in the genome of *Drosophila melanogaster* strain *w*^*1118*^*; iso-2; iso-3*. Fly. (Austin).

[72] Gu Z, Eils R, Schlesner M (2016). Complex heatmaps reveal patterns and correlations in multidimensional genomic data. Bioinformatics.

[73] Corman, V. M. et al. Detection of 2019 novel coronavirus (2019-nCoV) by real-time RT–PCR. *Euro. Surveill.* **25**, 2000045 (2020). 10.2807/1560-7917.ES.2020.25.3.2000045 PMC6988269 31992387

[74] Gruber AD (2020). Standardization of reporting criteria for lung pathology in SARS-CoV-2-infected hamsters: what matters?. Am. J. Respir. Cell Mol. Biol..

