# Supplementary material for: The trispecific DARPin ensovibep inhibits diverse SARS-CoV-2 variants
Source: Nat Biotechnol. 2022 Jul 21;40(12):1845–54. doi: 10.1038/s41587-022-01382-3 (PMC9750863; doi:10.1038/s41587-022-01382-3)
Supplement: Supplementary file 1 — Supplementary Methods, Supplementary Notes, References for Supplementary Information, Supplementary Figs. 1–14, Supplementary Tables 1–6 and References for Supplementary Figures and Tables [file 41587_2022_1382_MOESM1_ESM.pdf]

nature portfolio

https://doi.org/10.1038/s41587-022-01382-3

Supplementary information

The trispecific DARPin ensovibep inhibits diverse SARS-CoV-2 variants

In the format provided by the
authors and unedited

Supplementary Information – NBT-RA55862B

1. Supplementary Methods

SARS-CoV-2 spike proteins variants used for ribosome display selections and screening

Proteins used for selections and screening comprised SARS-CoV-2 S protein ectodomain (SARS2-Secto-d72-GCN4-Streptag (University of Utrecht, Utrecht, Netherlands), SARS-CoV-2 S protein (S1+S2 ECT, His-tag; Sino Biological, Beijing, China, 40589-V08B1), Bio-COVID-19_S1 protein_His_Avitag (Acro Biosystems, Newark, US), SARS2-S1-Flag-3Streptag (University of Utrecht, Utrecht, Netherlands), COVID-19_S_protein_RBD_Fc (Acro Biosystems, Newark, US), and SARS2-S1B-2Streptag (University of Utrecht, Utrecht, Netherlands). Proteins were biotinylated by using standard methods.

Selection of SARS-CoV-2 spike protein-specific DARPin molecules by ribosome display

DARPin libraries[1] (N2C and N3C) were used in ribosome display selections[2,3] against the SARS-CoV-2 spike protein targets. Four selection rounds were performed for each pool. Selection stringency was continuously increased to enrich high affinity binders. In details, the following panning conditions were applied: RD panning round 1: 400 nM target concentration, 8 washes for 1 minutes in wash buffer (wash buffer composition: 50 mM Tris-HOAc (pH 7.5 at 4°C), 150 mM NaCl, 50 mM $Mg(OAc)_2$, 0.05% Tween-20), 45 PCR cycles; RD panning round 2: 100 nM target concentration, 3 washes for 1 minute in wash buffer, then 3 washes for 15 minutes in wash buffer, followed by 2 washes for 1 minute in wash buffer, 35 PCR cycles; RD panning round 3: 25 nM target concentration, 3 washes for 1 minute in wash buffer, then 3 washes for 30 minutes in wash buffer, followed by 2 washes for 1 minute in wash buffer, 30 PCR cycles; RD panning round 4: 5 nM target concentration, 3 washes for 1 minute in wash buffer, then 3 washes for 45 minutes in wash buffer, followed by 2 washes for 1 minute in wash buffer, 35 PCR cycles. PCR product of round 4 output was cloned into an expression vector for subsequent screening

Screening of monovalent DARPin molecules

Monovalent DARPin molecules specifically binding to the S1-RBD, S1-NTD and the full S-ecto domains of the spike protein were identified by a homogeneous time resolved fluorescence (HTRF) assay using crude-extracts of DARPin-expressing Escherichia coli (E. coli) cells using standard protocols. In addition to target protein binding, competition to ACE2/RBD interaction was investigated using HTRF.

Briefly, DARPin clones selected by ribosome display were cloned into a derivative of the pQE30 (Qiagen) expression vector and transformed into E. coli XL1-Blue (Stratagene), plated out on LB agar/amp andincubated overnight at 37°C. Single colonies were picked into individual wells of 96 well plates containing 165 µl growth medium (LB containing 1% glucose and 50 µg/ml ampicillin) and incubated overnight (37°C/800 rpm). 150 µl of fresh LB medium containing 50 µg/ml ampicillin was inoculated with 8.5 µl of the overnight culture for expression induced with IPTG (0.5 mM for 6 h). Cells were harvested by centrifugation of the 96-deep-well plates before resuspension in 8.5 µl B-PERII (Thermo Scientific) and incubation for 1 h at RT/600 rpm. 160 µl PBS was added and cell debris removed by centrifugation. The extract was 1:200 diluted (final concentration) in PBSTB (PBS, 0.1% Tween-20,0.2% (w/v) BSA, pH 7.4) with 20 nM biotinylated target, 1:400 anti-6His-D2 HTRF antibody (Cisbio, France) and 1:400 anti-strep-Tb (Cisbio, France) in a 384-well format (incubation: 120 min at 4°C). The plate was read with a Tecan M1000 using standard HTRF settings. The extract of each lysed clone was tested for binding to the biotinylated spike protein formats, in order to assess specific binding to the spike protein.

Cloning of multivalent DARPin molecules

Multivalent DARPin molecules were prepared using conventional cloning methods as described[4]. The individual domains are linked with proline-threonine-rich polypeptide linkers[4] flanked by glycine-serine, with a length of 24 amino acids (GSPTPTPTTPTPTPTTPTPTPTGS).

DARPin protein characterization during screening process

Characterization of monovalent DARPin molecules included SDS-PAGE, size exclusion chromatography, surface plasmon resonance, SARS-CoV-2 pseudotype virus inhibition assay, as well as live virus inhibition assay. Characterization of multivalent DARPin molecules included SDS-PAGE (fully intact size without degradation), mass spectrometry (expected molecular weight), size exclusion chromatography coupled to static light scattering (mono-dispersity and aggregation propensity), circular dichroism (melting temperature assessment), storage stability (stability at 60°C for 1 week), serum stability (stability at 37°C in serum for one week), surface plasmon resonance (affinity), SARS-CoV-2 pseudotype virus inhibition assay, live virus inhibition assay, hamster pharmacokinetic analysis, and hamster efficacy model.

Surface plasmon resonance (SRP) affinity determination

SPR assays were used to determine the binding affinity of monovalent DARPin as well as multivalent DARPin molecules to the spike protein of SARS-CoV-2. All SPR data were generated using a Bio-Rad ProteOn XPR36 instrument with PBS-T (0.005% Tween20) as running buffer. A new neutravidin sensor chip (NLC) was air-initialized and conditioned according to Bio-Rad manual.

Monovalent DARPin molecules R1, R2, R3: Chemically biotinylated (via lysines) SARS-CoV-2 spike protein 20 (Sino Biologics) was captured to ~3400 RUs (30 µg/mL, 30 µL/min, 300 s). Two buffer injections (100 µL/min, 60 s) followed by two 12.5 mM NaOH regeneration steps (100 µL/min, 18 s) were applied before the first injections. Mono-domain DARPin proteins were injected (at 50/16.7/5.6/1.9/0.6 nM) for 180 s at 100 µL/min for association and dissociation was recorded for 3600 s (at 100 µL/min). The ligand was regenerated with a 12.5 mM NaOH pulse (100 µL/min, 18 s). The data was double referenced against the empty surface and a buffer injection and fitted according to the 1:1 Langmuir model.

Multivalent DARPin molecules: Avi-tagged biotinylated SARS-CoV-2 S protein (Acro Biosystems) was captured to ~1200 RUs (1.33 ug/mL, 30 µl/min, 300 s) on a precoated neutravidin chip (NLC). Two buffer injections (100 µL/min, 60 s) followed by three 12.5 mM NaOH regeneration steps (100 µL/min, 18s) were applied before the first injections. One single concentration of 20 nM of ensovibep was injected for 180 s at 100 µL/min for association and dissociation was recorded for 36'000 s (at 100 µL/min). The data was double referenced against the empty surface and a buffer injection. Due to avidity gain, no significant dissociation could be recorded during the measured time.

Competition ELISA setup

For the competition ELISA, 10 nM of monovalent DARPin or control antibody was diluted in PBS and coated to a NUNC Maxisorb ELISA plate overnight at 4°C. After 3 automated washes with 300 µl PBST (PBS supplemented with 0.01% Tween20®) per well, the wells were blocked with 300 µl PBST supplemented with 0.2% Bovine Serum Albumin (PBST-B) and incubated for 1 hour at room temperature on a Heidolph Titramax 1000 shaker (450 rpm). Plates were again washed 3 times as described above. The competitor molecules were diluted at 500 nM in presence of 10 nM of RBD-biotinylated protein in PBS 0.01% Tween20® supplemented with 0.2% BSA and pre-incubated for 1 hour at room temperature. The blocked plate was washed as described above and 100 µl of the pre-incubated competitors with the biotinylated RBD were added as triplicates. Plates were incubated for 30 minutes at room temperature. Afterwards, the plates were washed as described above. Detection of bound biotinylated RBD was performed by the addition of streptavidin HRP reagent at a

1:10000 dilution in PBST-B, 100 ul were added in the wells and incubated 30 minutes at room temperature (at 450 rpm). HRP-linked streptavidin was detected after development using 100 µl/well peroxidase soluble substrate (30 mM Citrate buffer pH 4.1, 5% (v/v) TMB solution, from Carl Roth GmbH and 0.16% H_2O_2) for 5 minutes and stopped by the addition of 50 µl 1M H_2SO_4. The difference between the absorbance at 450 nm and the absorbance at 620 nm was measured using a Sunrise Tecan reader. The signals were analyzed with GraphPad Prism 9.1.0. The mean of the triplicates and corresponding standard deviation were represented

Whole genome sequencing of SARS-CoV-2 isolated from treated hamsters

Following RNA extraction from swabs and lung samples, libraries were prepared and sequenced using Illumina technology (Illumina, San Diego, California, USA). For library preparation, a multiplexed amplicon-based whole-viral-genome approach using the NEBNext® ARTIC SARS-CoV-2 Library Prep Kit (Illumina®) was employed (New England Biolabs, Ipswich, Massachusetts, USA). Briefly, this approach relies on cDNA synthesis from total RNA and amplification of target SARS-CoV-2 cDNA using the V3 ARTIC primers; these amplicons then undergo the usual library preparation steps for Illumina sequencing (end repair, adaptor ligation and PCR enrichment). Quantification of enriched sequencing libraries was performed using the NEBNext® Library Quant Kit for Illumina® (New England Biolabs, Ipswich, Massachussets, USA). Libraries were then pooled and sequenced on an Illumina Miseq System (Illumina, San Diego, California, USA).

The generated Illumina sequencing data were processed with Trimmomatic v.0.39[5] and mapped against genome reference MT270101.1, using the Burrows-Wheeler aligner v.0.7.17[6]. Mapping statistics were generated using Samtools v1.10[7] and alignments were visualized using IGV v2.9.4 for Linux[8]. For detection of single-nucleotide polymorphisms (SNPs), Freebayes, a Bayesian genetic variant detector was used. All SNPs with a minimum mapping quality of 5, minimum count of 3 and minimum fraction of 0.01 were considered. Consensus sequences for each sample were obtained using BCFtools. All SNP-containing open reading frame (ORFs) sequences were extracted from these consensus genomes and translated using the Expasy[98]. Translate tool. The resulting protein sequences were then aligned to the corresponding reference protein sequences using the Expasy[8] SIM Protein Alignment tool. For SNPs that resulted in amino acid substitutions, their possible effect on protein function was gauged using two predictors: PROVEAN Protein[10 11] and SIFT[12]. Results from both predictors were taken into account, except on instances where the SIFT predictor could not resolve the proposed substitution or made “low confidence” predictions, then PROVEAN’s prediction was prioritized as its protein database is larger and newer.

RNA sequencing and transcriptome analysis

Bulk RNA sequencing was performed after RNA isolation of the right medium lung lobe of infected Roborovski dwarf hamsters using Trizol reagent according to the manufacturer's instructions. Bulk RNA sequencing libraries were constructed using the NEBNext Ultra II Directional RNA Library Prep Kit (New England Biolabs) and sequenced on a Illumina NextSeq 500 device. For viral transcriptome analysis, total RNA-seq reads were mapped to the SARS-CoV-2 genome (GenBank MN908947). For host response analysis, reads were aligned to the Roborowski hamster genome[13] with hisat2[14] and gene expression quantified using the package featureCounts from Rsubread[15] and analyzed by DESeq2[16].

Hamster pharmacokinetic study

Single intraperitoneal injections of 10 mg/kg were administered to female hamsters. Fifteen animals were enrolled in each study (n=3 per time point). Blood was sampled from individual animals at 2 h, 24 h, 48 h, 72 h and 168 h post administration and processed to serum. MP0420 serum concentrations were determined by sandwich ELISA using an anti-DARPin antibody as capture reagent and biotinylated RBD and HRP conjugated Streptavidin as detection reagent and quantified against a standard curve. Serum concentrations for detection of both antibodies REGN10933 and REGN10987 were determined by sandwich ELISA using an anti-IgG antibody as capture reagent and biotinylated RBD and HRP conjugated Streptavidin as detection reagent and using a standard curve. Pharmacokinetic parameters were determined with non-compartmental analyses using the software Phoenix WinNonLin (Certara, Princeton, USA) or GraphPadPrism (GraphPad Software, La Jolla,USA). For the in vivo efficacy study, terminal bleed samples were collected at 2, 3 or 5 days p.i. according to study description.

2. Supplementary Notes

Whole genome sequencing of SARS-CoV-2 isolated from treated hamsters

To better characterize positive effects of ensovibep and mAb treatment, we performed differential gene expression analysis by bulk RNA sequencing. RNA was isolated from lung tissue of all animals that received a therapeutic treatment with ensovibep or mAb cocktail 24 h after infection. At day 2/3 p.i., the percentage of viral transcripts found in the lung tissue of dwarf hamsters treated with either ensovibep or the mAb cocktail were comparable to non-treated animals (Figure 5C). Contrary to this, total SARS-CoV-2 transcript levels among animals treated with either ensovibep or the mAb cocktail at day 5 p.i. were significantly reduced in comparison to the placebo control (Figure 5C), which indicates that comparable antiviral effects were observed for both treatments. The transcriptional levels of gene sets representing pro-inflammatory cytokines or genes involved in cytokine-mediated signaling were overall reduced after treatment of hamsters with either ensovibep or the mAb cocktail, in comparison to non-treated animals. This reduction was comparable among both treated animal groups sacrificed at day 5 p.i. (Figure 5D-F). Overall, all three gene sets, containing pro-inflammatory cytokines, genes related to type I IFN response, and response to IFNγ, showed reduced expression following both treatments when compared to non-treated animals. While the transcriptional cytokine response seems to differ to some extent between treatments, strong inter-individual differences between animals limit our ability to define characteristic transcriptional signatures related to different treatments.

Whole genome sequencing using virus RNA recovered from lungs and upper respiratory tract was performed to investigate whether SARS-CoV-2 escape mutants were selected under ensovibep treatment. Viral RNA from individual animals with higher viral load compared to other animals of the same treatment group was analyzed and no escape mutations affecting the ensovibep epitope located in the RBD were discovered (Supplementary Table 5).

3. References for Supplementary Information

1 Binz, H. K. et al. High-affinity binders selected from designed ankyrin repeat protein libraries. Nat Biotechnol **22**, 575-582, doi:10.1038/nbt962 (2004).

2 Zahnd, C., Amstutz, P. & Pluckthun, A. Ribosome display: selecting and evolving proteins in vitro that specifically bind to a target. Nat Methods **4**, 269-279, doi:10.1038/nmeth1003 (2007).

3 Hanes, J., Jermutus, L., Weber-Bornhauser, S., Bosshard, H. R. & Pluckthun, A. Ribosome display efficiently selects and evolves high-affinity antibodies in vitro from immune libraries. Proc Natl Acad Sci U S

A **95**, 14130-14135, doi:10.1073/pnas.95.24.14130 (1998).
4 Binz, H. K. et al. Design and characterization of MP0250, a tri-specific anti-HGF/anti-VEGF DARPin(R) drug candidate. MAbs **9**, 1262-1269, doi:10.1080/19420862.2017.1305529 (2017).
5 Bolger, A. M., Lohse, M. & Usadel, B. Trimmomatic: a flexible trimmer for Illumina sequence data. Bioinformatics **30**, 2114-2120, doi:10.1093/bioinformatics/btu170 (2014).
6 Li, H. & Durbin, R. Fast and accurate short read alignment with Burrows-Wheeler transform. Bioinformatics **25**, 1754-1760, doi:10.1093/bioinformatics/btp324 (2009).
7 Danecek, P. et al. Twelve years of SAMtools and BCFtools. GigaScience **10**, doi:10.1093/gigascience/giab008 (2021).
8 Robinson, J. T. et al. Integrative genomics viewer. Nat Biotechnol **29**, 24-26, doi:10.1038/nbt.1754 (2011).
9 Duvaud, S. et al. Expasy, the Swiss Bioinformatics Resource Portal, as designed by its users. Nucleic Acids Res **49**, W216-w227, doi:10.1093/nar/gkab225 (2021).
10 Choi, Y., Sims, G. E., Murphy, S., Miller, J. R. & Chan, A. P. Predicting the functional effect of amino acid substitutions and indels. PLoS One **7**, e46688, doi:10.1371/journal.pone.0046688 (2012).
11 Choi, Y. in Proceedings of the ACM Conference on Bioinformatics, Computational Biology and Biomedicine 414–417 (Association for Computing Machinery, Orlando, Florida, 2012).
12 Sim, N. L. et al. SIFT web server: predicting effects of amino acid substitutions on proteins. Nucleic Acids Res **40**, W452-457, doi:10.1093/nar/gks539 (2012).
13 Andreotti, S. et al. De Novo Whole Genome Assembly of the Roborovski Dwarf Hamster (Phodopus roborovskii) Genome, an Animal Model for Severe/Critical COVID-19. bioRxiv, 2021.2010.2002.462569, doi:10.1101/2021.10.02.462569 (2021).
14 Kim, D., Paggi, J. M., Park, C., Bennett, C. & Salzberg, S. L. Graph-based genome alignment and genotyping with HISAT2 and HISAT-genotype. Nature Biotechnology **37**, 907-915, doi:10.1038/s41587-019-0201-4 (2019).
15 Liao, Y., Smyth, G. K. & Shi, W. The R package Rsubread is easier, faster, cheaper and better for alignment and quantification of RNA sequencing reads. Nucleic Acids Research **47**, e47-e47, doi:10.1093/nar/gkz114 (2019).
16 Love, M. I., Huber, W. & Anders, S. Moderated estimation of fold change and dispersion for RNA-seq data with DESeq2. Genome Biology **15**, 550, doi:10.1186/s13059-014-0550-8 (2014).

Supplementary Figures & Tables – NBT-RA55862B

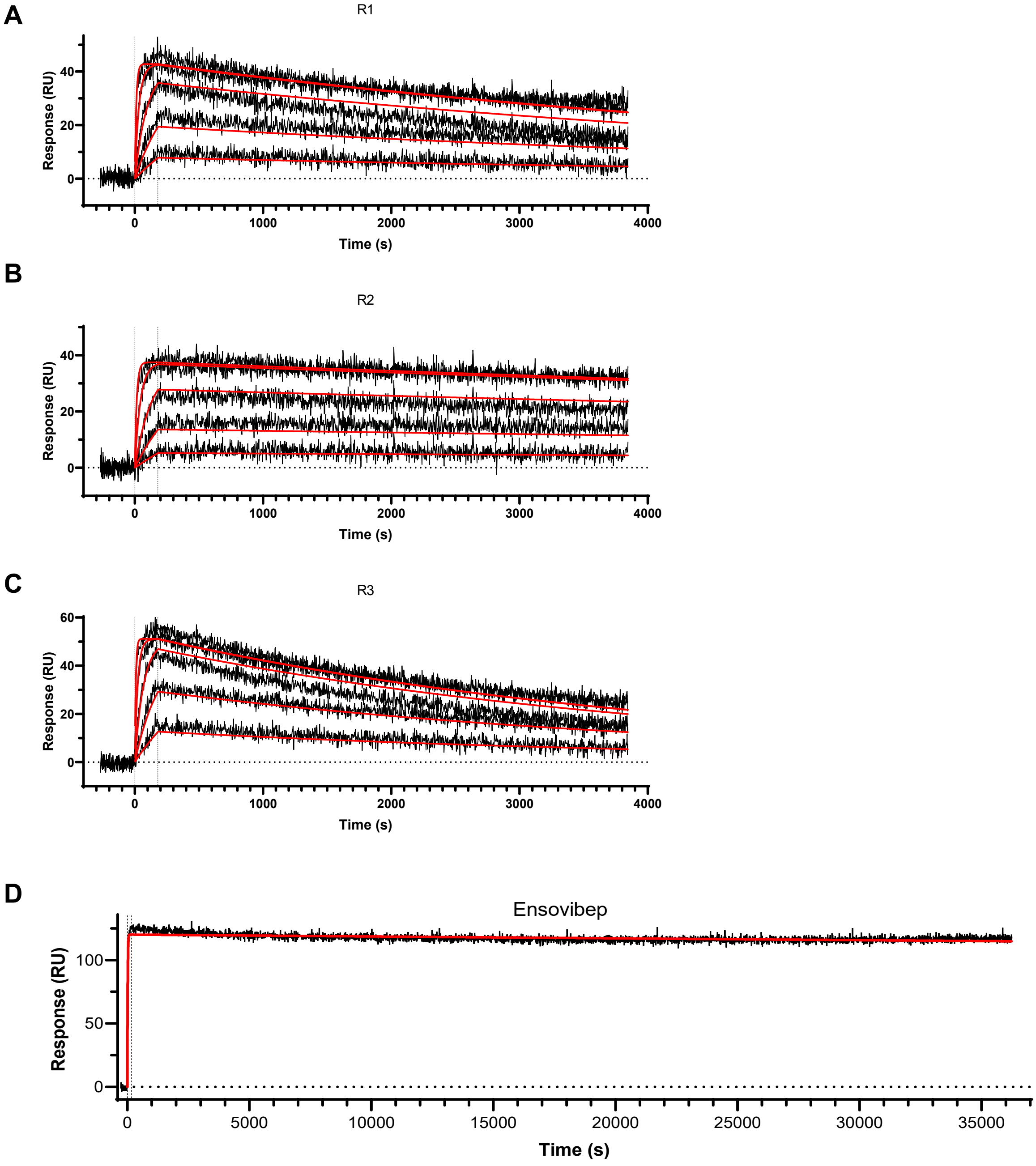

Supplementary Figure 1: A-C) Surface plasmon resonance (SPR) sensorgrams of the monovalent DARPin modules (R1, R2, R3), incorporated in ensovibep binding to immobilized trimeric spike protein. DARPin concentrations for A-C: 50/16.67/5.56/1.85/0.62 nM. Determined K_D values: A) 80 pM, B) 30 pM, C) 90 pM. D) SPR sensorgram of ensovibep binding to immobilized spike protein. Off-rate was measured over 10 h and no physical off-rate could be determined by SPR due to very strong avidity of the three interlinked RBD binding modules.

A

	**Amino Acid Sequence**
**Full Ensovibep**	(M)GSDLGKKLLEAARAGQDDEVRELLKAGADVNAKDYFSHTPLHLAARNGHLKIVEV<br>LLKAGADVNAKDFAGKTPLHLAANEGHLEIVEVLLKAGADVNAQDIFGKTPADIAADA<br>GHEDIAEVLQKAAGSPTPTPTTPTPTPTTPTPTPTGSDLGKKLLEAARAGQDDEVRE<br>LLKAGADVNAKDYFSHTPLHLAARNGHLKIVEVLLKAGADVNAKDFAGKTPLHLAAN<br>EGHLEIVEVLLKAGADVNAQDIFGKTPADIAADAGHEDIAEVLQKAAGSPTPTPTTPTP<br>TPTTPTPTPTGSDLGKKLLQAARAGQLDEVRELLKAGADVNAKDREGITPLHLAAQH<br>GHLEIVEVLLKAGADVNAKDVWGRTPLHLAAWQGHLEIVEVLLKAGADVNAKDLAGA<br>TPLHVAALYGHLEIVEVLLKAGADVNAQDKSGKTPADLAARAGHQDIAEVLQKAAGS<br>PTPTPTTPTPTPTTPTPTPTGSDLGKKLLQAARAGQLDEVRELLKAGADVNAKDREG<br>KTPLHVAAQEGHLEIVEVLLKAGADVNAKDVWGRTPLHLAAWIGHLEIVEVLLKAGAD<br>VNAKDVSGATPLHAAALHGHLEIVEVLLNAGADVNAQDKSGKTPADLAARAGHQDIA<br>EVLQKAAGSPTPTPTTPTPTPTTPTPTPTGSDLGKKLLQAARAGQLDEVRELLKAGA<br>DVNAKDQEGITPLHVAAHQGHLEIVEVLLKAGADVNAKDVWGRTPLHLAAWRGHLEI<br>VEVLLKAGADVNAKDHAGATPLHAAALSGHLEIVEVLLKAGADVNAQDKSGKTPADL<br>AARAGHQDIAEVLQKAA
**N-term initiation motif**	(M)GS
**1. DARPin domain: HSA-binder**	DLGKKLLEAARAGQDDEVRELLKAGADVNAKDYFSHTPLHLAARNGHLKIVEVLLKA<br>GADVNAKDFAGKTPLHLAANEGHLEIVEVLLKAGADVNAQDIFGKTPADIAADAGHE<br>DIAEVLQKAA
**Proline/Threonine Linker**	GSPTPTPTTPTPTPTTPTPTPTGS
**2. DARPin domain: HSA-binder**	DLGKKLLEAARAGQDDEVRELLKAGADVNAKDYFSHTPLHLAARNGHLKIVEVLLKA<br>GADVNAKDFAGKTPLHLAANEGHLEIVEVLLKAGADVNAQDIFGKTPADIAADAGHE<br>DIAEVLQKAA
**Proline/Threonine Linker**	GSPTPTPTTPTPTPTTPTPTPTGS
**3. DARPin Domain: R1**	DLGKKLLQAARAGQLDEVRELLKAGADVNAKDREGITPLHLAAQHGHLEIVEVLLKA<br>GADVNAKDVWGRTPLHLAAWQGHLEIVEVLLKAGADVNAKDLAGATPLHVAALYGH<br>LEIVEVLLKAGADVNAQDKSGKTPADLAARAGHQDIAEVLQKAA
**Proline/Threonine Linker**	GSPTPTPTTPTPTPTTPTPTPTGS
**4. DARPin Domain: R2**	DLGKKLLQAARAGQLDEVRELLKAGADVNAKDREGKTPLHVAAQEGHLEIVEVLLKA<br>GADVNAKDVWGRTPLHLAAWIGHLEIVEVLLKAGADVNAKDVSGATPLHAAALHGHL<br>EIVEVLLNAGADVNAQDKSGKTPADLAARAGHQDIAEVLQKAA
**Proline/Threonine Linker**	GSPTPTPTTPTPTPTTPTPTPTGS
**5. DARPin Domain: R3**	DLGKKLLQAARAGQLDEVRELLKAGADVNAKDQEGITPLHVAAHQGHLEIVEVLLKA<br>GADVNAKDVWGRTPLHLAAWRGHLEIVEVLLKAGADVNAKDHAGATPLHAAALSGH<br>LEIVEVLLKAGADVNAQDKSGKTPADLAARAGHQDIAEVLQKAA

B

```
consensus     - DLGKKLLQAARAGQLDEVRELLKAGADVNAKDxxGxTPLHxAAxxGHLEIVEVLLKAGAD -60
R1            - DLGKKLLQAARAGQLDEVRELLKAGADVNAKDREGITPLHLAAQHGHLEIVEVLLKAGAD -60
R2            - DLGKKLLQAARAGQLDEVRELLKAGADVNAKDREGKTPLHVAAQEGHLEIVEVLLKAGAD -60
R3            - DLGKKLLQAARAGQLDEVRELLKAGADVNAKDQEGITPLHVAAHQGHLEIVEVLLKAGAD -60

consensus     - VNAKDxxGxTPLHxAAxxGHLEIVEVLLKAGADVNAKDxxGxTPLHxAAxxGHLEIVEVL -120
R1            - VNAKDVWGRTPLHLAAWQGHLEIVEVLLKAGADVNAKDLAGATPLHVAALYGHLEIVEVL -120
R2            - VNAKDVWGRTPLHLAAWIGHLEIVEVLLKAGADVNAKDVSGATPLHAAALHGHLEIVEVL -120
R3            - VNAKDVWGRTPLHLAAWRGHLEIVEVLLKAGADVNAKDHAGATPLHAAALSGHLEIVEVL -120

consensus     - LKAGADVNAQDKSGKTPADLAARAGHQDIAEVLQKAA -157
R1            - LKAGADVNAQDKSGKTPADLAARAGHQDIAEVLQKAA -157
R2            - LNAGADVNAQDKSGKTPADLAARAGHQDIAEVLQKAA -157
R3            - LKAGADVNAQDKSGKTPADLAARAGHQDIAEVLQKAA -157
```

C

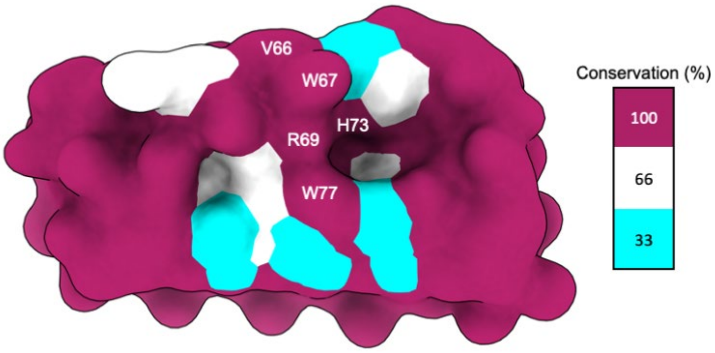

Supplementary Figure 2: A) Description of the full amino acid sequence of ensovibep and dissection of the amino acid sequence into the various component, including N-term initiation, five DARPin domains and proline/threonine linkers in between the DARPin domains. The two N-terminal, HSA-binding domains, are identical in sequence (It was shown elsewhere[1] that two HSA binders increase the systemic half-life compared to one HSA-binder). The three RBD-binding domains at the C-term share some level of homology in the interaction residues and belong to the same sequence family, containing shared interaction residues but also some part of distinct interaction residues.

B) Sequence alignment of spike binding DARPins – R1 (3rd DARPin), R2 (4th DARPin), and R3 (5th DARPin). Consensus sequence is shown with the randomized library positions indicated as "x". Residues that differ between the binders are colored in red. Interaction residues are highlighted in yellow and are determined from the analysis of distances between chains in 3D structures/models. Interaction residues are defined as those within 5 Å distance of the binding partner chain (ignoring hydrogen atoms). For R2, binding to the RBD is based on the cryoEM structure, refined computationally in Rosetta, and for the R1 and R3 DARPin domain, binding to the RBD is based on the homology information of R2 which allowed generating a computational docking model. As described in the structural analysis part, the homologous conserved fragment VWGRTPLHLAAW (underlined) interacts with the RBM loop residues F486, N487, Y489, which were shown to be important for the virus to bind ACE2 and remain highly conserved in SARS-CoV-2 variants.

C) Surface representation of the R2 DARPin domain colored according to sequence conservation of R1, R2 and R3. RBD interacting residues from the conserved fragment are indicated.

To note: Unlike monoclonal antibodies, where many are extracted from SARS patients, RBD-binders of ensovibep were selected in vitro *from artificial ribosome display DARPin libraries (see consensus sequence in panel B). The very high library diversity of 10^{12} allowed the immediate selection of picomolar binders to the spike RBD in four selection rounds[2]. In order to target an ACE2 neutralizing epitope, the binders were screened via HTRF for highest competition with human ACE2 (Supplementary Figure 14) The DARPin interaction surface residues (panel B; marked in yellow) are distributed within the amino acid sequence and embedded in the alpha helical DARPin structure. Compared to antibodies, a DARPin binding surface is relatively rigid and may target different epitope structures.*

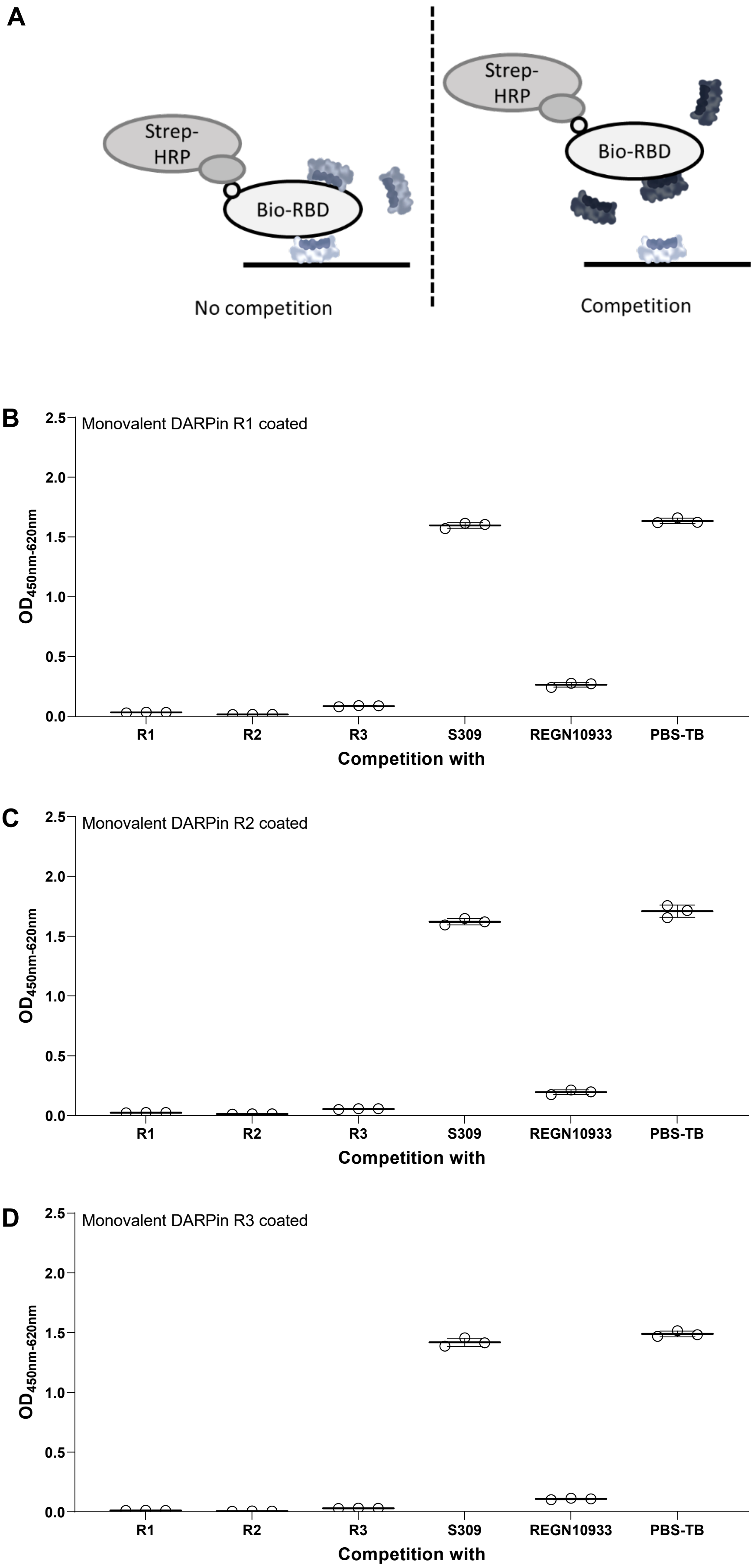
A
Strep-HRP
Bio-RBD
No competition
Strep-HRP
Bio-RBD
Competition
B
Monovalent DARPin R1 coated
C
Monovalent DARPin R2 coated
D
Monovalent DARPin R3 coated
OD450nm-620nm
0.0
0.5
1.0
1.5
2.0
2.5
R1
R2
R3
S309
REGN10933
PBS-TB
Competition with

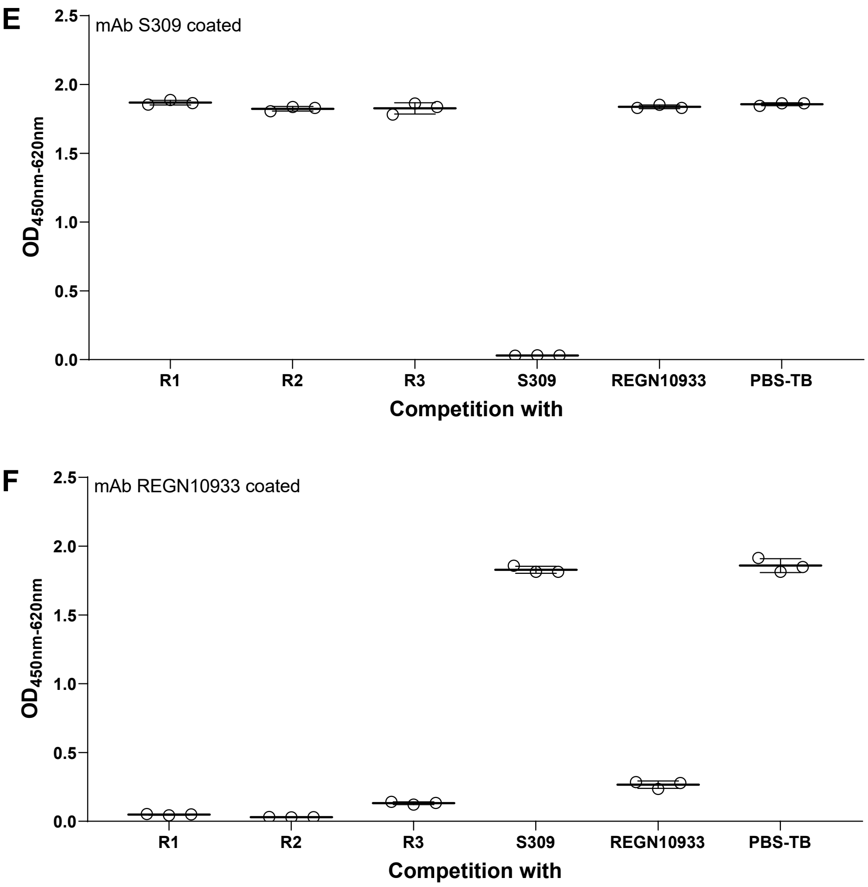

Supplementary Figure 3: Competition ELISA experiment indicates that the three monodomain RBD binders (R1; R2; R3) in ensovibep bind overlapping epitopes on the RBD of the SARS-CoV-2 spike protein. *Significant competition was observed between all three RBD binding DARPin domains (R1, R2, R3) and REGN10933 but not for S309. S309 does not compete for the same epitope on the RBD as the other binders tested.*

A) Schematic representation of the ELISA setup used to assess competition of the different molecules tested for one epitope. B) Binding competition ELISA against immobilized R1. Biotinylated SARS-CoV-2 RBD target was pre-incubated with or without competitor (R1, R2, R3, S309 or REGN10933). C) Binding competition ELISA against immobilized R2. Biotinylated SARS-CoV-2 RBD target was pre-incubated with or without competitor (R1, R2, R3, S309 or REGN10933). D) Binding competition ELISA against immobilized R3. Biotinylated SARS-CoV-2 RBD target was pre-incubated with or without competitor (R1, R2, R3, S309 or REGN10933). E) Binding Competition ELISA against immobilized S309. Biotinylated SARS-CoV-2 RBD target was pre-incubated with or without competitor (R1, R2, R3, S309 or REGN10933). F) Binding Competition ELISA against immobilized REGN10933. Biotinylated SARS-CoV-2 RBD target was pre-incubated with or without competitor (R1, R2, R3, S309 or REGN10933). For B-F, data of n=3 independent samples are represented as mean values +/- SD.

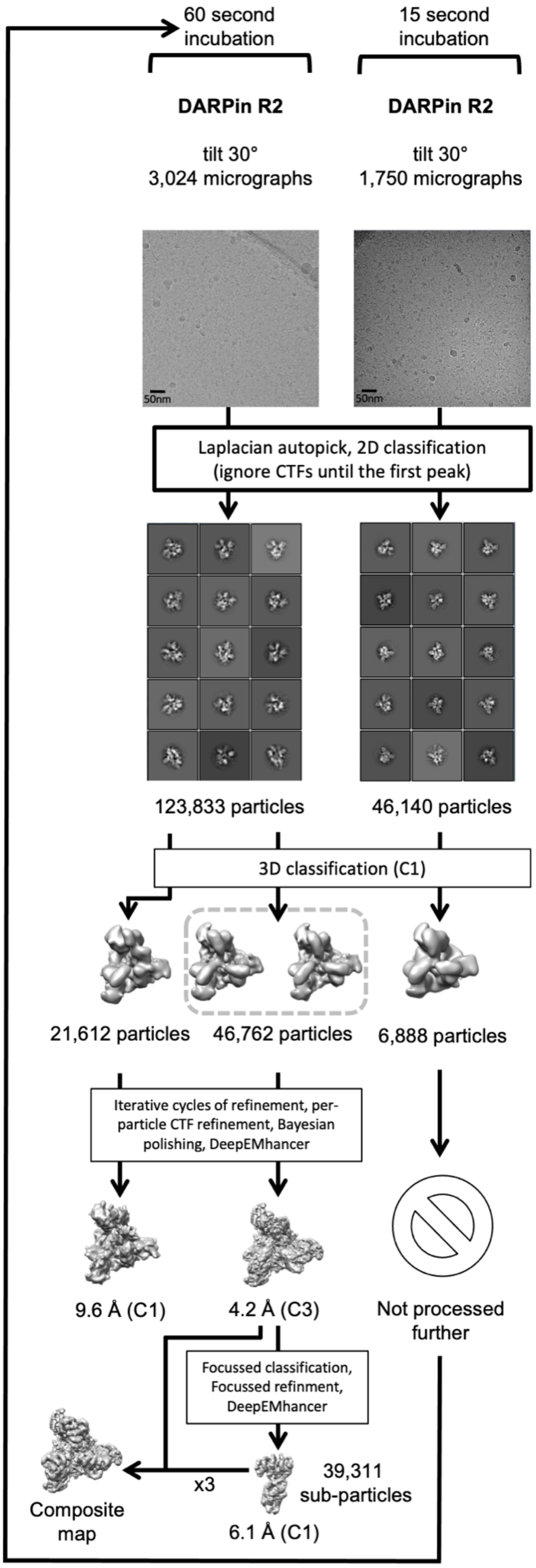

Supplementary Figure 4: Single-particle cryo-EM data processing workflow. Single-particle cryo-EM image processing workflow for the monovalent DARPin R2 data collections.

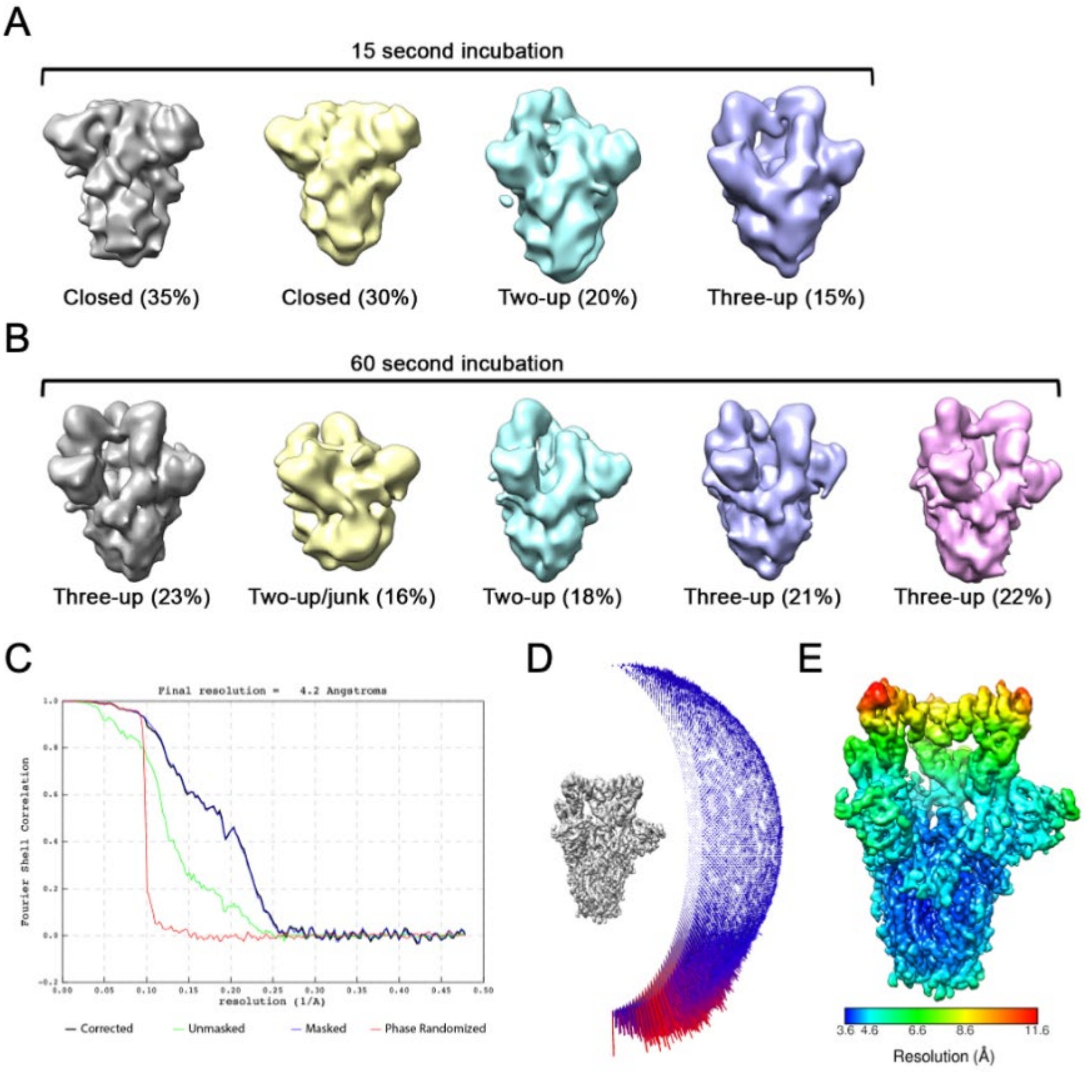

Supplementary Figure 5: Single-particle cryo-EM data processing. *A) 3D classes obtained from spike ectodomains incubated with monovalent DARPin R2 for 15 seconds, and B) for 60 seconds. C) Gold-standard Fourier shell correlation (FSC) curve generated from the independent half maps contributing to the 4.2 Å resolution density map. D) Angular distribution plot of the final C3 refined EM density map. E) The EM density map of the spike ectodomain bound to three copies of monovalent DARPin R2, colored according to local resolution.*

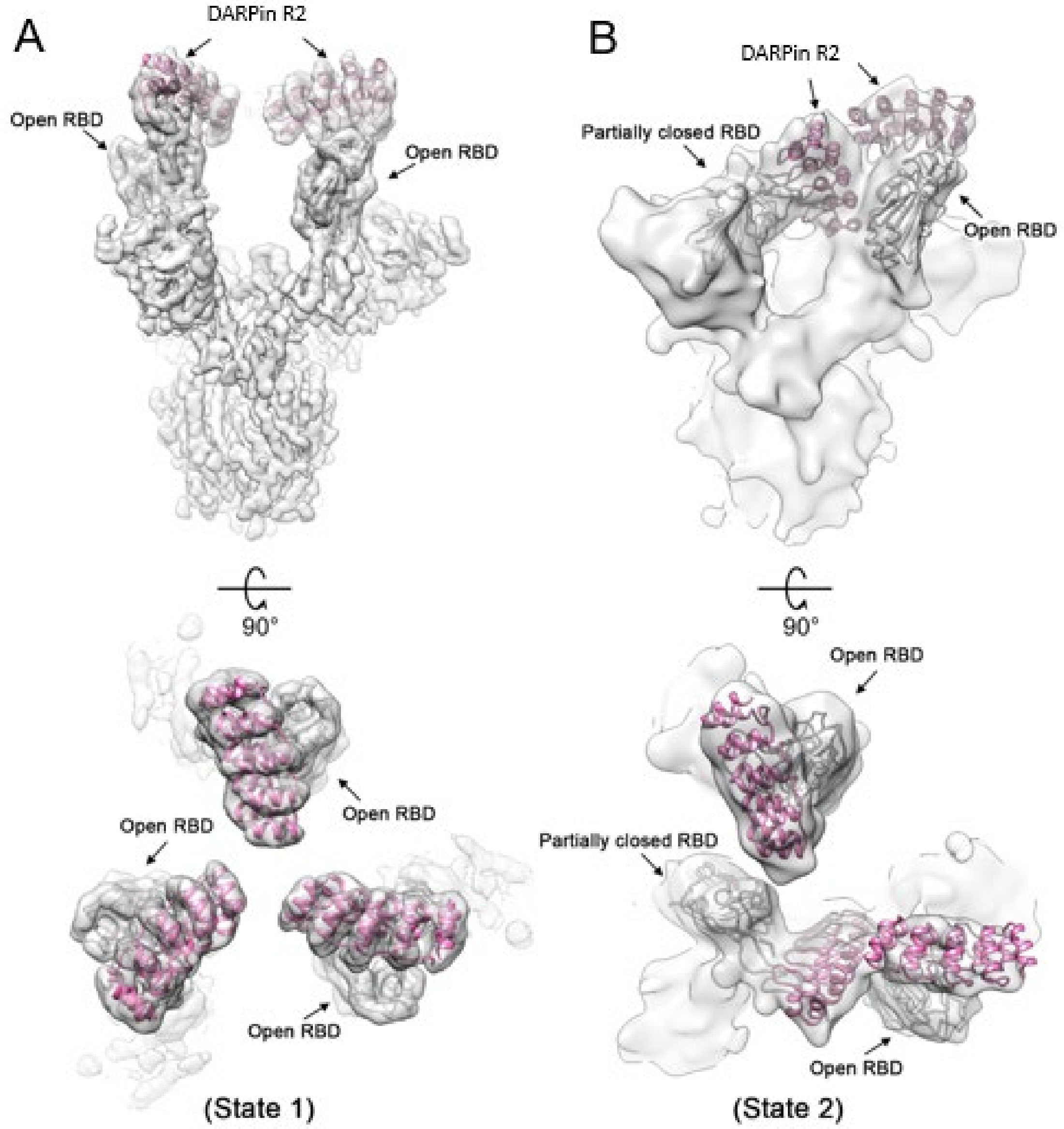

Supplementary Figure 6: Monovalent DARPin R2 prevents full closure of the RBD. A) Cryo-EM density for state 1 and B) state 2 of the SARS-CoV-2 spike ectodomain in complex with the RBD targeting monovalent DARPin R2, shown as two orthogonal views. The pseudo-atomic model of monovalent DARPin R2 in complex with RBD, derived from molecular docking experiments, is fitted in each of the spike protomers and colored grey and pink, respectively.

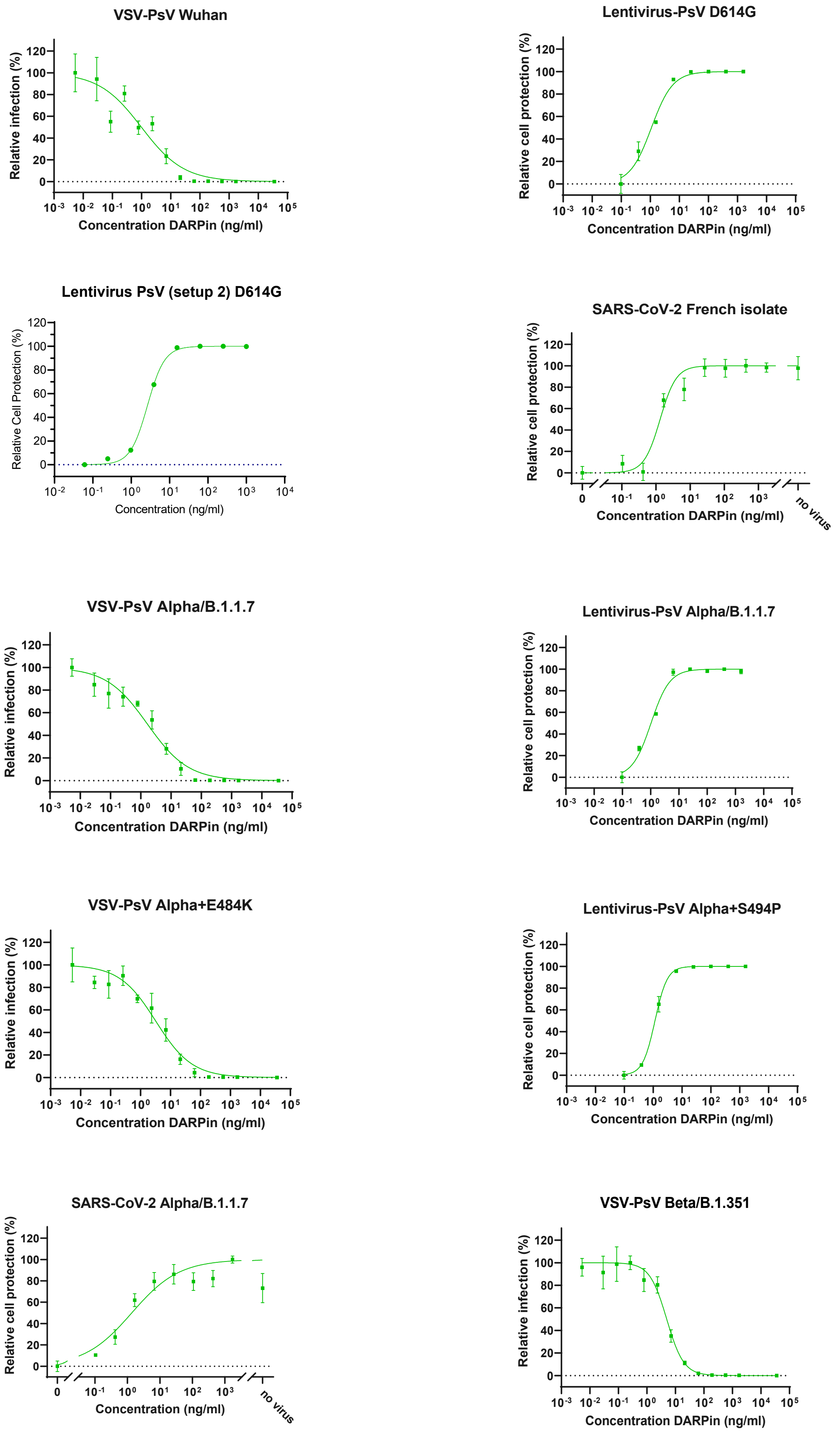

VSV-PsV Wuhan
Relative infection (%)
Concentration DARPin (ng/ml)
Lentivirus-PsV D614G
Relative cell protection (%)
Concentration DARPin (ng/ml)
Lentivirus PsV (setup 2) D614G
Relative Cell Protection (%)
Concentration (ng/ml)
SARS-CoV-2 French isolate
Relative cell protection (%)
Concentration DARPin (ng/ml)
no virus
VSV-PsV Alpha/B.1.1.7
Relative infection (%)
Concentration DARPin (ng/ml)
Lentivirus-PsV Alpha/B.1.1.7
Relative cell protection (%)
Concentration DARPin (ng/ml)
VSV-PsV Alpha+E484K
Relative infection (%)
Concentration DARPin (ng/ml)
Lentivirus-PsV Alpha+S494P
Relative cell protection (%)
Concentration DARPin (ng/ml)
SARS-CoV-2 Alpha/B.1.1.7
Relative cell protection (%)
Concentration (ng/ml)
no virus
VSV-PsV Beta/B.1.351
Relative infection (%)
Concentration DARPin (ng/ml)

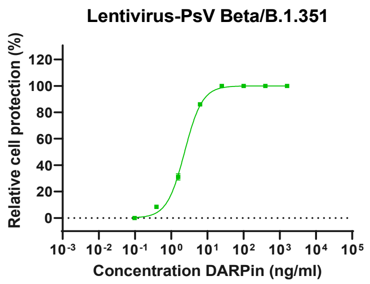
Lentivirus-PsV Beta/B.1.351
Relative cell protection (%)
Concentration DARPin (ng/ml)

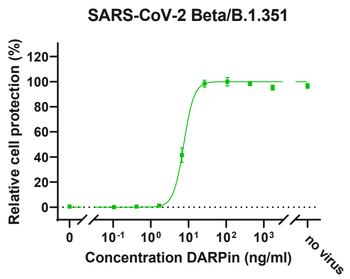
SARS-CoV-2 Beta/B.1.351
Relative cell protection (%)
Concentration DARPin (ng/ml)
no virus

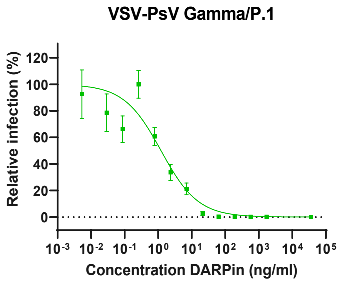
VSV-PsV Gamma/P.1
Relative infection (%)
Concentration DARPin (ng/ml)

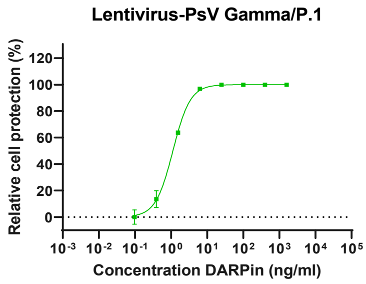
Lentivirus-PsV Gamma/P.1
Relative cell protection (%)
Concentration DARPin (ng/ml)

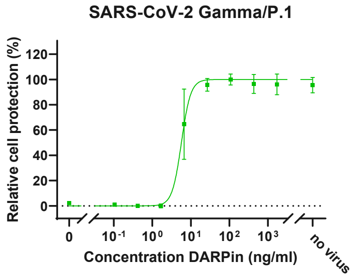
SARS-CoV-2 Gamma/P.1
Relative cell protection (%)
Concentration DARPin (ng/ml)
no virus

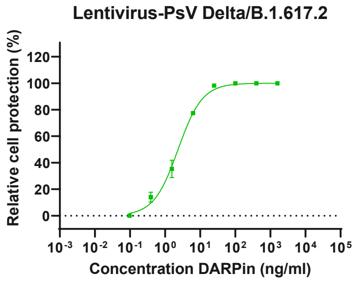
Lentivirus-PsV Delta/B.1.617.2
Relative cell protection (%)
Concentration DARPin (ng/ml)

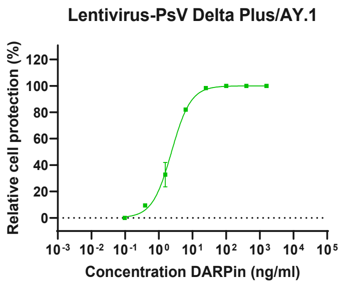
Lentivirus-PsV Delta Plus/AY.1
Relative cell protection (%)
Concentration DARPin (ng/ml)

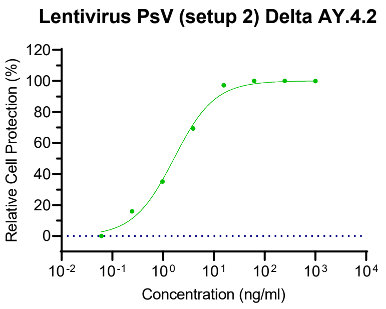
Lentivirus PsV (setup 2) Delta AY.4.2
Relative Cell Protection (%)
Concentration (ng/ml)

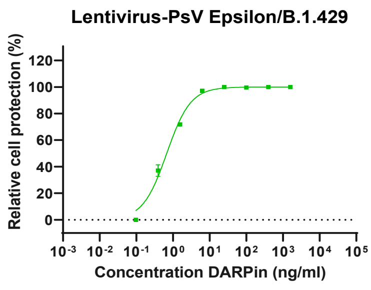
Lentivirus-PsV Epsilon/B.1.429
Relative cell protection (%)
Concentration DARPin (ng/ml)

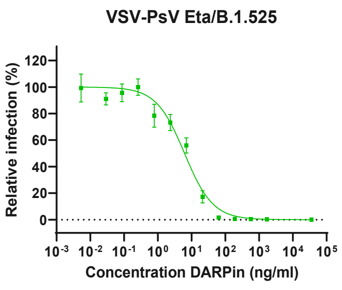
VSV-PsV Eta/B.1.525
Relative infection (%)
Concentration DARPin (ng/ml)

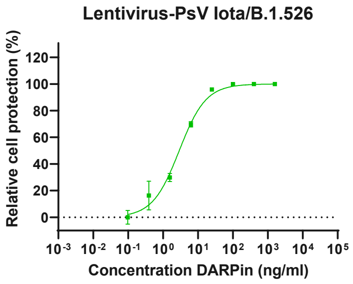
Lentivirus-PsV Iota/B.1.526
Relative cell protection (%)
Concentration DARPin (ng/ml)

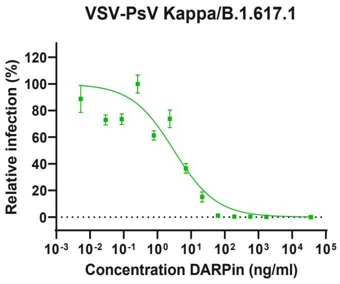
VSV-PsV Kappa/B.1.617.1
Relative infection (%)
Concentration DARPin (ng/ml)

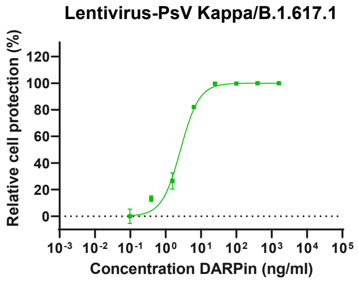
Lentivirus-PsV Kappa/B.1.617.1
Relative cell protection (%)
Concentration DARPin (ng/ml)

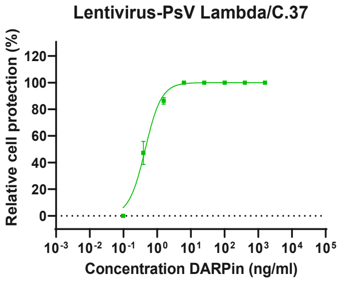
Lentivirus-PsV Lambda/C.37
Relative cell protection (%)
Concentration DARPin (ng/ml)

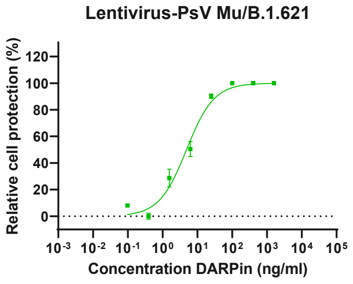
Lentivirus-PsV Mu/B.1.621
Relative cell protection (%)
Concentration DARPin (ng/ml)

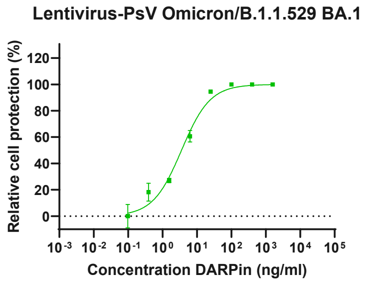
Lentivirus-PsV Omicron/B.1.1.529 BA.1
Relative cell protection (%)
Concentration DARPin (ng/ml)

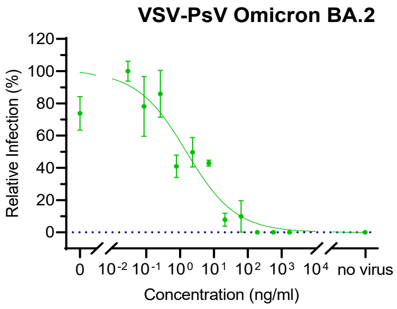
VSV-PsV Omicron BA.2
Relative Infection (%)
no virus
Concentration (ng/ml)

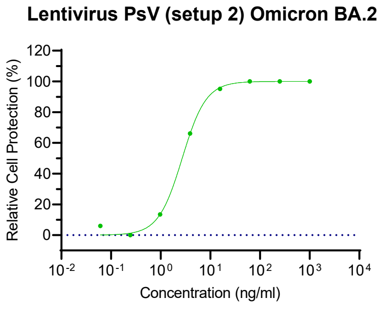
Lentivirus PsV (setup 2) Omicron BA.2
Relative Cell Protection (%)
Concentration (ng/ml)

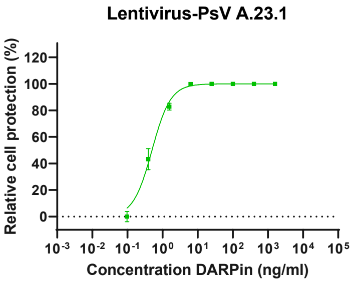
Lentivirus-PsV A.23.1
Relative cell protection (%)
Concentration DARPin (ng/ml)

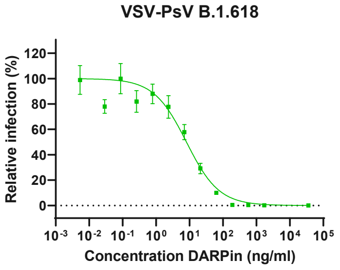
VSV-PsV B.1.618
Relative infection (%)
Concentration DARPin (ng/ml)

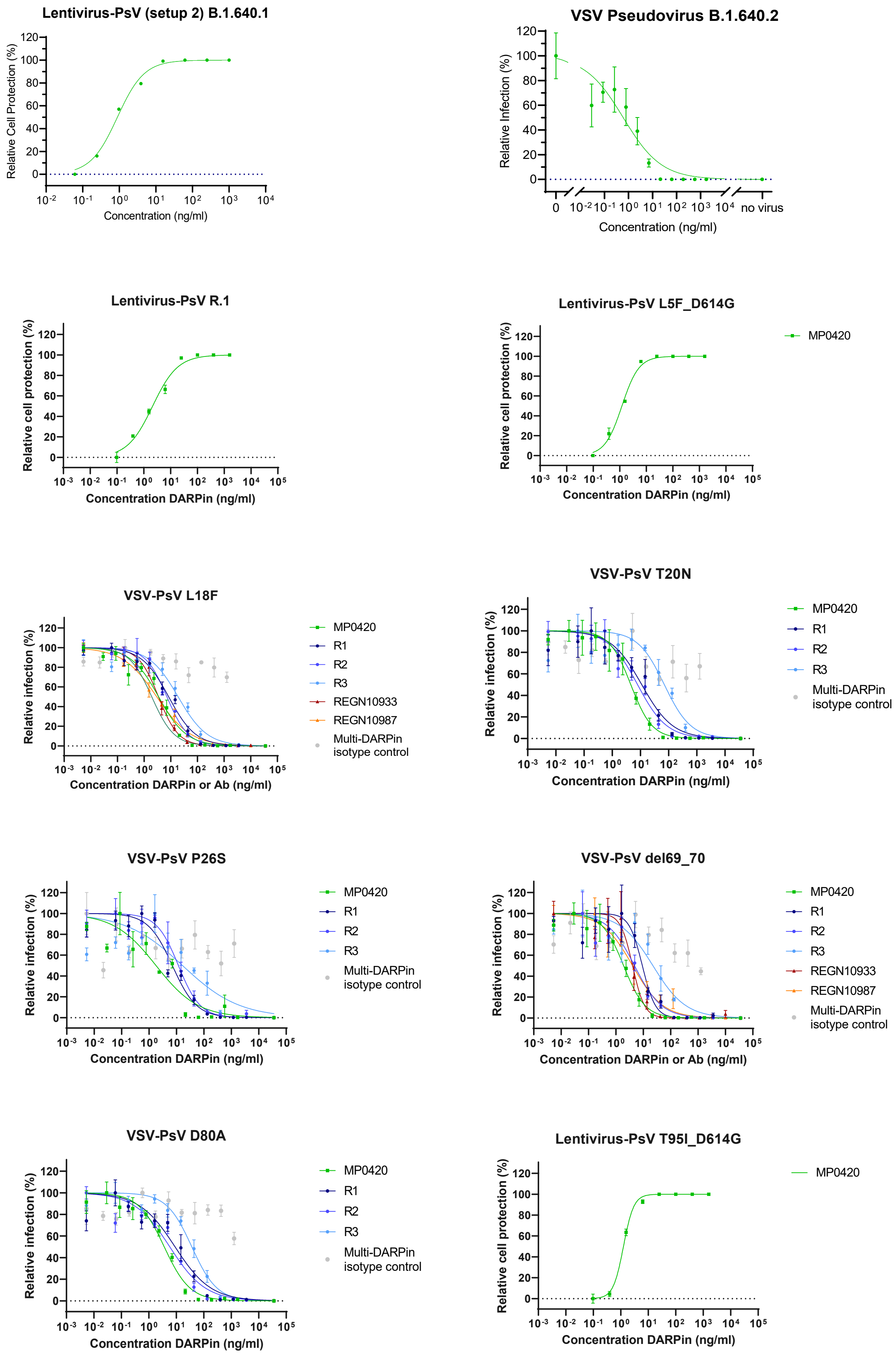

Lentivirus-PsV (setup 2) B.1.640.1
Relative Cell Protection (%)
Concentration (ng/ml)
VSV Pseudovirus B.1.640.2
Relative Infection (%)
no virus
Concentration (ng/ml)
Lentivirus-PsV R.1
Relative cell protection (%)
Concentration DARPin (ng/ml)
Lentivirus-PsV L5F_D614G
Relative cell protection (%)
Concentration DARPin (ng/ml)
MP0420
VSV-PsV L18F
Relative infection (%)
Concentration DARPin or Ab (ng/ml)
MP0420
R1
R2
R3
REGN10933
REGN10987
Multi-DARPin isotype control
VSV-PsV T20N
Relative infection (%)
Concentration DARPin (ng/ml)
MP0420
R1
R2
R3
Multi-DARPin isotype control
VSV-PsV P26S
Relative infection (%)
Concentration DARPin (ng/ml)
MP0420
R1
R2
R3
Multi-DARPin isotype control
VSV-PsV del69_70
Relative infection (%)
Concentration DARPin or Ab (ng/ml)
MP0420
R1
R2
R3
REGN10933
REGN10987
Multi-DARPin isotype control
VSV-PsV D80A
Relative infection (%)
Concentration DARPin (ng/ml)
MP0420
R1
R2
R3
Multi-DARPin isotype control
Lentivirus-PsV T95I_D614G
Relative cell protection (%)
Concentration DARPin (ng/ml)
MP0420

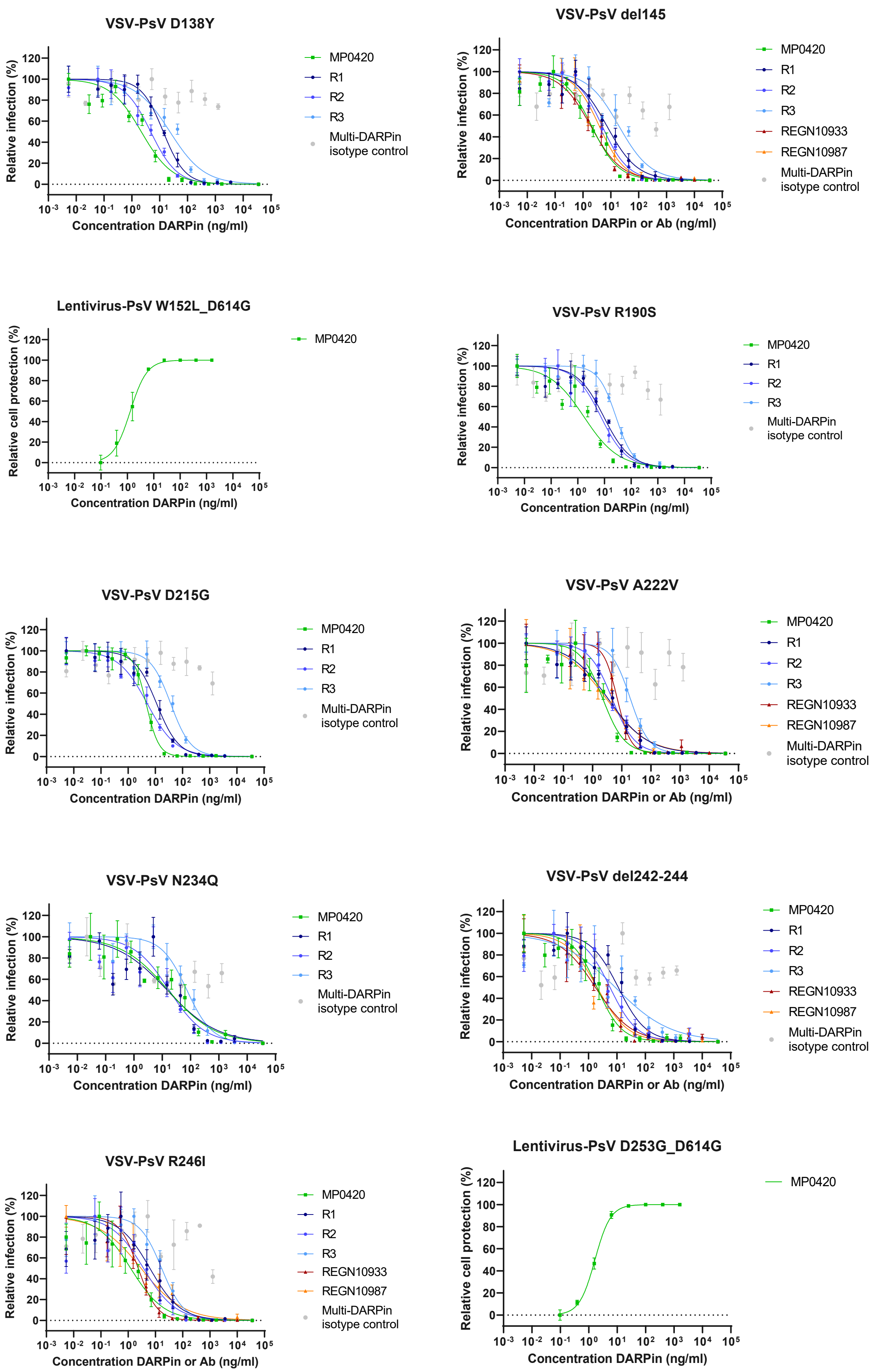
VSV-PsV D138Y
Relative infection (%)
Concentration DARPin (ng/ml)
MP0420
R1
R2
R3
Multi-DARPin isotype control
VSV-PsV del145
Relative infection (%)
Concentration DARPin or Ab (ng/ml)
MP0420
R1
R2
R3
REGN10933
REGN10987
Multi-DARPin isotype control
Lentivirus-PsV W152L_D614G
Relative cell protection (%)
Concentration DARPin (ng/ml)
MP0420
VSV-PsV R190S
Relative infection (%)
Concentration DARPin (ng/ml)
MP0420
R1
R2
R3
Multi-DARPin isotype control
VSV-PsV D215G
Relative infection (%)
Concentration DARPin (ng/ml)
MP0420
R1
R2
R3
Multi-DARPin isotype control
VSV-PsV A222V
Relative infection (%)
Concentration DARPin or Ab (ng/ml)
MP0420
R1
R2
R3
REGN10933
REGN10987
Multi-DARPin isotype control
VSV-PsV N234Q
Relative infection (%)
Concentration DARPin (ng/ml)
MP0420
R1
R2
R3
Multi-DARPin isotype control
VSV-PsV del242-244
Relative infection (%)
Concentration DARPin or Ab (ng/ml)
MP0420
R1
R2
R3
REGN10933
REGN10987
Multi-DARPin isotype control
VSV-PsV R246I
Relative infection (%)
Concentration DARPin or Ab (ng/ml)
MP0420
R1
R2
R3
REGN10933
REGN10987
Multi-DARPin isotype control
Lentivirus-PsV D253G_D614G
Relative cell protection (%)
Concentration DARPin (ng/ml)
MP0420

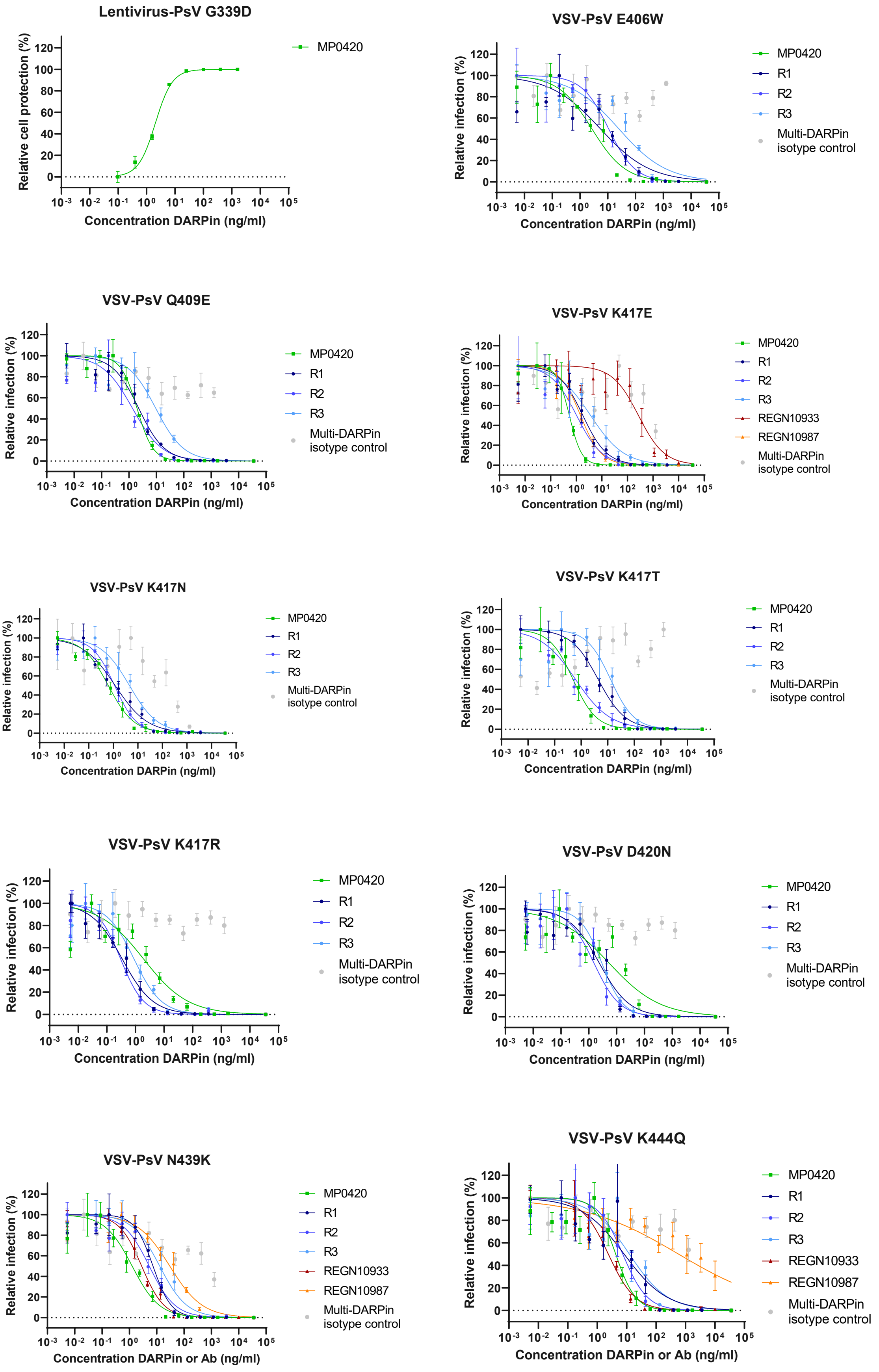
Lentivirus-PsV G339D
Relative cell protection (%)
Concentration DARPin (ng/ml)
MP0420
VSV-PsV E406W
VSV-PsV Q409E
VSV-PsV K417E
VSV-PsV K417N
VSV-PsV K417T
VSV-PsV K417R
VSV-PsV D420N
VSV-PsV N439K
VSV-PsV K444Q
Relative infection (%)
Concentration DARPin (ng/ml)
Concentration DARPin or Ab (ng/ml)
MP0420
R1
R2
R3
REGN10933
REGN10987
Multi-DARPin isotype control

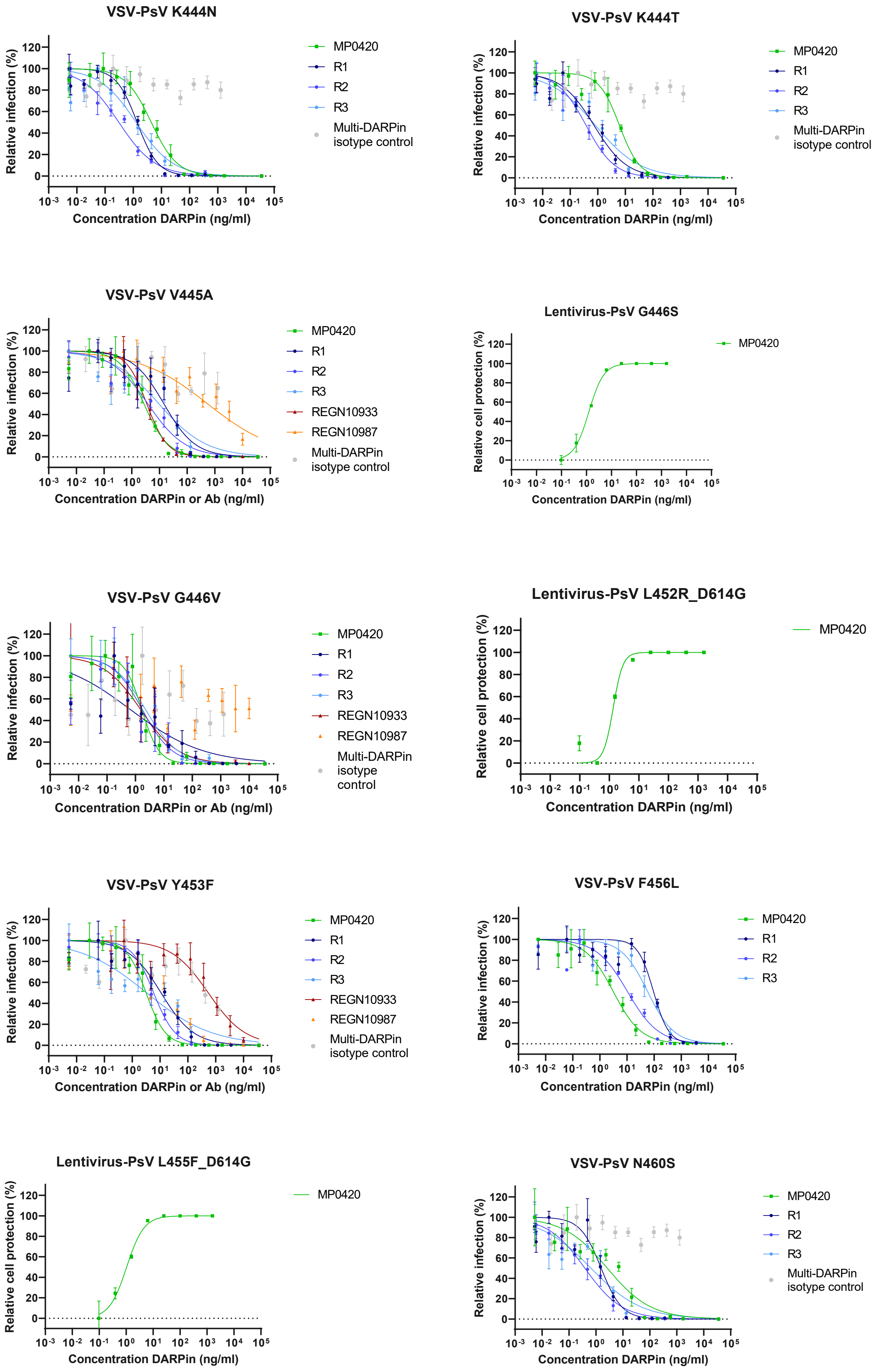
VSV-PsV K444N
Relative infection (%)
Concentration DARPin (ng/ml)
MP0420
R1
R2
R3
Multi-DARPin isotype control
VSV-PsV K444T
Relative infection (%)
Concentration DARPin (ng/ml)
MP0420
R1
R2
R3
Multi-DARPin isotype control
VSV-PsV V445A
Relative infection (%)
Concentration DARPin or Ab (ng/ml)
MP0420
R1
R2
R3
REGN10933
REGN10987
Multi-DARPin isotype control
Lentivirus-PsV G446S
Relative cell protection (%)
Concentration DARPin (ng/ml)
MP0420
VSV-PsV G446V
Relative infection (%)
Concentration DARPin or Ab (ng/ml)
MP0420
R1
R2
R3
REGN10933
REGN10987
Multi-DARPin isotype control
Lentivirus-PsV L452R_D614G
Relative cell protection (%)
Concentration DARPin (ng/ml)
MP0420
VSV-PsV Y453F
Relative infection (%)
Concentration DARPin or Ab (ng/ml)
MP0420
R1
R2
R3
REGN10933
REGN10987
Multi-DARPin isotype control
VSV-PsV F456L
Relative infection (%)
Concentration DARPin (ng/ml)
MP0420
R1
R2
R3
Lentivirus-PsV L455F_D614G
Relative cell protection (%)
Concentration DARPin (ng/ml)
MP0420
VSV-PsV N460S
Relative infection (%)
Concentration DARPin (ng/ml)
MP0420
R1
R2
R3
Multi-DARPin isotype control

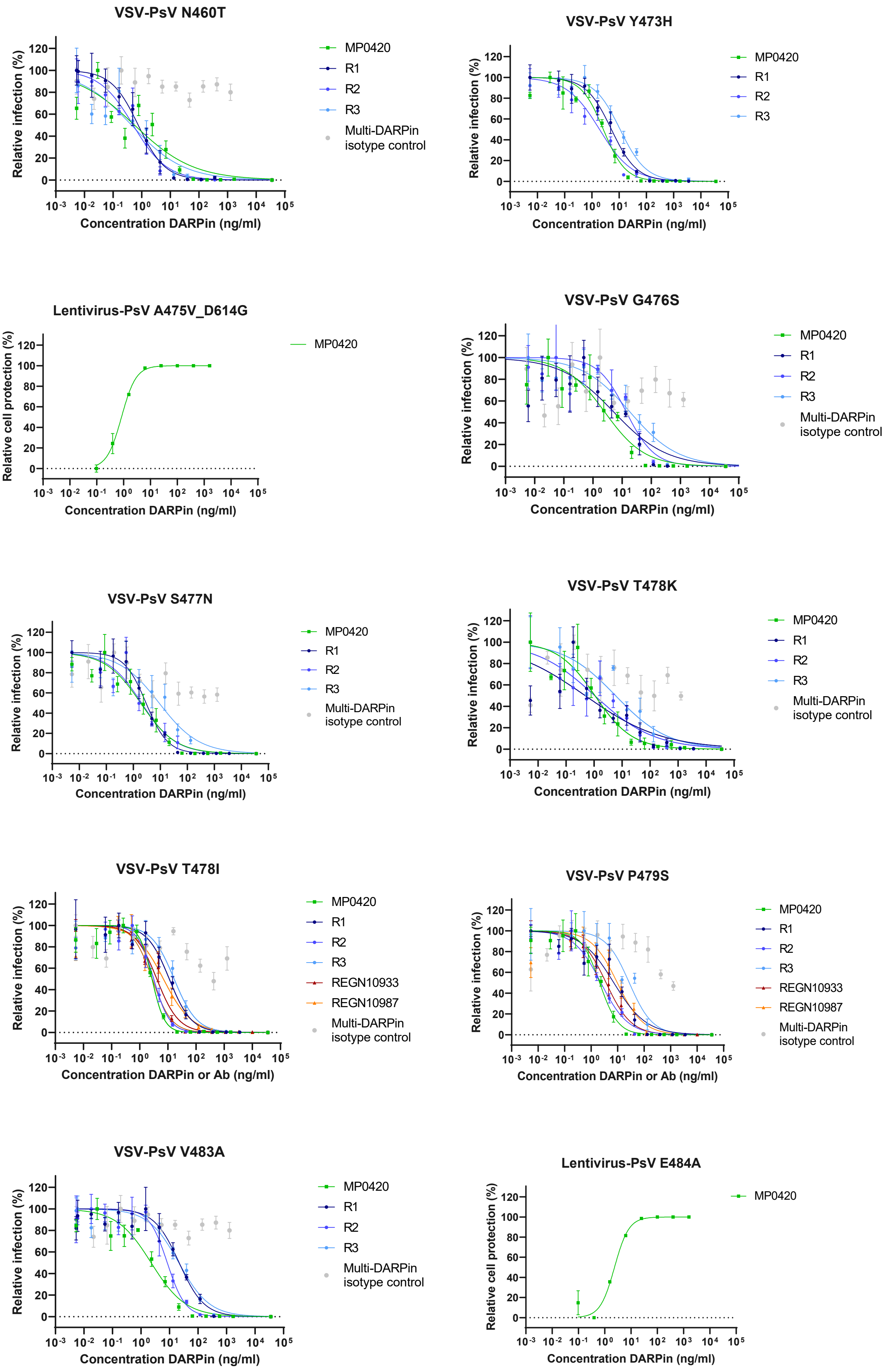
VSV-PsV N460T
Relative infection (%)
Concentration DARPin (ng/ml)
MP0420
R1
R2
R3
Multi-DARPin isotype control
VSV-PsV Y473H
Relative infection (%)
Concentration DARPin (ng/ml)
MP0420
R1
R2
R3
Lentivirus-PsV A475V_D614G
Relative cell protection (%)
Concentration DARPin (ng/ml)
MP0420
VSV-PsV G476S
Relative infection (%)
Concentration DARPin (ng/ml)
MP0420
R1
R2
R3
Multi-DARPin isotype control
VSV-PsV S477N
Relative infection (%)
Concentration DARPin (ng/ml)
MP0420
R1
R2
R3
Multi-DARPin isotype control
VSV-PsV T478K
Relative infection (%)
Concentration DARPin (ng/ml)
MP0420
R1
R2
R3
Multi-DARPin isotype control
VSV-PsV T478I
Relative infection (%)
Concentration DARPin or Ab (ng/ml)
MP0420
R1
R2
R3
REGN10933
REGN10987
Multi-DARPin isotype control
VSV-PsV P479S
Relative infection (%)
Concentration DARPin or Ab (ng/ml)
MP0420
R1
R2
R3
REGN10933
REGN10987
Multi-DARPin isotype control
VSV-PsV V483A
Relative infection (%)
Concentration DARPin (ng/ml)
MP0420
R1
R2
R3
Multi-DARPin isotype control
Lentivirus-PsV E484A
Relative cell protection (%)
Concentration DARPin (ng/ml)
MP0420

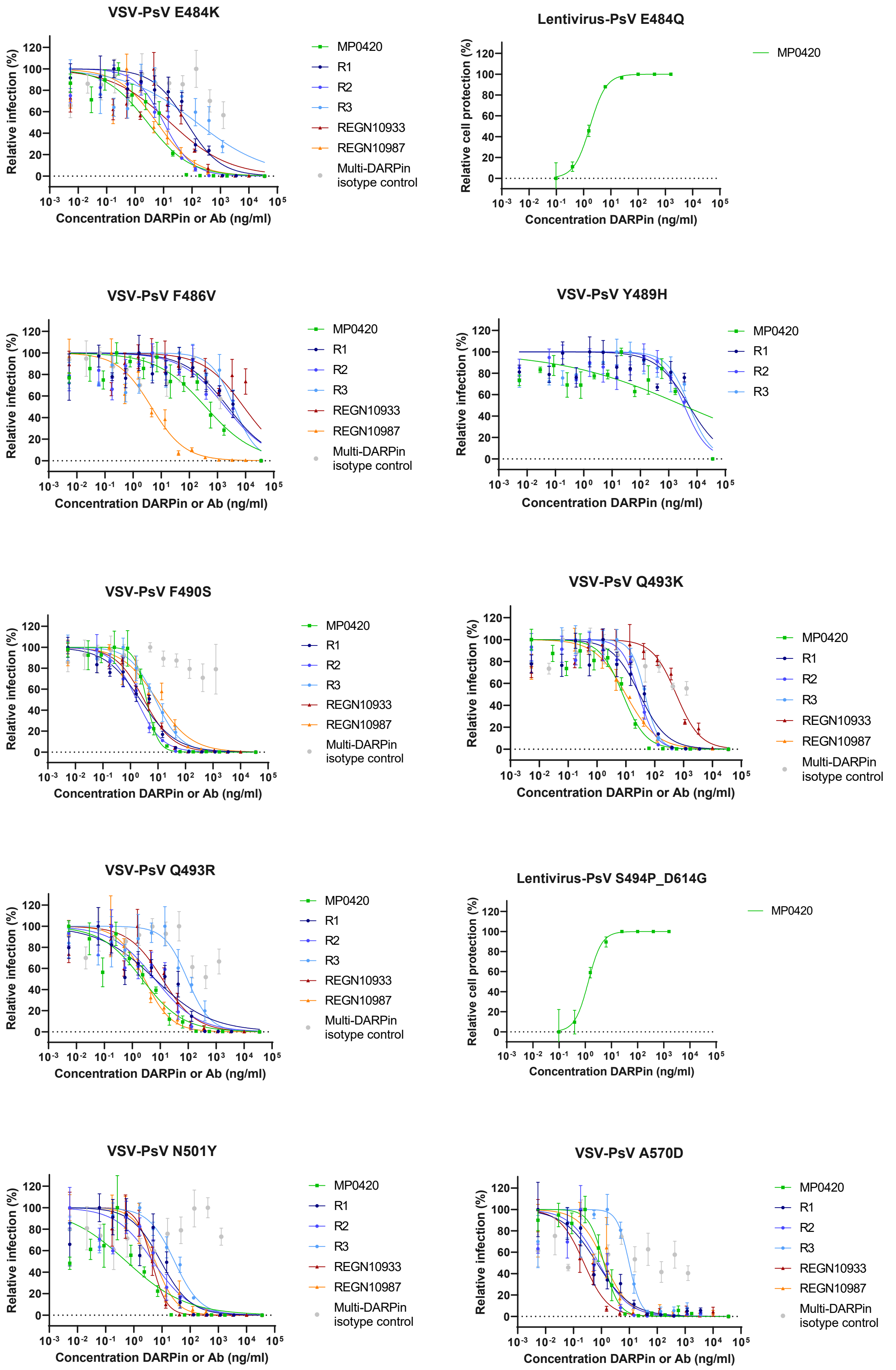
VSV-PsV E484K
Relative infection (%)
Concentration DARPin or Ab (ng/ml)
MP0420
R1
R2
R3
REGN10933
REGN10987
Multi-DARPin isotype control
Lentivirus-PsV E484Q
Relative cell protection (%)
Concentration DARPin (ng/ml)
MP0420
VSV-PsV F486V
Relative infection (%)
Concentration DARPin or Ab (ng/ml)
MP0420
R1
R2
R3
REGN10933
REGN10987
Multi-DARPin isotype control
VSV-PsV Y489H
Relative infection (%)
Concentration DARPin (ng/ml)
MP0420
R1
R2
R3
VSV-PsV F490S
Relative infection (%)
Concentration DARPin or Ab (ng/ml)
MP0420
R1
R2
R3
REGN10933
REGN10987
Multi-DARPin isotype control
VSV-PsV Q493K
Relative infection (%)
Concentration DARPin or Ab (ng/ml)
MP0420
R1
R2
R3
REGN10933
REGN10987
Multi-DARPin isotype control
VSV-PsV Q493R
Relative infection (%)
Concentration DARPin or Ab (ng/ml)
MP0420
R1
R2
R3
REGN10933
REGN10987
Multi-DARPin isotype control
Lentivirus-PsV S494P_D614G
Relative cell protection (%)
Concentration DARPin (ng/ml)
MP0420
VSV-PsV N501Y
Relative infection (%)
Concentration DARPin or Ab (ng/ml)
MP0420
R1
R2
R3
REGN10933
REGN10987
Multi-DARPin isotype control
VSV-PsV A570D
Relative infection (%)
Concentration DARPin or Ab (ng/ml)
MP0420
R1
R2
R3
REGN10933
REGN10987
Multi-DARPin isotype control

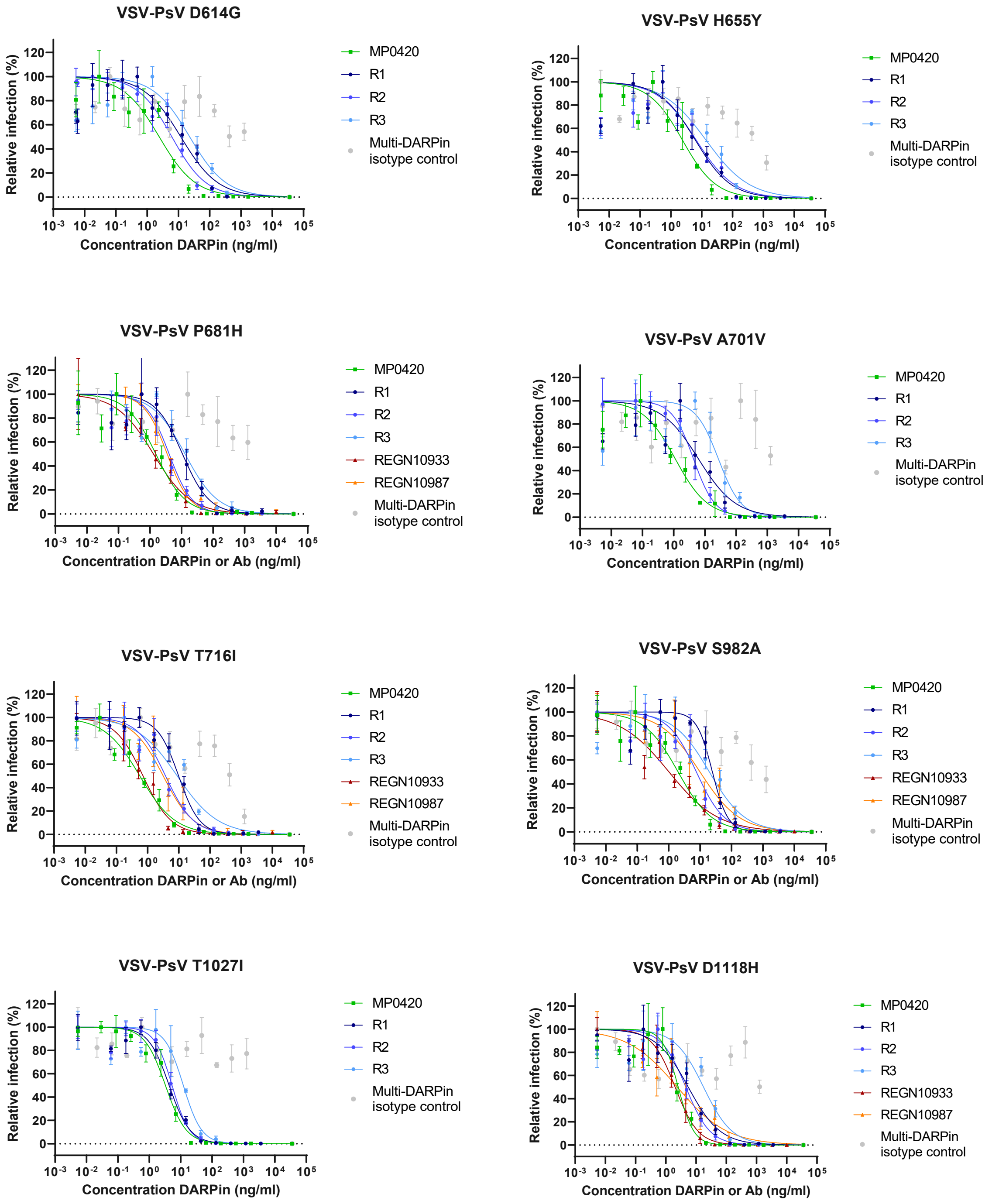

Supplementary Figure 7: Titration curves for ensovibep (MP0420) and its RBD-binding domains (i.e. R1, R2 and R3), REGN10933 and REGN10987 to determine IC_{50} neutralization potencies on multiple spike mutants or only for ensovibep (MP0420) on the variants, which are summarized in Figure 2, Table 2 and Table 3. For lentivirus-based pseudotype assays the mean +/− SEM (standard error of the mean) of n=2 biologically independent samples are represented (exception for setup 2, n=1). For VSV-based pseudotype assays the mean +/− SEM (standard error of the mean) of n=4 biologically independent samples are represented.

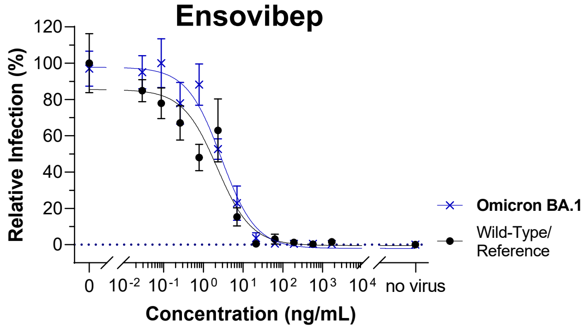

B

	Wild-Type	Omicron BA.1	Fold-Change
	IC_{50} (ng/mL)	IC_{50} (ng/mL)	IC_{50} (ng/mL)
**Ensovibep**	2.2	2.8	**1.3**
**R1**	8.8	359.6	**40.7**
**R2**	0.8	5.4	**9.9**
**R3**	11.93	496.8	**41.6**

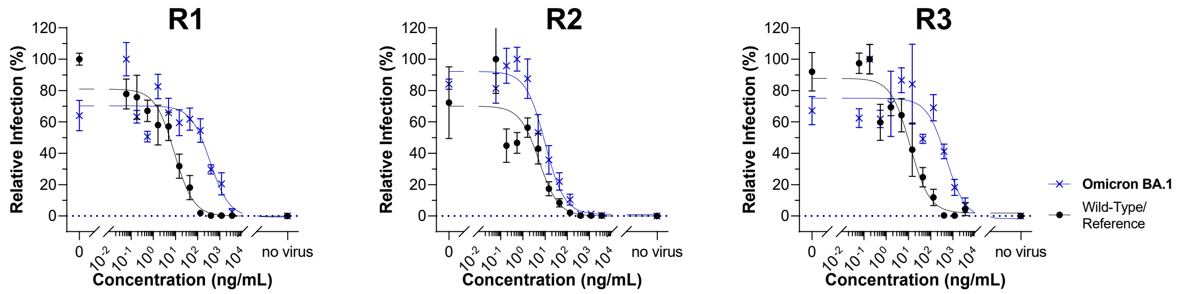

Supplementary Figure 8: VSV-pseudotype relative infection assay titration curves of the full ensovibep molecule (A) and the individual ensovibep DARPin domains (C-E) against Wuhan-hu-1 wild-type (WT) and the omicron BA.1 variant of SARS-CoV-2 spike protein. IC50 values for WT and omicron BA.1 as well as the fold-change between the omicron BA.1 and the WT IC50 values are given in the table in panel B. Reduction in potency on the omicron variant BA.1 is observed for each individual DARPin domain (R1, 40.7-fold / R2, 9.9-fold / R3, 41.6-fold). Pseudotype omicron BA.1 neutralization by the trispecific ensovibep, containing cooperative binding of all three domains retains potency (1.3-fold, compared to WT). For VSV-based pseudotype assays in A-D, the mean +/− SEM (standard error of the mean) of n=4 biologically independent samples are represented.

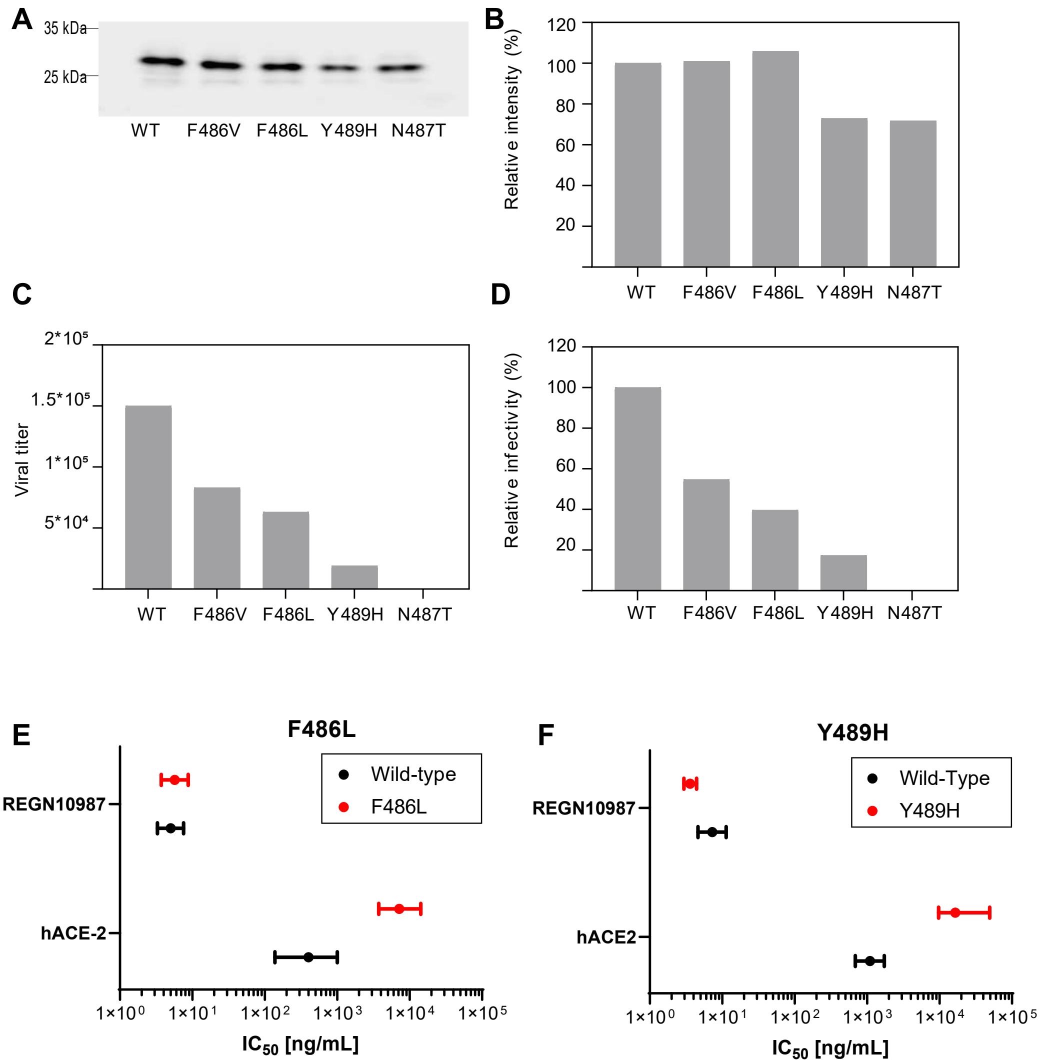

Supplementary Figure 9: Impact of SARS-CoV-2 spike protein substitutions at positions F486, N487 and Y489 on SARS-CoV-2 infectivity

Characterization of VSV-Pseudotype (PsV) neutralization when the SARS-CoV-2 spike protein in mutated at position F486, N487 or Y489, the key binding residues for ensovibep. (A) Quantitative assessment of viral particles. Following pseudovirus production, supernatants were suspended in equal volumes of SDS-PAGE sample buffer. Equal relative amount of pseudoviral samples were assessed by Western blotting using anti-VSV M antibody. Signals of VSV M (26 kDa) were quantified by densitometry. (B) Quantification of (A). VSV-M signals in the blots in (A) were quantified by densitometry. Data were obtained in arbitrary densitometric units (DU), normalized to WT and represented in relative intensity (%). (C) Titer of SARS-CoV-2 VSV-PsV. SARS-CoV-2 VSV-PsV WT and containing key residues (F486V, F486L, Y489H, N487T) were serial diluted and incubated with monolayers of Vero E6 cells. Titers were determined by counting the EGFP reporter by direct immunofluorescence. Representative titers are indicated. (D) Relative infectivity of pseudovirus. Viral titers were normalized to the amount of VSV M (A) and represented in percentage. While relative infectivities for substitutions at F486 and Y489 dropped substantially when compared to wild-type, the N487H substituted pseudotype infectivity was too low to perform solid neutralization experiments.

(E,F) Determination of IC_{50} values for human ACE2 and REGN10987 for neutralization of a VSV pseudotype with SARS-CoV-2 wild type spike protein compared to substitutions in positions F486 (E) and Y489 (F). To note: both mutated residues are key residues for the virus to interact with human ACE2, while REGN10987 (not bind in this epitope region) was used as a control. While no potency loss was

observed for REGN10987, neutralization of the F486 and Y489 substituted virus was significantly lowered. Reported is the mean +/− 95% confidence intervals of n=4 biologically independent samples. Presented data in panels A-F are further supported by very low frequencies of mutations in positions F486, N487 and Y489 in the SARS-CoV-2 spike protein sequences counted in the more than seven million sequences of the GISAID database (January 2022; F486, 0.0079% / N487, 0.0025% / Y489, 0.0031%)

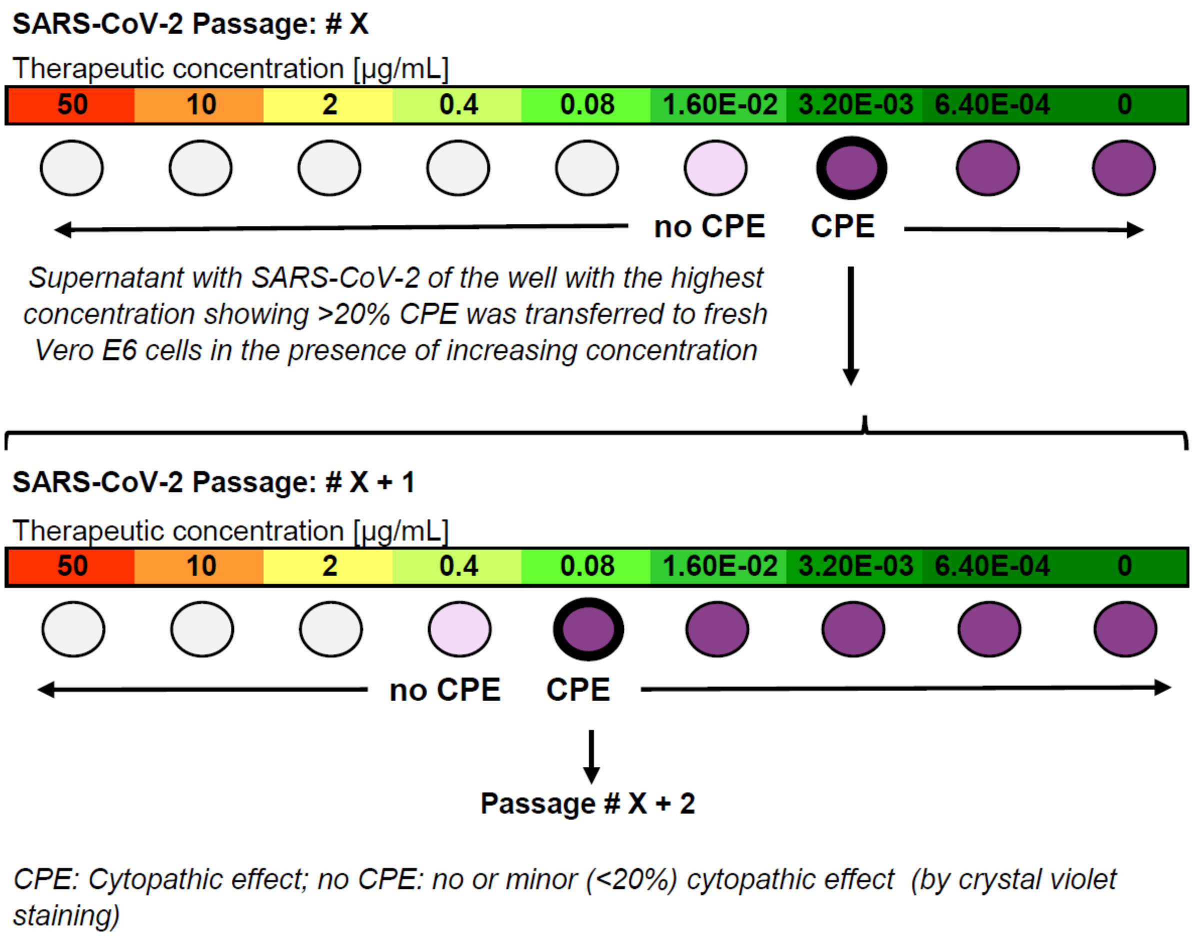

Supplementary Figure 10: Overview of the experimental protocol for viral passaging: A patient SARS-CoV-2 isolate from early 2020 (1.5 ×10^6 pfu) was incubated in presence of increasing concentrations of DARPin candidate or antibody for 4 days on Vero E6 cells and virus-induced cytopathic effects (CPE) were determined by microscopy. For each DARPin and antibody condition, cultures showing significant cytopathic effect (≥20%) under the greatest selective pressure were selected and virus-containing supernatant collected to start a new culture passage on Vero E6 cells (bold circle), again under increasing concentrations of the corresponding DARPin candidate or antibody condition. Passaging of virus containing supernatant was continued in the same manner for a total of 4 passages.

A

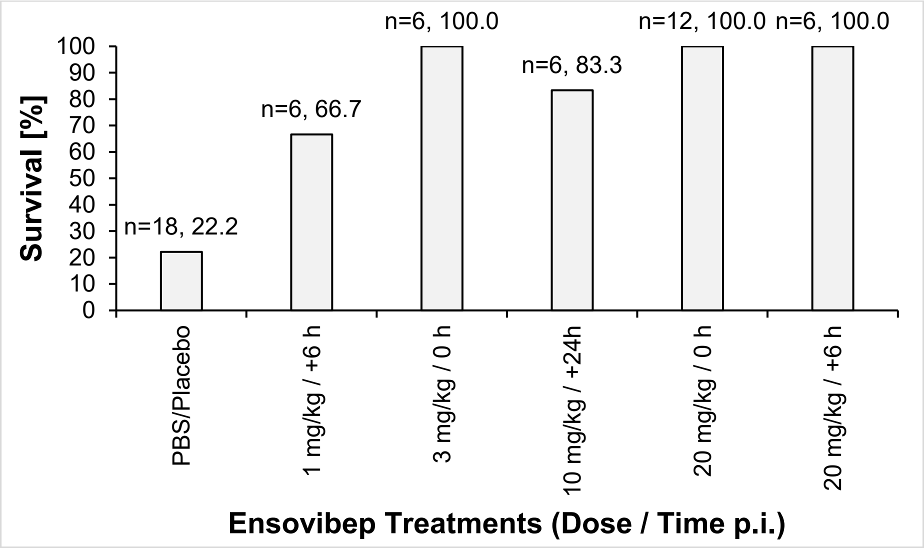

B

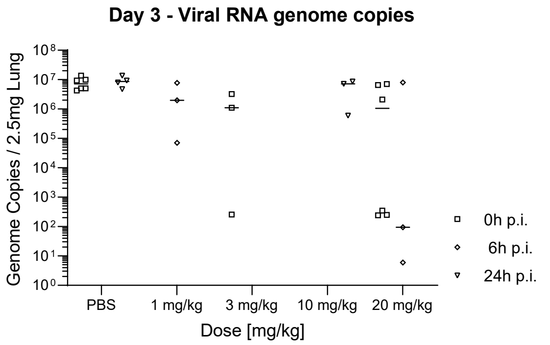

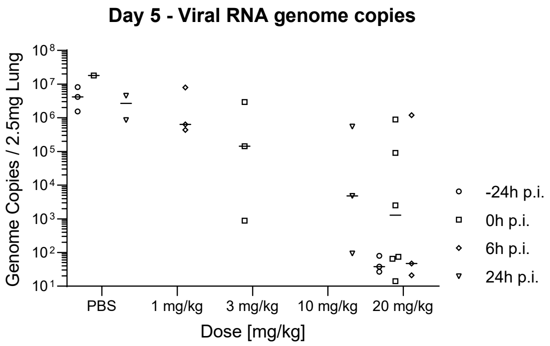

C

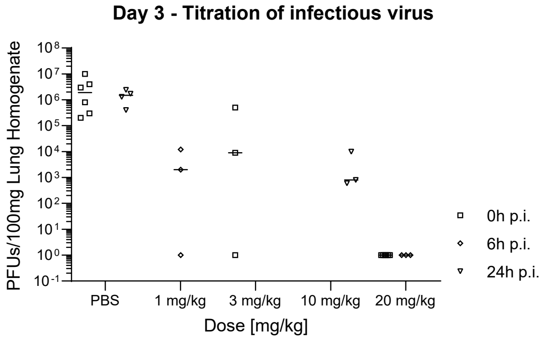

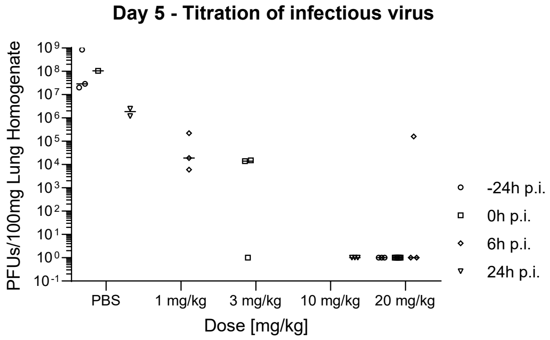

Supplementary Figure 11: Summarized previous in vivo studies with Roborovski dwarf hamster infected with WT SARS-CoV-2 and treated with ensovibep at various doses and administration time points. A) Animal survival, end-point analysis, animals that had to be euthanized according to score sheet criteria were considered non-survived, animals that reached their respective defined take-out at day 3 or 5 post infection were considered survived. B) qPCR analysis of virus gRNA copy numbers in oropharyngeal swabs and lung homogenates at day 3 or day 5 post infection. (For statistical evaluation a Mann-Whitney test between PBS and different doses was performed. Due to the small numbers, all

administration time-points were pooled per dose group. Day 3: 1 mg/kg not significant; 3 mg/kg not significant; 10 mg/kg $p<0.05$; 20mg/kg $p<0.001$ / Day 5: 1 mg/kg not significant; 3 mg/kg $p<0.01$; 10 mg/kg not significant; 20mg/kg $p<0.01$) C) Titration of replication competent virus from lung homogenates as plaque assay on Vero E6 cells at day 3 or day 5 post infection. Reported are the values of the individual animals and the median. (For statistical evaluation a Mann-Whitney test between PBS and different doses was performed. Due to the small numbers, all administration time-points were pooled per dose group. Day 3: 1 mg/kg $p<0.01$; 3 mg/kg $p<0.05$; 10 mg/kg $p<0.05$; 20mg/kg $p<0.0001$ / Day 5: 1 mg/kg $p<0.05$; 3 mg/kg $p<0.05$; 10 mg/kg $p<0.05$; 20mg/kg $p<0.0001$)

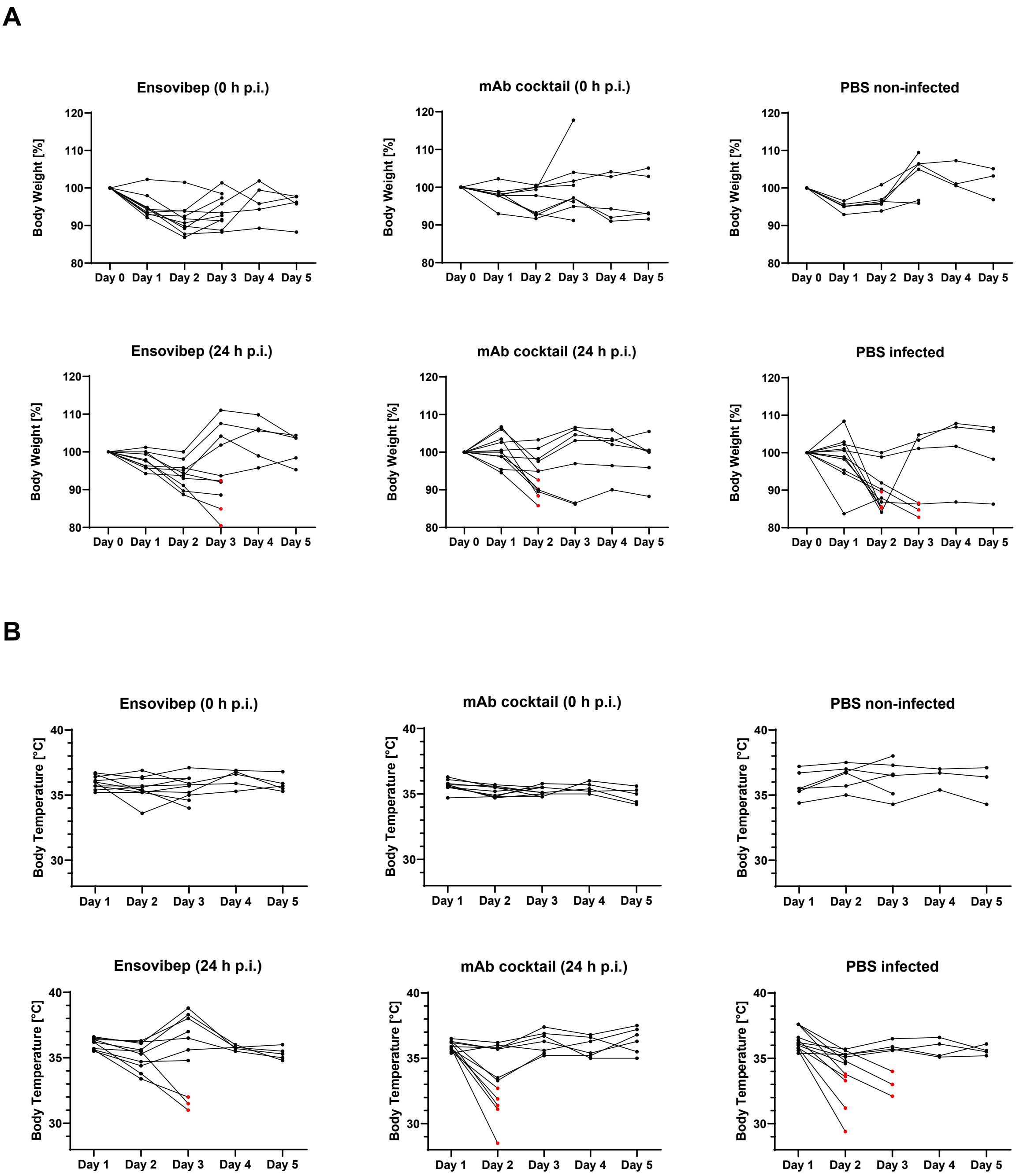

Supplementary Figure 12: Clinical Parameters of individuals over the course of infection, (mean +/- SD presented in Figure 5C) A) Body weight changes of individual hamsters B) Body temperatures of individual hamsters. Animals that had to be euthanized based on score sheet criteria are marked in red.

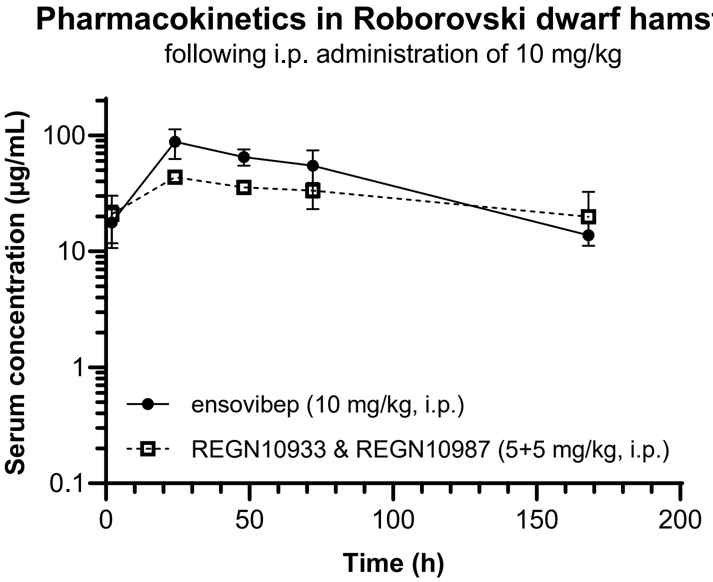

Supplementary Figure 13: *Pharmacokinetics profiles of non-infected Roborovski dwarf hamsters injected i.p. with either 10 mg/kg of ensovibep or the cocktail of REGN10933 and REGN10987 at 5 mg/kg for each of the monoclonal antibodies. Three animals were sacrificed for determination of the therapeutic concentration in the serum of the terminal bleeds. Obvious outliers due to likely a failure of the intraperitoneal injection were removed from the evaluation. Reported is the mean +/− SEM (standard error of the mean) of n=3 experimentally independent animals for each time-point and treatment. Pharmacokinetic parameters for ensovibep: $T_{1/2}$: 52.0 h; C_{max}: 87.8 µg/mL; T_{max}: 24 h. Pharmacokinetic parameters for the cocktail of REGN10933 and REGN10987: $T_{1/2}$: 139 h; C_{max}: 43.5 µg/mL; T_{max}: 24h.*

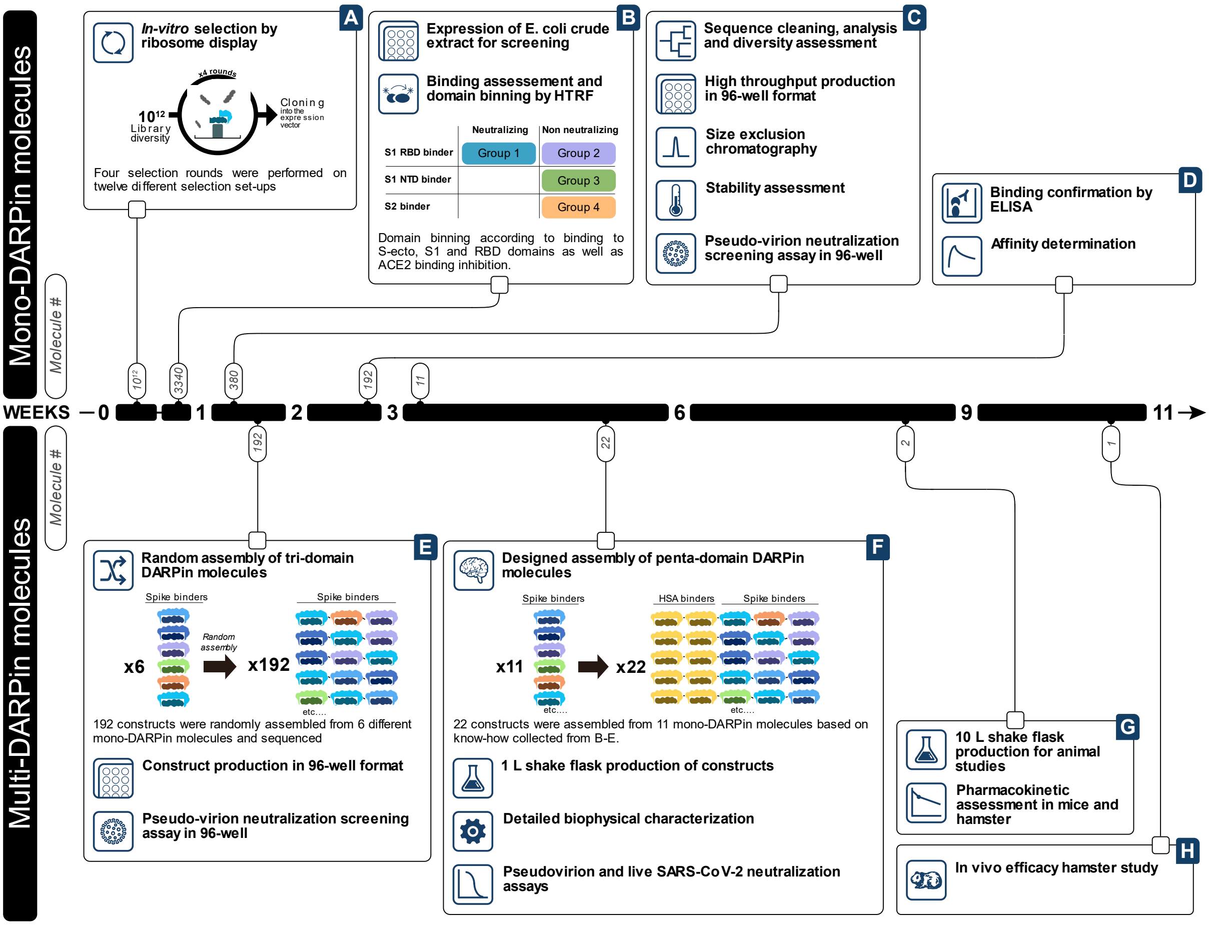

*Supplementary Figure 14: **Process overview for the generation of anti-SARS-CoV-2 multivalent DARPin molecules.** Upper panels (A-D), generation and evaluation of monovalent DARPin molecules. Lower panels (E-H), assembly and deep-characterization of multivalent DARPin molecules, resulting in the generation and in vivo proof of concept for ensovibep, 11 weeks after the initiation of the in vitro selection process.*

Supplementary Table 1: Cryo-EM data collection and image processing information.

**Incubation time (seconds)**	**60**	**60**	**15**
**Magnification**	75,000	75,000	92,000
**Voltage** [3]	300	300	200
**Electron exposure (e-/Å^2)**	40	40	40
**Defocus range (µm)**	1.25-2.5	1.25-2.5	1.25-2.5
**Pixel size (Å)**	1.045	1.045	1.1
**Symmetry imposed**	C3	C1	N/A
**Initial particle images (no.)**	123,833	123,833	46,140
**Final particle images (no.)**	46,762	21,612	6,888
**Map resolution (Å)**	4.2	9.6	N/A
**FSC threshold**	0.143	0.143	N/A
**Map resolution range (Å)**	3.6-14.1	8.2-26	N/A

Supplementary Table 2: *In vitro protection against emerging SARS-CoV-2 variants for ensovibep*

Variant	Substitutions / Deletions	Assay Type	Neutralizing IC_{50} [ng/mL]
References	Wuhan wild type	VSV pseudotype	1
	D614G background	Lentivirus pseudotype	1.1
	D614G background	Lentivirus pseudotype Setup 2	2.7
	French isolate: V367F; E990A	Authentic virus	1.3
Alpha / B.1.1.7	69-70 del, del145, N501Y, A570D, D614G, P681H, T716I, S982A, D1118H	VSV	1.7
	69-70 del, del145, E484K, N501Y, A570D, D614G, P681H, T716I, S982A, D1118H	VSV	3.2
	69-70 del, del145, N501Y, A570D, D614G, P681H, T716I, S982A, D1118H	Lentivirus pseudotype	0.9
	69-70 del, del145, S494P, N501Y, A570D, D614G, P681H, T716I, S982A, D1118H	Lentivirus pseudotype	0.8
	H69_V70del, Y145del, N501Y, A570D, D614G, P681H, T716I, S982A, D1118H	Authentic	1.3
Beta / B.1.351	D80A, D215G, E484K, N501Y, A701V	VSV	5.5
	L18F, D80A, D215G, Del242-244, R246I, K417N, E484K, N501Y, D614G, A701V	VSV	5
	L18F, D80A, D215G, Del242-244, K417N, E484K, N501Y, D614G, A701V	Lentivirus pseudotype	1.2
	L18F, D80A, D215G, L242_L244del, T302T, K417N, E484K, N501Y, D571D, D614G, A701V	Authentic	7.5
Gamma / P.1	L18F, T20N, P26S, D138Y, R190S, K417T, E484K, N501Y, D614G, H655Y, T1027I	VSV	1.2
	L18F, T20N, P26S, D138Y, R190S, K417T, E484K, N501Y, D614G, H655Y, T1027I, V1176F	Lentivirus pseudotype	0.7
	L18F, T20N, P26S, D138Y, R190S, K417T, E484K, N501Y, D614G, H655Y, T1027I, V1176F	Authentic	5.7
Delta/DeltaPlus / B.1.617.2	T19R, G142D, E156GI, F157del, R158del, L452R, T478K, D614G, P681R, D950N	Lentivirus pseudotype	2.4
	T19R, T95I, G142D, E156G, F157del, R158del, W258L, K417N, L452R, T478K, D614G, P681R, D950N	Lentivirus pseudotype	2.6
	T19R, T95I, G142D, Y145H, E156G, F157-, R158-, A222V, L452R, T478K, D614G, P681R, D950N	Lentivirus pseudotype (Setup 2)	1.6
Epsilon/B.1.429	S13I, W152C, L452R, D614G	Lentivirus pseudotype	0.9
	S13I, P26S, W152C, L452R, D614G	Lentivirus pseudotype	0.5
Iota/B.1.526	L5F, T95I, D253G, E484K, D614G, A701V	Lentivirus pseudotype	3.0
Kappa / B.1.617.1	T95I, G142D, E154K, L452R, E484Q, D614G, P681R, Q1071H	Lentivirus pseudotype	2.0
	G142D, E154K, V382L, L452R, E484Q, D614G, P681R, Q1071H, D1153Y	Lentivirus pseudotype	1.9
Lambda / C37	G75V, T76I, R246del, S247-G252del, D253N, L452Q, F490S, D614G, T859N	Lentivirus pseudotype	0.4

**Mu / B.1.621**	T95I, Y144S, Y145N, R346K, E484K, N501Y, D614G, P681H, D950N	Lentivirus pseudotype	6.1
**Omicron / B.1.1.529 / BA.1**	A67V, Δ69-70, T95I, G142D, Δ143-145, Δ211, L212I, ins214EPE, G339D, S371L, S373P, S375F, K417N, N440K, G446S, S477N, T478K, E484A, Q493R, G496S, Q498R, N501Y, Y505H, T547K, D614G, H655Y, N679K, P681H, N764K, D796Y, N856K, N969K, L981F	VSV pseudotype	2.2
	A67V, Δ69-70, T95I, G142D, Δ143-145, Δ211, L212I, ins214EPE, G339D, S371L, S373P, S375F, K417N, N440K, G446S, S477N, T478K, E484A, Q493K, G496S, Q498R, N501Y, Y505H, T547K, D614G, H655Y, N679K, P681H, N764K, D796Y, N856K, Q954H, N969K, L981F	VSV pseudotype	2.1
	A67V, Δ69-70, T95I, G142D, Δ143-145, Δ211, L212I, ins214EPE, G339D, S371L, S373P, S375F, K417N, N440K, G446S, S477N, T478K, E484A, Q493R, G496S, Q498R, N501Y, Y505H, T547K, D614G, H655Y, N679K, P681H, N764K, D796Y, N856K, Q954H, N969K, L981F	Lentivirus pseudotype	3.6
**Omicron / B.1.1.529 / BA.2**	T19I, L24-, P25-, P26-, A27S, G142D, V213G, G339D, S371F, S373P, S375F, T376A, D405N, R408S, K417N, N440K, S477N, T478K, E484A, Q493R, Q498R, N501Y, Y505H, D614G, H655Y, N679K, P681H, N764K, D796Y, Q954H, N969K	Lentivirus pseudotype (Setup 2)	2.7
	T19I, L24-, P25-, P26-, A27S, G142D, V213G, G339D, S371F, S373P, S375F, T376A, D405N, R408S, K417N, N440K, S477N, T478K, E484A, Q493R, Q498R, N501Y, Y505H, D614G, H655Y, N679K, P681H, N764K, D796Y, Q954H, N969K	VSV pseudotype	1.5
**B.1.640.1**	P9L_E96Q_C136del_N137del_D138del_P139del_F140del_L141del_G142del_V143del_Y144del_R190S_I210T_R346S_N394S_Y449N_F490R_N501Y_D614G_P681H_T859N_D936H	Lentivirus pseudotype (Setup 2)	0.9
**B.1.640.2**	P9L, E96Q, delC136-Y144, R190S, D215H, R346S, N394S, Y449N, E484K, F490S, N501Y, D614G, P681H, T859N, D1139H	VSV pseudotype	0.6
**R.1**	W152L, E484K, D624G, G769V	Lentivirus pseudotype	2.4
**A.23.1**	F157L, V367F, Q613H, D614G, P681R	Lentivirus pseudotype	0.3

Supplementary Table 3: In vitro *protection against SARS-CoV-2 spike protein substitutions or deletions for ensovibep.*

Amino acid position	Substitution / deletion	Assay Type	Neutralizing IC_{50} [ng/mL]
L18	F	VSV-pseudotype	3.5
T20	N	VSV-pseudotype	4.6
P26	S	VSV-pseudotype	1.8
69-70	del	VSV-pseudotype	1.9
D80	A	VSV-pseudotype	3.6
T95	I	Lentivirus pseudotype	0.9
D138	Y	VSV-pseudotype	2.2
145	del	VSV-pseudotype	2.1
W152	L	Lentivirus pseudotype	1.2
R190	S	VSV-pseudotype	1.7
A222	V	VSV-pseudotype	2.2
N234	Q	VSV-pseudotype	16.2
242-244	del	VSV-pseudotype	2.0
G339	D	Lentivirus pseudotype	2.0
E406	Q	Lentivirus pseudotype	1.5
	W	VSV-pseudotype	2.7
Q409	E	VSV-pseudotype	2.0
K417	E	VSV-pseudotype	0.5
	N	VSV-pseudotype	0.6
	R	VSV-pseudotype	2.1
	T	VSV-pseudotype	0.5
D420	N	VSV-pseudotype	5.6
N439	K	VSV-pseudotype	1.3
K444	E	Lentivirus pseudotype	0.8
	N	VSV-pseudotype	4.4
	Q	Lentivirus pseudotype	1.3
	T	VSV-pseudotype	6.1
V445	A	Lentivirus pseudotype	1.3
G446	V	VSV-pseudotype	1.7
	S	Lentivirus pseudotype	1.3
N450	D	Lentivirus pseudotype	0.9

L452	R	Lentivirus pseudotype	0.4
Y453	F	VSV-pseudotype	3.2
L455	F	Lentivirus pseudotype	1.1
F456	L	VSV-pseudotype	3.1
N460	S	VSV-pseudotype	2.6
	T	VSV-pseudotype	0.6
Y473	H	VSV-pseudotype	2.6
A475	V	Lentivirus pseudotype	0.9
G476	S	VSV-pseudotype	1.5
S477	N	VSV-pseudotype	1.9
T478	I	VSV-pseudotype	2.7
	K	Lentivirus pseudotype	1.5
P479	S	VSV-pseudotype	2.1
V483	A	VSV-pseudotype	2.3
E484	A	Lentivirus pseudotype	2.4
	K	VSV-pseudotype	2.7
	Q	Lentivirus pseudotype	2.3
G485	D	VSV-pseudotype	28.5
F486	V	VSV-pseudotype	>100
	L	VSV-pseudotype	>100
Y489	H	VSV-pseudotype	>100
F490	S	VSV-pseudotype	3.8
Q493	K	VSV-pseudotype	7.9
	R	VSV-pseudotype	2.2
S494	P	Lentivirus pseudotype	1.3
N501	Y	VSV-pseudotype	0.6
A570	D	VSV-pseudotype	1.2
D614	G	VSV-pseudotype	2.4
H655	Y	VSV-pseudotype	2.4
P681	H	VSV-pseudotype	1.5
A701	V	VSV-pseudotype	1.1
T716	I	VSV-pseudotype	0.6
S982	A	VSV-pseudotype	2.0
T1027	I	VSV-pseudotype	3.3
D1118	H	VSV-pseudotype	2.6

Supplementary Table 4: drug exposure levels in serum at day of euthanization. Animals with drug exposure levels below 10% of the group average were removed from the study analysis (depicted in bold).

**Ensovibep** (10 mg/kg i.p.; 0 h p.i)		
#	Animal ID	Serum concentration [µg/mL]
1	DN5_1	86.9
**2**	**DN5_2**	**2.7**
3	DN5_3	169.7
4	DN5_4	44.5
5	DN5_5	92.9
6	DN5_6	39.2
**7**	**DN5_7**	**1.5**
8	DN5_8	109.4
9	DN5_9	51.8
10	DN5_10	127.9
11	DN5_11	70.2
12	DN5_12	38.6

**Ensovibep** (10 mg/kg i.p.; 24 h p.i)		
#	Animal ID	Serum concentration [µg/mL]
1	DN5_25	17.0
**2**	**DN5_26**	**1.8**
3	DN5_27	39.1
4	DN5_28	N/A
5	DN5_29	48.0
**6**	**DN5_30**	**5.7**
7	DN5_31	225.1
8	DN5_32	27.3
9	DN5_33	66.6
10	DN5_34	109.5
11	DN5_35	128.4
12	DN5_36	78.3

**REGN10933 & REGN10987** (5 + 5 mg/kg i.p.; 0 h p.i)		
#	Animal ID	Serum concentration [µg/mL]
1	DN5_13	29.0
**2**	**DN5_14**	**0.3**
3	DN5_15	26.5
**4**	**DN5_16**	**1.1**
5	DN5_17	34.0
**6**	**DN5_18**	**1.4**
7	DN5_19	41.9
8	DN5_20	30.3
9	DN5_21	43.5
10	DN5_22	38.0
11	DN5_23	28.1
12	DN5_24	32.7

**REGN10933 & REGN10987** (5 + 5 mg/kg i.p.; 24 h p.i)		
#	Animal ID	Serum concentration [µg/mL]
1	DN5_37	72.0
2	DN5_38	60.9
3	DN5_39	42.8
4	DN5_40	32.6
5	DN5_41	46.7
6	DN5_42	41.9
7	DN5_43	43.7
8	DN5_44	45.3
9	DN5_45	46.1
10	DN5_46	61.9
11	DN5_47	37.2
12	DN5_48	41.8

N/A: Not available due to low amount of serum extracted from terminal bleeds

Bold: animals removed from the study data due to low therapeutic exposure

Red: animals euthanized at 2 dpi

Blue: animals euthanized at 3 dpi

Green: animals euthanized at 5dpi

Supplementary Table 5: Identification of escape mutations by deep sequencing of SARS-CoV-2 Alpha variant B.1.1.7 in animals at day 5 p.i., which indicated remaining viral titers. As a control, three non-treated animals were also deep sequenced. Deep Sequencing was performed from either swab (S) or lung (L) extracted RNA.

Treatment group	Animal identifier	Throat swab (S) or lung homogenate (L)	Identified spike protein amino acid substitution	Potential Impact
**Ensovibep 0 dpi**	DN5_2	L	-	-
		S	-	-
	DN5_4	L	-	-
		S	K1034M	Neutral / outside ensovibep epitope
**antibody cocktail 0 dpi**	DN5_15	L	-	-
		S	-	-
	DN5_24	L	-	-
		S	-	-
**Ensovibep 1 dpi**	DN5_30	L	-	-
		S	-	-
**Placebo group**	DN5_50	L	-	-
		S	R671L	At furin cleavage site
	DN5_52	L	-	-
		S	R671L	At furin cleavage site
	DN5_53	L	-	-
		S	-	-

Supplementary Table 6: *Histopathology data table*

Stimulus	Necropsy day post infection	Inflammation Parameters					Bronchi			Alveoli			Vasculature		
		% affected	Degree of inflammation	Lymphocytes	Macrophages	Neutrophils	Broncho-epithelial necroses	Bronchitis	Broncho-epithelial hyperplasia	Alveolar epithelium necroses	Alveolar edema	Typ II Hyperplasia	Perivascular lymphocyte cuffs	Perivascular edema	Endothelitis
PBS / non-infected	3	20	1	0	1	1	0	1	0	0	1	0	0	1	0
PBS / non-infected	3	<5	0	0	0	0	0	0	0	0	0	0	0	0	0
PBS / non-infected	5	<5	0	0	0	0	0	0	0	0	0	0	0	0	0
PBS / non-infected	5	<5	0	0	0	0	0	0	0	0	0	0	0	0	0
PBS / non-infected	5	10	1	1	1	1	0	0	1	0	0	1	0	1	0
PBS / non-infected - Excluded from analysis	3	90	2	2	2	2	0	1	0	1	2	0	3	2	0
PBS / Infected	2	80	1	0	1	1	1	1	1	1	2	0	0	3	1
PBS / Infected	2	80	3	1	3	3	1	2	1	1	3	0	0	2	0
PBS / Infected	2	80	3	1	2	3	1	2	1	1	2	0	0	2	2
PBS / Infected	2	70	1	1	1	1	1	1	0	1	2	0	0	2	0
PBS / Infected	3	70	3	1	3	3	1	3	1	0	2	1	0	2	2
PBS / Infected	3	70	2	2	2	2	1	2	0	0	3	1	2	2	2
PBS / Infected	3	20	1	1	1	1	1	2	0	1	2	0	0	1	1
PBS / Infected	5	40	3	2	2	2	0	2	1	0	0	2	0	1	2
PBS / Infected	5	60	3	2	2	2	0	2	0	0	1	2	2	1	3
PBS / Infected	5	50	2	2	1	1	0	1	2	0	0	2	2	1	0
PBS / Infected	5	30	1	2	1	1	0	1	1	0	0	2	1	1	3
PBS / Infected - excluded from analysis	2	100	0	0	0	0	0	0	1	0	0	0	0	0	0
ensovibep 10mg/kg (0dpi)	3	50	2	1	2	2	1	1	1	0	1	2	4	1	0
ensovibep 10mg/kg (0dpi)	3	40	3	1	2	3	1	2	2	1	2	0	0	1	1
ensovibep 10mg/kg (0dpi)	3	40	3	1	2	2	1	3	1	1	2	1	1	1	0
ensovibep 10mg/kg (0dpi)	3	5	1	0	0	1	1	0	0	0	1	0	0	0	0
ensovibep 10mg/kg (0dpi)	3	5	±	0	0	0	0	0	0	0	0	0	0	0	0
ensovibep 10mg/kg (0dpi)	5	90	3	1	3	3	1	2	2	1	2	3	2	1	0
ensovibep 10mg/kg (0dpi)	5	50	2	1	2	2	1	2	2	0	1	3	1	1	0
ensovibep 10mg/kg (0dpi)	5	15	1	1	1	1	1	1	1	0	1	2	4	0	0
ensovibep 10mg/kg (0dpi)	5	50	3	1	2	2	1	2	2	0	1	2	1	1	0
ensovibep 10mg/kg (0dpi)	5	<5	0	0	0	0	0	0	0	0	0	0	0	0	0
ensovibep 10mg/kg (1dpi)	3	100	1	0	1	1	0	0	0	3	2	0	0	3	0
ensovibep 10mg/kg (1dpi)	3	20	1	1	1	1	1	1	3	0	1	1	0	0	1
ensovibep 10mg/kg (1dpi)	3	<5	0	0	0	0	0	0	0	0	0	0	0	0	1
ensovibep 10mg/kg (1dpi)	3	100	1	0	1	1	1	1	0	2	2	1	0	3	2
ensovibep 10mg/kg (1dpi)	3	80	2	0	2	2	1	2	2	2	1	1	0	2	2
ensovibep 10mg/kg (1dpi)	5	<5	0	0	0	0	0	0	0	0	0	0	0	0	0
ensovibep 10mg/kg (1dpi)	5	20	1	1	1	1	0	1	1	0	0	1	1	1	0
ensovibep 10mg/kg (1dpi)	5	100	2	1	2	2	3	1	1	3	3	0	0	1	0
ensovibep 10mg/kg (1dpi)	5	80	2	1	2	2	1	2	1	1	2	2	1	0	0
ensovibep 10mg/kg (1dpi)	5	<5	1	0	1	1	0	0	0	0	0	1	0	0	0
mAb cocktail 10mg/kg (0dpi)	3	70	3	2	2	2	1	1	2	1	1	1	2	1	1
mAb cocktail 10mg/kg (0dpi)	3	90	2	1	2	2	1	1	1	0	0	1	0	1	0
mAb cocktail 10mg/kg (0dpi)	3	30	1	1	1	1	0	1	1	0	0	1	0	1	0
mAb cocktail 10mg/kg (0dpi)	3	50	2	1	2	2	1	2	2	0	2	1	0	1	0
mAb cocktail 10mg/kg (0dpi)	5	<5	0	0	0	0	0	0	0	0	0	0	0	0	0
mAb cocktail 10mg/kg (0dpi)	5	50	3	1	3	3	1	3	1	1	2	1	1	1	1
mAb cocktail 10mg/kg (0dpi)	5	10	1	1	1	1	0	1	1	0	0	1	0	0	0
mAb cocktail 10mg/kg (0dpi)	5	<5	1	0	1	1	0	0	0	0	0	0	0	0	0
mAb cocktail 10mg/kg (0dpi)	5	30	1	1	1	1	0	1	2	0	0	1	0	1	0
mAb cocktail 10mg/kg (1dpi)	2	50	3	1	3	3	1	3	1	2	3	0	0	1	1
mAb cocktail 10mg/kg (1dpi)	2	90	3	0	3	3	1	3	1	1	2	0	0	2	1
mAb cocktail 10mg/kg (1dpi)	2	90	3	1	3	3	1	1	0	1	1	0	2	3	2
mAb cocktail 10mg/kg (1dpi)	2	90	3	0	3	3	1	2	1	1	3	0	0	3	0
mAb cocktail 10mg/kg (1dpi)	2	20	2	0	2	2	1	2	1	1	1	0	0	2	0
mAb cocktail 10mg/kg (1dpi)	3	60	3	0	3	3	1	2	0	1	3	1	1	2	1
mAb cocktail 10mg/kg (1dpi)	5	40	1	1	1	1	0	1	3	0	0	3	0	1	0
mAb cocktail 10mg/kg (1dpi)	5	80	1	1	1	1	0	1	3	0	1	3	0	1	0
mAb cocktail 10mg/kg (1dpi)	5	70	1	1	1	1	0	0	1	0	1	3	0	1	0
mAb cocktail 10mg/kg (1dpi)	5	10	1	1	0	0	0	0	1	0	0	1	0	1	0
mAb cocktail 10mg/kg (1dpi)	5	10	1	1	1	1	0	0	0	0	0	1	0	1	0
mAb cocktail 10mg/kg (1dpi)	5	<5	1	1	1	1	0	0	2	0	0	1	0	1	0

References

1 Steiner, D. *et al.* Half-life extension using serum albumin-binding DARPin(R) domains. *Protein Eng Des Sel* **30**, 583-591, doi:10.1093/protein/gzx022 (2017).

2 Walser, M. *et al.* Highly potent anti-SARS-CoV-2 multivalent DARPin therapeutic candidates. *bioRxiv* (2020).

3 Friedrich, M. *et al.* Preclinical characterization of AMG 330, a CD3/CD33-bispecific T-cell-engaging antibody with potential for treatment of acute myelogenous leukemia. *Mol Cancer Ther* **13**, 1549-1557, doi:10.1158/1535-7163.MCT-13-0956 (2014).
