# Supplementary material for: The trispecific DARPin ensovibep inhibits diverse SARS-CoV-2 variants
Source: Nat Biotechnol. 2022 Jul 21;40(12):1845–54. doi: 10.1038/s41587-022-01382-3 (PMC9750863; doi:10.1038/s41587-022-01382-3)
Supplement: Supplementary file 2 — Reporting Summary [file 41587_2022_1382_MOESM2_ESM.pdf]

nature research

Corresponding author(s): Michael T. Stumpp

Last updated by author(s): May 24, 2022

Reporting Summary

Nature Research wishes to improve the reproducibility of the work that we publish. This form provides structure for consistency and transparency in reporting. For further information on Nature Research policies, see our Editorial Policies and the Editorial Policy Checklist.

Statistics

For all statistical analyses, confirm that the following items are present in the figure legend, table legend, main text, or Methods section.

n/a	Confirmed	
☐	☒	The exact sample size (*n*) for each experimental group/condition, given as a discrete number and unit of measurement
☐	☒	A statement on whether measurements were taken from distinct samples or whether the same sample was measured repeatedly
☐	☒	The statistical test(s) used AND whether they are one- or two-sided<br>*Only common tests should be described solely by name; describe more complex techniques in the Methods section.*
☒	☐	A description of all covariates tested
☒	☐	A description of any assumptions or corrections, such as tests of normality and adjustment for multiple comparisons
☐	☒	A full description of the statistical parameters including central tendency (e.g. means) or other basic estimates (e.g. regression coefficient) AND variation (e.g. standard deviation) or associated estimates of uncertainty (e.g. confidence intervals)
☐	☒	For null hypothesis testing, the test statistic (e.g. *F*, *t*, *r*) with confidence intervals, effect sizes, degrees of freedom and *P* value noted<br>*Give P values as exact values whenever suitable.*
☒	☐	For Bayesian analysis, information on the choice of priors and Markov chain Monte Carlo settings
☒	☐	For hierarchical and complex designs, identification of the appropriate level for tests and full reporting of outcomes
☒	☐	Estimates of effect sizes (e.g. Cohen's *d*, Pearson's *r*), indicating how they were calculated

Our web collection on statistics for biologists contains articles on many of the points above.

Software and code

Policy information about availability of computer code

Data collection	Cryo-EM data were acquired using the EPU 2 software (Thermo Fisher Scientific). Deep sequencing after viral passaging was done using the Ion Chef Instrument (ThermoFisher Scientific). Hamsters were uniquely identified with subcutaneously implemented IPTT-300 transponders (BMDS, Seaford, DE, USA). Body temperatures were recorded using the DAS-8027-IUS reader with DASHost communications software (BMDS, Seaford, DE, USA). Whole-genome sequencing after the hamster experiment was performed on a Illumina MiSeq using v3 chemistry for 2x300 bp reads. Bulk RNA sequencing was performed after RNA isolation of the right medium lung lobe. Bulk RNA sequencing libraries were constructed using the NEBNext Ultra II Directional RNA Library Prep Kit (New England Biolabs) and sequenced on a Illumina NextSeq 500 device.
Data analysis	Cryo-EM data were processed using Relion (version 3.1.1), MotionCor2 (version 1.3.0), GCTF (version 1.06), DeepEMhancer (version 0.13) UCSF Chimera (version 1.15.0), UCSF ChimeraX (version 1.2.5) and COSMIC2 web platform. Deep sequencing data were processed using Samtools 1.10, Trimmomatic 0.39, bwa 0.7.17, LoFreq v2.1.5, bcftools 1.10, SNPEff 5.0, and R 3.6.1. Whole-genome sequencing data were processed using the same software packages as described above. For viral transcriptome analysis, total RNA-seq reads were mapped to the SARS-CoV-2 genome (GenBank MN908947). For host response transcriptome analysis, reads were aligned to the Roborowski hamster genome with hisat2 and gene expression quantified using the package featureCounts from Rsubread and analyzed by DESeq2.

For manuscripts utilizing custom algorithms or software that are central to the research but not yet described in published literature, software must be made available to editors and reviewers. We strongly encourage code deposition in a community repository (e.g. GitHub). See the Nature Research guidelines for submitting code & software for further information.

Data

Policy information about availability of data

All manuscripts must include a data availability statement. This statement should provide the following information, where applicable:

- Accession codes, unique identifiers, or web links for publicly available datasets
- A list of figures that have associated raw data
- A description of any restrictions on data availability

The EM density maps for the SARS-CoV-2 spike ectodomain in complex with monovalent DARPin R2 have been deposited to the Electron Microscopy Data Bank under the accession codes EMD-11953, EMD-14810, EMD-14811 and EMD-11954. The atomic coordinates of the SARS-CoV-2 spike ectodomain used to generate the starting model for cryo-EM 3D classification are available from the Protein Data Bank under accession code PDB ID: 6VSB. The atomic coordinates of the template used to generate DARPin R1, R2 and R3 are available from the Protein Data Bank under accession code PDB ID: 2XEE. The monovalent DARPin and multivalent DARPin sequences, and pseudo-atomic models derived from molecular docking experiments, are available here, to allow the use of the data for non-commercial purposes: https://www.guidetopharmacology.org/GRAC/LigandDisplayForward?tab=structure&ligandId=11470

Field-specific reporting

Please select the one below that is the best fit for your research. If you are not sure, read the appropriate sections before making your selection.

☒ Life sciences ☐ Behavioural & social sciences ☐ Ecological, evolutionary & environmental sciences

For a reference copy of the document with all sections, see nature.com/documents/nr-reporting-summary-flat.pdf

Life sciences study design

All studies must disclose on these points even when the disclosure is negative.

Sample size	For hamster studies, we chose the sample size based on previous experience with SARS-CoV-2 infection in this particular animal model. Since Roborovski dwarf hamsters can develop severe disease upon SARS-CoV-2 challenge and are highly permissive to virus replication, relevant differences can be identified with comparatively few individuals per group. The use of N=12 animals with two planned take-outs (planned N=6 per time-point) is based on previous experiments that provided statistically significant differences even with smaller groups sizes. Relevant citations can be found here: 10.1016/j.celrep.2020.108488; 10.1038/s41586-021-03995-1; 10.1016/j.ymthe.2022.03.014
Data exclusions	Few animals were excluded from the analyses if intraperitoneal injections were suboptimal and accordingly described in the manuscript and supplementary material (Figure 5d-f, Supplementary Figure 13, and Supplementary Table 4). Animals where organ removal was compromised were also excluded from histology analysis as described (Supplementary Table 6)
Replication	For the purpose of this publication, two independent experiments regarding the treatment of SARS-CoV-2 infection with ensovibep were performed. First, we performed a dose finding study to confirm the efficacious ensovibep dose, this data is presented in the supplementary information of the manuscript. Second, we performed a comparison of ensovibep and the Regerneron mAb cocktail at the previously determined dose. The outcome of both experiments is in good agreement. Ethical considerations clearly mandate the prudent use of animals for scientific purposes, considering the already large number of animals used in two independent experiments performed for this publication, and made us desist from further replications. Additionally, permission for replication of animal experiments is unlikely to be granted under German law.
Randomization	Hamsters were randomized and stratified for body weight.
Blinding	Treatment, scoring of the health status as well as histology scoring was performed by blinded operators using unique sample identifiers.

Reporting for specific materials, systems and methods

We require information from authors about some types of materials, experimental systems and methods used in many studies. Here, indicate whether each material, system or method listed is relevant to your study. If you are not sure if a list item applies to your research, read the appropriate section before selecting a response.

Materials & experimental systems

n/a	Involved in the study	
☐	☒	Antibodies
☐	☒	Eukaryotic cell lines
☒	☐	Palaeontology and archaeology
☐	☒	Animals and other organisms
☒	☐	Human research participants
☒	☐	Clinical data
☒	☐	Dual use research of concern

Methods

n/a	Involved in the study	
☒	☐	ChIP-seq
☒	☐	Flow cytometry
☒	☐	MRI-based neuroimaging

Antibodies

Antibodies used	Publicly available sequences of variable domains from monoclonal antibodies were used to synthetize the corresponding cDNA fragments and cloned into a proprietary expression vector at Evitria AG (Schlieren, Switzerland)
Validation	Purity and specificity of antibodies was confirmed using chromatographic techniques, and ELISA using SARS-CoV-2 spike protein, respectively.

Eukaryotic cell lines

Policy information about cell lines

Cell line source(s)	Vero E6 cells (kindly provided by Prof. Volker Thiel, University of Bern, Switzerland). Vero E6/TMPRSS2 cells were obtained from the Centre For AIDS Reagents (National Institute for Biological Standards and Control, Herts, UK). HEK293T-ACE2.TMPRSS2s cells were generated in the Carol Weiss lab (US Food and Drug Administration, Center for Biologics Evaluation and Research) as described.
Authentication	None of the cell lines were authenticated.
Mycoplasma contamination	Mycoplasma was tested and confirmed negative using PCR.
Commonly misidentified lines (See ICLAC register)	None

Animals and other organisms

Policy information about studies involving animals; ARRIVE guidelines recommended for reporting animal research

Laboratory animals	Female and male outbread Roborovski dwarf hamsters (Phodopus roborovskii) of 6-9 weeks of age (13-25 g) were used as supplied by German Pet trade. A strain designation is not available, these animals are a non-traditional animal model and not bred for scientific purposes in Europe. Importantly, hamsters of this species show a similarly severe COVID-19 like disease phenotype upon SARS-CoV-2 infection when obtained from different sourced across different geographic origins: 10.1080/21505594.2021.1972201; 10.1016/j.celrep.2022.110502
Wild animals	None
Field-collected samples	None
Ethics oversight	All animal procedures were performed in accordance with relevant institutional and legal regulations and approved by the responsible state authority, Landesamt für Gesundheit und Soziales Berlin, Germany, permit number G 0086/20. Under German law, all studies involving animals have to be approved by the relevant state authority after review by an ethics committee (German: Tierversuchskommission, according to § 15 Abs. 1 Tierschutzgesetz and § 42 Tierschutz-Versuchstierordnung). The state authority (in our case, Landesamt für Gesundheit und Soziales Berlin) is solely responsible for approval and issues the permit (in this case, G0086/20). It is not the ethics committee, but the authority that finally approves the study.

Note that full information on the approval of the study protocol must also be provided in the manuscript.
